# Motherhood choice in multiple sclerosis (MoMS) – Pilot trial of web-based decision support

**DOI:** 10.1371/journal.pone.0351108

**Published:** 2026-06-12

**Authors:** Lara S. Stahl, Julia Hickstein, Sascha Köpke, Kerstin Hellwig, Christoph Heesen, Anne C. Rahn

**Affiliations:** 1 Institute for Neuroimmunology and Multiple Sclerosis, University Medical Centre Hamburg-Eppendorf, Hamburg, Germany; 2 Institute for Social Medicine and Epidemiology, Nursing Research Unit, University of Lübeck, Lübeck, Germany; 3 Institute of Nursing Science, University of Cologne, Medical Faculty and University Hospital Cologne, Cologne, Germany; 4 Department of Neurology, St. Josef Hospital Bochum, Ruhr University Bochum, Bochum, Germany; 5 Department of Neurology, University Medical Centre Hamburg-Eppendorf, Hamburg, Germany; Ministry of Health, Sri Lanka, SRI LANKA

## Abstract

**Backgroud:**

Uncertainty concerning motherhood is common among women with multiple sclerosis (wwMS). Therefore, we developed and pre-tested a patient decision aid (PtDA) and a nurse-led decision coaching intervention (DC) to support motherhood choice. The DC includes the PtDA, a decision guide on motherhood choice, and decision coaching.

**Methods:**

We conducted a randomised pilot trial across Germany to test feasibility, with decisional conflict (Decisional Conflict Scale, DCS) as an exploratory endpoint. Initially, we planned a 3:1 randomisation ratio (planned; PtDA = 48, DC = 16). Due to recruitment difficulties, we switched to a 1:1 randomisation ratio to ensure an appropriate sample size (PtDA > 20, DC > 10). Women between 18 and 45 years old with relapsing-remitting MS or clinically isolated syndrome, and who had not yet decided about motherhood, were eligible. We recruited two nurses for our decision coaching training course. We used questionnaires to measure decisional conflict, programme feasibility, knowledge, and worries regarding pregnancy in MS. Interviews were conducted to gain in-depth information on the potential feasibility of the programmes. Interviews were analysed thematically and questionnaires descriptively. We conducted explorative group comparisons and merged findings using joint display analysis.

**Results:**

We trained two decision coaches in the DC group and recruited 35 wwMS (PtDA = 22; DC = 13) over five months. Median DCS scores in the DC group were 49 at baseline and 15 at follow-up (range 0–100; higher scores indicate greater decisional conflict). In the PtDA group, scores were 54 (baseline) and 31 (follow-up). Explorative group comparison indicated lower decisional conflict at follow-up in the DC group than in the PtDA group (p = 0.035). Interviewees (PtDA = 5; DC = 6; nurses = 2) described both interventions as helpful for decision-making. Qualitative findings indicate greater satisfaction levels in the DC group.

**Conclusion:**

Both interventions appear useful for wwMS in making motherhood choices. The DC programme seems more promising in supporting decision-making.

**Trial registration:**

German Clinical Trials Register (DRKS); DRKS00038534.

## Introduction

Multiple sclerosis (MS) is the most common autoimmune disease of the central nervous system and is predominantly diagnosed in women of childbearing age [1]. Many women start their family planning around the time of the MS onset [2]. MS itself has no adverse effects on the course of pregnancy [3,4], and recent studies showed no negative impact of pregnancy on the long-term disease course of MS or children of women with MS (wwMS) [3,4]. An MS diagnosis, however, can lead to insecurities and anxieties regarding family planning decisions [5,6], especially concerning immunotherapy. Reliable and comprehensive information can be difficult to obtain for wwMS [7], and misinformation and uncertainties are common among wwMS and healthcare providers [5].

One approach to support informed decision-making about pregnancy is shared decision-making (SDM). SDM is a structured approach that supports health decisions by enabling patients and healthcare professionals to consider options together, discuss risks and benefits, and incorporate personal values and preferences to make an informed choice [8]. Patient decision aids (patient decision aid(s), PtDA) support SDM by providing information and clarifying preferences and values [9]. These are often based on evidence-based patient information (EBPI), which offer clear, objective, and transparent information on diseases, diagnostics, treatments, and prevention [10]. Nearly 20 years ago, Prunty et al. evaluated a PtDA on motherhood choice for wwMS in a randomised controlled trial (RCT) with 194 wwMS [11]. The PtDA increased knowledge and reduced decisional conflict regarding motherhood choice, but was last updated in 2011 and thus does not contain recent findings, such as those on newer treatment options [11].

Given the complexity of family-planning decisions, decision coaching could be an additional assistance to a PtDA. Decision coaching is a non-directive form of support by trained health professionals to provide individual decision support, explain health information, clarify preferences, and encourage their sharing [12]. A cluster randomised trial (DECIMS, decision coaching in multiple sclerosis) found indications that nurse-led decision coaching, including EBPI, is helpful and acceptable in supporting people with MS in decision-making processes [13]. To date, there is only one support programme for motherhood in MS in Germany [14], the effectiveness of which has not yet been evaluated, and which does not include a structured decision coaching programme.

Our study aimed to pilot-test two support programmes on motherhood choice for wwMS considering pregnancy: 1) a PtDA and 2) a decision coaching programme including a PtDA. We evaluated whether a PtDA alone or combined with a decision coaching programme is the most promising approach.

## Materials and methods

This study is a part of the multiphase *MoMS* study (*Motherhood choice in multiple sclerosis)*, a mixed-methods study [15] based on the British Medical Research Council’s (MRC) framework for developing and evaluating complex interventions, addressing phases one (needs assessment and development) and two (see S1 Fig – Overview of the study steps) [16]. In phase two, both programmes were tested and piloted for feasibility using the alpha and beta testing approach by Coulter et al. [17]. This article primarily focuses on the pilot testing (beta testing) conducted in phase two. Further details on the development process and the feasibility testing (alpha testing) are provided in S2 Text and S3 Text. This pilot trial was retrospectively registered in the German Clinical Trials Register (DRKS, registration number DRKS00038534) on 1 December 2025. We did not prospectively register it because the pilot study was conceived as an exploratory feasibility trial, and at that time we did not consider it to fall under the requirements of a full clinical trial.

### Decision support programmes

We developed two support programmes on motherhood choice for wwMS considering pregnancy: 1) a PtDA and 2) a decision coaching programme including a PtDA. Supplementary Table S2 (see S2 Text) provides an overview of the components following the TIDieR guidelines [18]. For a detailed description of the development process, see S2 Text.

#### Patient decision aid (PtDA).

We developed a web-based PtDA covering the different aspects of MS and motherhood (see S2 Text) and a workbook (decision guide, DG). The DG complements the PtDA and contains six steps of SDM [13,19].

#### Decision coaching programme.

The decision coaching programme consists of a training course for nurses and the decision coaching intervention. The decision coaching intervention (abbreviated as DC) entails the PtDA, a modified DG (DG^pro^), a decision coaching session with a trained nurse, and moderation cards for the nurses. Compared to the DG, the DGpro used in the decision coaching sessions, contains additional information on prognostic factors for the MS course and the opportunity to document the individual MS course together with the nurse. We consider it essential for this section of the guide to be completed with the trained nurse, as it could leave women feeling uncertain and anxious.

#### Feasibility testing/ alpha testing.

Health professionals with experience caring for wwMS who are considering pregnancy, a patient representative, and wwMS were recruited to assess the practicability and acceptance of the PtDA and DG iteratively. Participants filled out questionnaires, and semi-structured interviews were performed by one author (JH) to explore usability. We tested decision coaching and the moderation cards within the research team, with an independent MS nurse from the *University Medical Centre Hamburg-Eppendorf* (UKE) and a wwMS. Quantitative and qualitative data indicate good acceptance and practicability of the components. During feasibility testing, we continuously refined each component based on the feedback. For a detailed description, see S3 Text.

### Randomised pilot trial (beta testing)

We conducted a randomised parallel-group pilot study [20] with a process evaluation [21] to gain in-depth information on their feasibility and identify potential for improvement. The pilot study followed the CONSORT extension to pilot and feasibility trials [22] (see S7 Table for the CONSORT checklist). The study took place across Germany with location-independent participation.

#### Decision coaches.

For the decision coaching programme, we recruited and trained one nurse from the MS outpatient clinic at the *UKE* and one from the MS outpatient clinic at the *University Medical Center of the Ruhr University Bochum* as decision coaches. After the training, they conducted and audio-recorded one (Coach 2) and two coaching sessions (Coach 1) as training on the job with wwMS, considering pregnancy. After these training sessions, the nurses received feedback from JH. Before and after the training course, evaluation and knowledge questionnaires were completed to assess the impact of the training. We developed the knowledge questionnaire based on the final quiz of the Ottawa Decision Support Tutorial [23]. One nurse performed decision coaching via telephone, and the other via a web conference to determine which medium should be used. During the pilot study, nurses completed questionnaires after each coaching session (see Measured outcomes and process evaluation).

#### Participants and recruitment.

We defined the following inclusion criteria: women had to be aged between 18 and 45 years with relapsing-remitting multiple sclerosis (RRMS), clinically isolated syndrome (CIS), or suspected RRMS, and who had not yet fully decided about motherhood. Women with MS who were not facing a decision regarding family planning in connection with their MS were excluded. We recruited wwMS via flyers in MS outpatient clinics, mailing lists, and social media outlets where self-help organisations and wwMS advertised our study. Recruitment took place from 20 September 2021 to 28 February 2022. The employees of the MS outpatient clinics did not perform recruitment. Interested women contacted the study team, and one researcher checked the inclusion criteria via phone. After the wwMS gave written informed consent, they were randomised to receive the PtDA or decision coaching with PtDA (abbreviated as DC).

#### Randomisation.

We did not conduct a formal sample size calculation due to the exploratory design of our study. For intermediate effect sizes between 0.4 and 0.6 and an 80% confidence level, Sim and Lewis recommend a minimum sample size of 25–35 participants for pilot studies [24]. We aimed for a sample size of 64 participants (PtDA = 48, DC = 16) applying a 3:1 randomisation (block sizes of 16) to account for dropouts and loss to follow-up. Due to the higher demands and additional resources required for the DC (e.g., the coaches’ time resources), we intended to recruit a smaller DC group. We had initially intended a 3:1 randomisation (block sizes of 16). Halfway through the recruitment phase, we changed to a 1:1 randomisation (block sizes of 2, 4, and 6) due to recruitment difficulties to ensure an appropriate number of participants in both groups (PtDA n > 20, DC n > 10). An independent study nurse at the UKE performed the block randomisation by using an online randomisation tool [25]. The study nurse also conducted the allocation and sent all participants the access information to the PtDA and the respective DG via mail. Researchers were not blinded to intervention allocation.

#### Study procedure.

Both groups had access to the programme for two weeks. The decision coaching session took place during this period and was conducted via telephone (Nurse 1) or the web conference tool Cisco Webex (Nurse 2). We asked both groups to complete questionnaires before starting the programme and after two weeks.

#### Clinical data.

Maternity status, disease-specific and sociodemographic data were collected at baseline. We used the Patient-Determined Disease Steps (PDDS) questionnaire [26] to assess the degree of disability. We used a modified version of the Control Preference Scale (CPS) [27] to assess preferences for involvement in decisions regarding immunotherapies when considering pregnancy. For this purpose, we specified the medical decision scenario for MS treatment decisions in the family planning phase.

#### Measured outcomes and process evaluation.

As main outcomes of this study, we evaluated the feasibility and acceptability of the programmes and the potential for expansion into an efficacy trial. We assessed feasibility in accordance with the MRC framework for development and evaluation of complex interventions [16] and the MRC framework for process evaluation of complex interventions [21]. Outcomes were measured at baseline and at follow-up (after two weeks). The exploratory key endpoint was decisional conflict, as measured by the Decisional Conflict Scale (DCS) [28]. The DCS total score is made up of five subscales: (Un)certainty when making a decision, how effective the decision-making is, and three modifiable factors such as feeling uninformed or unsupported as well as value clarity [28]. The DCS ranges from 0 to 100 and is widely used to assess comfort in the decision-making process. Higher DCS scores indicate higher decisional conflict. Scores <25 are associated with implementing decisions, and scores >37.5 are associated with delaying decisions [28,29]. In both groups, we measured the decisional conflict from the perspective of the wwMS. For the decision coaching programme, decisional conflict was additionally measured from the perspective of the decision coach. We measured knowledge regarding pregnancy and MS using the Motherhood choice knowledge questionnaire (MCKQ), which contains 16 questions about pregnancy with MS-related topics [30]. We assessed worries about motherhood using the Motherhood/Pregnancy choice and worries questionnaire (MPWQ, [31]), which comprises the worries scale and descriptive additional items on immunotherapies, attitudes, and coping strategies related to motherhood choice in MS.

For the DC group, we used the Multifocal Approach to the Sharing in Shared Decision-Making (MAPPIN’SDM) [32] to measure SDM. The MAPPIN’SDM assessment was based on audio recordings (observer) of the decision coaching sessions and questionnaires completed by wwMS and decision coaches. The MAPPIN’SDM scale ranges from 0 to 4 and allows the calculation of convergent validities between the involvement of different parties (wwMS and MS nurse). A score of 4 indicates the highest levels of shared decision-making. One author conducted the observer-based assessment based on audio recordings.

Within the scope of the process evaluation, both groups filled out process evaluation questionnaires addressing the feasibility of the programmes at baseline and follow-up. As part of the feasibility testing, we tested the practicability (e.g., length, structure and applicability) and acceptance (e.g., information content and usability) of the components of the programmes [33].

The nurses were asked to complete a questionnaire before and after the training, which included knowledge questions about the training content and feedback questions about the course. After each decision coaching session, the nurses completed an evaluation questionnaire. We interviewed the nurses and some wwMS to gain in-depth insights into the feasibility by using semi-structured interview guides. The results of the evaluation questionnaires were used to develop the interview guides and to choose the wwMS. We aimed to get a range of interviewees with differing perceptions of the programmes’ usefulness. Nurses were interviewed after the last decision coaching session and wwMS after completing the programme.

#### Analysis.

Demographic and quantitative data from the questionnaires (evaluation questionnaires, DCS, MCKQ, MPWQ, MAPPIN’SDM) were analysed descriptively. We summarised continuous data using medians, ranges, means, and standard deviations. Categorical data were reviewed using frequencies. If more than 20% of the items of the DCS, MPWQ, or MAPPIN’SDM were not answered, we excluded the questionnaire from the analysis. If fewer than 20% of the items were left unanswered, we used item mean imputation to replace missing values. For the MCKQ, if fewer than 20% of the items were left unanswered, we considered the unanswered questions to be incorrect. Multiple answers were also marked as incorrect. We exploratively compared the two groups regarding decisional conflict (DCS), knowledge (MCKQ), and worries (MPWQ) using the Mann-Whitney U test and reported the effect size as *r* = standardised test statistic/ total sample size. Results with p < 0.05 (two-sided) were considered statistically significant. We used the following recommendations for interpreting the effect size: small = r ≤ 0.1, moderate ≥0.3, strong ≥0.5 [34]. For statistical data analysis, we used the IBM SPSS Statistics programme version 27.0 [35]. The anonymised dataset (quantitative data) analysed in the current study is presented in the Appendix (see S8 Dataset).

We recorded all interviews, transcribed and analysed them using template analysis [36,37] with MAXQDA 2020 [38]. One researcher conducted the initial coding of the interview data and developed an initial coding template with themes and subthemes regarding the potential of the interventions. The results were discussed and modified within the research team. Afterwards, one researcher applied the final template to the interview data.

We conducted a joint display analysis [39,40], merging quantitative and qualitative data to gain in-depth insights into the feasibility of the programmes. For this purpose, we organised the qualitative and quantitative data based on categories side by side in a table. Qualitative data were used to explain quantitative data. We used Microsoft Excel (version 2019) [41] to create bar charts for visualising quantitative data and Canva Pro [42] to develop illustrations for visualising qualitative data.

### Ethics declarations, consent and data security

Ethics Committee of the Hamburg Chamber of Physicians approved the design and execution of this study (PV6063). All participants received written information about the study. Participation was voluntary, and the consent followed the Declaration of Helsinki and good clinical practice [43]. Participants were informed of the purpose of the study and the nature of the data to be published, and they consented to the inclusion of their anonymised data in the publication. All participating persons provided written informed consent prior to the inclusion in the study. The web conference tool Cisco Webex was hosted on a UKE server to ensure maximum data security and protected communication.

## Results

### Training of the nurses

The nurses received digital training in September 2021 and completed up to two training coaching sessions in October 2021. They felt well-prepared and considered the training informative and sufficient. For more details, see Merging of the qualitative and quantitative data. Both nurses conducted one or two training sessions and successfully applied the SDM principles within these sessions.

### Study population

Between 20 September 2021 and 31 March 2022, we conducted the randomised pilot study. Recruitment of wwMS took place from 20 September 2021 to 28 February 2022. We recruited and randomised 36 wwMS (PtDA = 23, DC = 13). One woman was excluded due to a screening failure (motherhood choice already made), and one dropped out for personal reasons.

**Fig 1:**
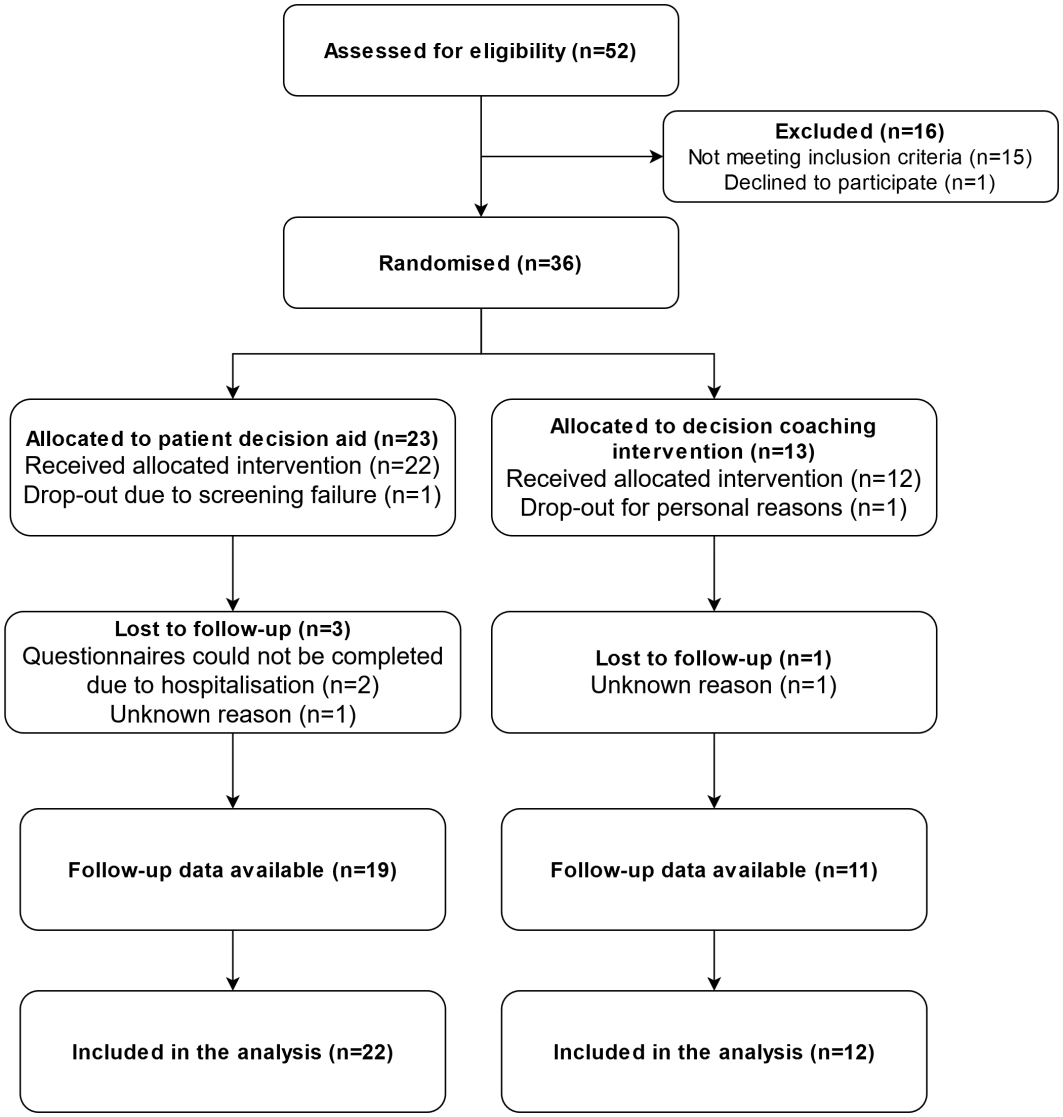
Study flowchart of the randomised pilot study.

We included all wwMS who received the respective intervention in our analysis (PtDA = 22, DC = 12; see Fig 1). In the DC group, six women received decision coaching via phone, and six women received it via web conference. Most women had no children (PtDA 91%; DC 92%), had RRMS (PtDA 73%; DC 85%), and were treated with immunotherapy (PtDA 91%; DC 85%; see Table 1).

**Table 1:** Baseline characteristics.

	PtDA (N = 22)	DC (N = 12)
**Sociodemographic characteristics**		
**Age, mean (SD)**	29.9 (3.7)	30.6 (4.7)
**Education (highest degree) n (%)**		
< 12 school years	2 (9)	3 (25)
≥ 12 school years	20 (91)	9 (75)
**Maternity status n (%)**		
No children	20 (91)	11 (92)
Children	2 (9)	1 (8)
**Diseases specific characteristics**		
**Disease course n (%)**		
CIS	0 (0)	0 (0)
Suspected RRMS/CIS	5 (23)	2 (17)
RRMS	16 (73)	10 (83)
Unclear	1 (5)	0 (0)
**Disease duration n (%)**		
< 1 year	3 (14)	6 (50)
1–5 years	12 (54)	5 (42)
≥ 5 years	7 (32)	1 (8)
**PDDS, Mean (SD)**	1.3 (1.2)	1.2 (1.3)
**Number of relapses in the last 12 months n (%)**		
None	5 (23)	5 (42)
1	9 (41)	4 (33)
2	2 (9)	2 (17)
≥ 3	6 (27)	1 (8)
**Immunotherapy n (%)**		
Yes	20 (91)	10 (83)
No	1 (5)	1 (8)
In the decision-making process	1 (5)	1 (8)
Increase in disability in the last 12 months* n (%)		
No increase	5 (23)	2 (17)
A little bit	12 (55)	7 (58)
A little more	1 (5)	2(17)
A lot	4 (18)	1 (8)

* Self-assessed by an ordinal scaled item (How much has your disability increased due to MS in the last 12 months?).

**PtDA** = patient decision aid; **DC** = decision coaching intervention; **SD** = standard deviation.

**CIS** = clinically isolated syndrome; **RRMS** = relapsing-remitting multiple sclerosis.

**PDDS** = Patient-Determined Disease Steps [26].

### Results regarding decisional conflict and involvement in the decision process

Baseline data were available for 34 wwMS and follow-up data for 31 wwMS (PtDA n = 19; DC n = 12). The PtDA group’s median DCS score was 54 at baseline and 31 at follow-up. In the DC group, the median DCS score was 49 at baseline and 15 at follow-up (see Table 2). An exploratory group comparison (Mann-Whitney U test) indicated lower scores on the informedness subscale of the DCS in the DC group at baseline compared with the PtDA group (p = 0.039).

**Table 2:** Pre- and post-intervention scores regarding decisional conflict (DCS), worries (MPWQ) and knowledge (MCKQ).

	Baseline					Follow-up						
	PtDA (N = 22)		DC (N = 12)		Group comparison	PtDA (N = 22)		DC (N = 12)		Group comparison	Perspective of the coach (only DC)*	
	median (range)	excluded Q	median (range)	excluded Q	P value§	median (range)	excluded Q	median (range)	excluded Q	P value§	median (range)	excluded Q
**DCS^ⱡ^** Total Score	54 (16-100)	0	49 (20-80)	0	0.161	31 (9-67)	3	15 (3-47)	2	0.035	14 (2-44)	0
Uncertainty subscale	63 (8-100)	0	63 (25-92)	0	0.297	42 (8-100)	3	29 (8-58)	2	0.266	21 (0-75)	0
Informed subscale	63 (0-100)	0	50 (17-83)	0	0.029	25 (0-58)	3	13 (0-83)	2	0.040	0 (0-25)	0
Value clarity subscale	46 (17-100)	0	42 (25-75)	0	0.250	25 (0-58)	3	8 (0-42)	2	0.001	4 (0-50)	0
Support subscale	58 (0-100)	0	54 (0-75)	0	0.072	33 (0-58)	3	13 (0-58)	2	0.115	8 (0-25)	0
Effective decision subscale	44 (0-100)	0	38 (19-81)	0	0.260	25 (0-100)	3	13 (0-81)	2	0.050	15 (0-50)	0
**MPWQ** Total Score	45 (26-61)†	1	42 (30-62)‡	0	0.750	40 (23-56)$	3	39 (27-57)#	1	0.343	–	
**MCKQ** Total Score	8 (3-13)	1	8 (5-11)	0	0.986	9 (5-16)	4	11 (6-13)	1	0.307	–	

^§^Mann-Whitney U-test, results should be interpreted with caution due the small sample size; * In the decision coaching intervention, decisional conflict was additionally measured from the perspective of the decision coach; † in n = 1 questionnaire, mean imputation was used for 3 items; ‡ in n = 1 questionnaire, mean imputation was used for 1 item, $ in n = 2 questionnaires, mean imputation was used for 1 item; # in n = 1 questionnaire, mean imputation was used for 2 items.

**PtDA** = patient decision aid; **DC** = decision coaching intervention, **Q** = questionnaires.

**DCS** = Decisional Conflict Scale; Scales 0–100; high scores indicate a higher decisional conflict; DCS scores <25 are associated with implementing decisions; DCS scores >37.5 are associated with decision delay or feeling unsure about implementation [28].

**MPWQ** = motherhood/pregnancy choice and worries questionnaire; 18 items with a 4-point Likert scale; Scale 18–72; higher scores indicate more worries.

**MCKQ** = motherhood choice knowledge questionnaire; 16 knowledge questions; scores give the number of correctly answered questions.

Exploratory group comparison indicated differences in decisional conflict score distributions at follow-up, with lower scores among the wwMS in the DC group than in the PtDA group (p = 0.035; see Table 2). The effect size was moderate (r = 0.4). Group comparisons did not indicate differences between groups regarding the subscales uncertainty and support. For the subscales informed (p = 0.039), value clarity (p = 0.002), and effective decision (p = 0.050), group comparisons indicated lower scores among the wwMS in the DC group at follow-up. The effect size was moderate for the subscales informed and effective decision (r = 0.4) and strong for the subscale value clarity (r = 0.6).

The participant-based MAPPIN’SDM assessment in the DC group (wwMS and coaches; no missing values or questionnaires) showed a good level of perceived involvement in the decision-making process, while the observer-based assessment showed a moderate level (median scores): wwMS 3.5 (SD 0.3), decision coaches 3.5 (SD 0.2), observer perspective 2.2 (SD 0.5).

### Results regarding worries and knowledge

The PtDA group’s median MPWQ score was 45 at baseline and 40 at follow-up. In the DC group, the median MPWQ score was 42 at baseline and 39 at follow-up (see Table 2). In both groups, the median MCKQ score was 8 at baseline. At follow-up, the median MCKQ score was 9 in the PtDA group and 11 in the DC group.

Explorative group comparisons (Mann-Whitney U test) did not indicate no differences between wwMS in the DC and PtDA groups regarding worries and knowledge (see Table 2).

The Wilcoxon signed-rank test for pre-post comparison indicated a significant increase in the MCKQ score in both groups after using the respective programme: DC (n = 12): Z = −2.32, p = 0.020; DST (n = 22): Z = −3.23, p = 0.001. The effect size was large for both pre-post comparisons (DC r = 0.67; DST r = 0.69).

Additional MPWQ items covered coping strategies and attitudes towards motherhood/pregnancy. In both groups, more women reported coping strategies and feeling sufficiently informed to decide after the programmes than beforehand (see Figs S4.3 and S4.4 in S4 File). For attitudes regarding motherhood/pregnancy, the results suggest wwMS in both groups felt informed enough to decide after having received the respective programme. For other MPWQ items relating to attitudes toward motherhood choice, both groups responded similarly before and after the respective programme (see Figs S4.1 and S4.2 in S4 File).

### Process evaluation

**Fig 2:**
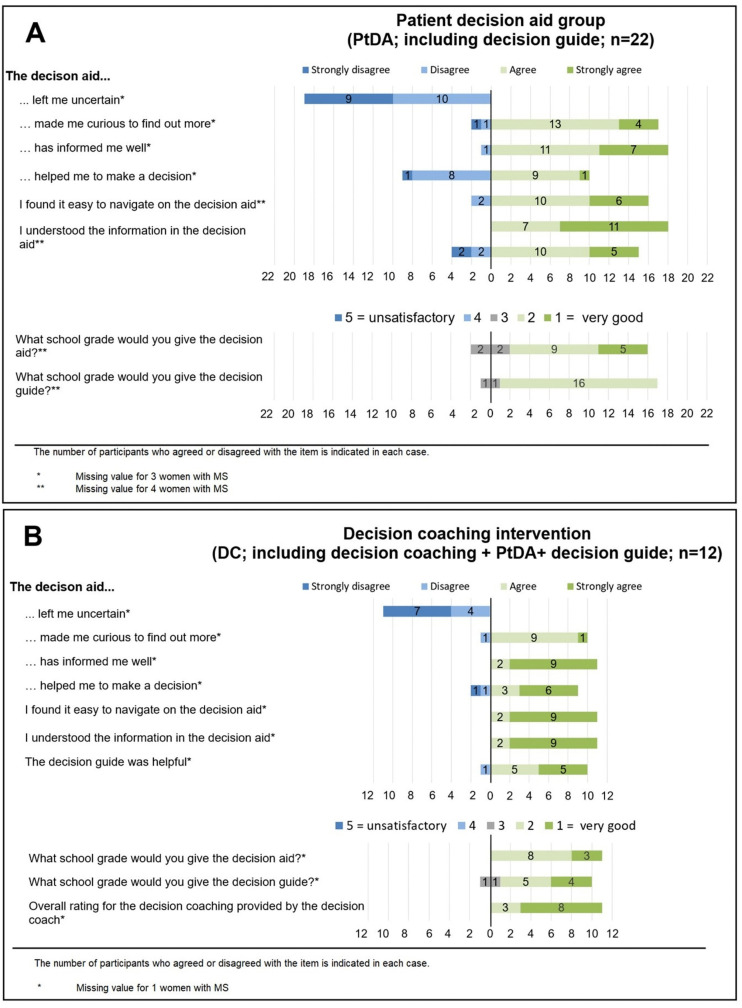
Results of the evaluation questionnaire – women with multiple sclerosis (MS).

WwMS in the PtDA group rated the PtDA with a median score of 2 (1 = very good to 5 = deficient; see Fig 2) and the DG with a median of 2. WwMS in the DC group rated the PtDA with a median score of 2, and the DG^pro^ with a median score of 2. The decision coaching received a median score of 1 (see Fig 2). The decision coaching sessions were perceived as pleasant by wwMS (n = 9), and the duration of the session was considered adequate (n = 8). One woman wished for a longer session.

We asked the wwMS what expectations they had at baseline and how these were met after the programme (e.g., clarification of questions, reduction of uncertainties). WwMS indicated that both programmes met their expectations. This was reported more frequently by wwMS in the DC group (see Figs S5.1 and S5.2 in S5 File).

**Fig 3:**
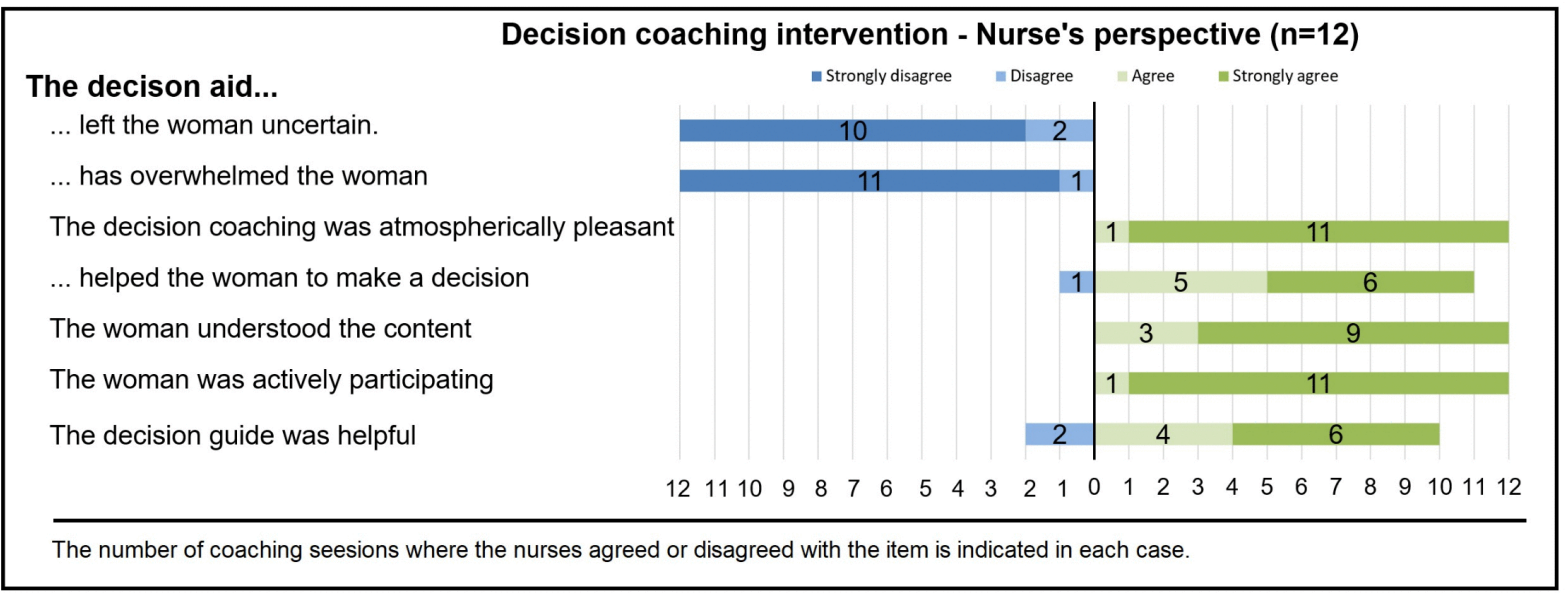
Results of the evaluation questionnaire – Nurse’s perspective.

The decision coaches described the decision coaching as understandable, atmospherically pleasant, and helpful for family planning decisions of wwMS (see Fig 3).

### Interviews with wwMS and MS experts

Between February and March 2022, we interviewed 11 wwMS about the usability of the programmes (PtDA n = 5, DC n = 6). We also interviewed the two nurses about the feasibility of the decision coaching programme. We coded women’s feedback into themes and subthemes regarding expectations and the potential of the support programmes. Nurses’ feedback was coded into themes and subthemes on the potential of the DC and their role as decision coaches (see Figs S6.1 and S6.2 in S6 File).

Women’s feedback was overall positive regarding structure and content and indicated the usability and acceptance of the programmes. In the PtDA group, some information (such as the psychosocial impact of parental MS on children) was perceived negatively, and some wwMS would have liked personal counselling. This was not reported in the DC group, where participants said they felt eased and understood after the coaching session.

In the PtDA group, one woman stated that the programme was interesting but did not help her make a decision. She would have preferred to talk with a health professional. All other women found the respective programme useful for family planning decisions.

In the DC, women reported that decision coaching was the most important component for them since open questions could be answered, worries were reduced, and their decisional tendency was strengthened. The wwMS receiving decision coaching via phone (n = 3) or web conference (n = 3) were similarly satisfied with the medium.

The nurses felt comfortable as decision coaches and well prepared by the training course. No problems with the implementation of the coaching sessions were mentioned. One nurse integrated coaching into her working hours; the other did it as a secondary activity. They noted a positive development in their skills and knowledge. The moderation cards and DG^pro^ were assessed as helpful, and the digital format of the programme was considered acceptable. Feedback from the wwMS was positive and a motivating experience. They found the decision coaching helpful for resolving uncertainties and considered it a valuable add-on to the PtDA. Both nurses reported struggles with certain components of the DG^pro^ (e.g., writing down possibilities, including pros and cons) and found it to be partly too comprehensive. They felt that all the crucial details were communicated and understood by the wwMS during the decision coaching, yet sometimes, the sessions were regarded as too long.

### Merging of the qualitative and quantitative data

**Fig 4:**
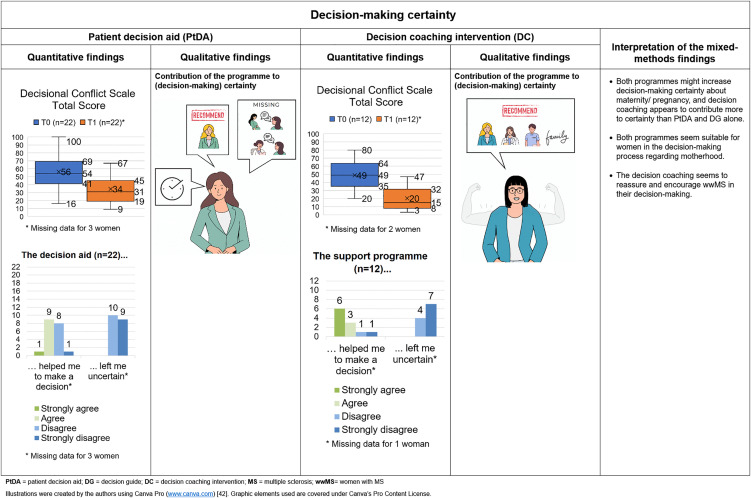
Merging the qualitative and quantitative data from the women with MS – Joint display analysis part 1.

**Fig 5:**
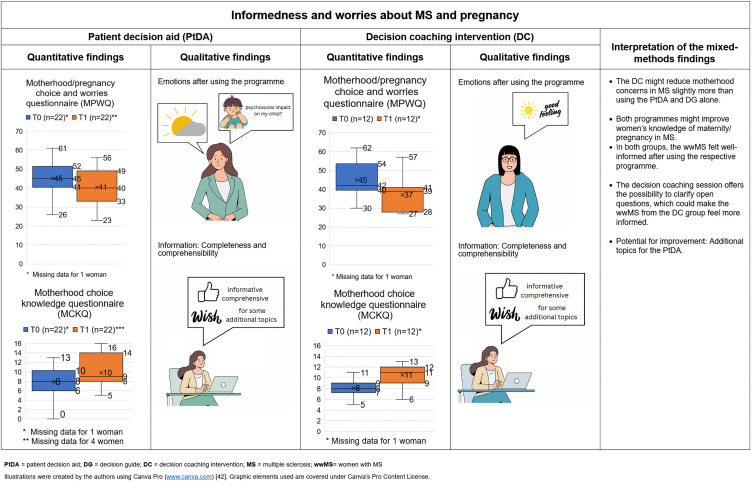
Merging the qualitative and quantitative data from the women with MS – Joint display analysis part 2.

**Fig 6:**
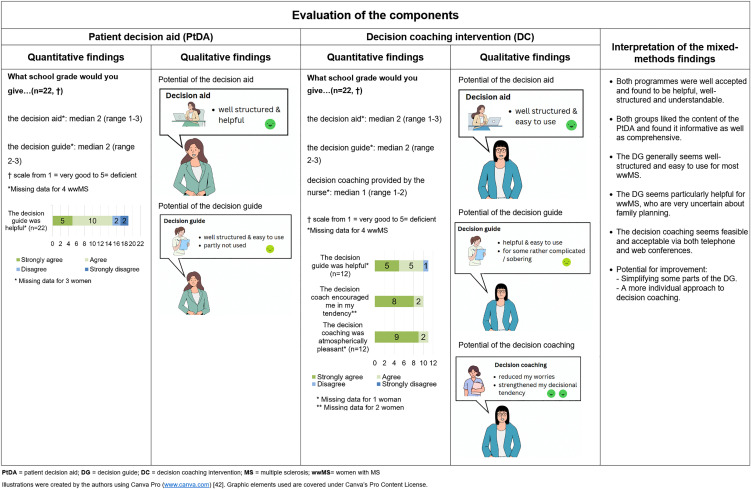
Merging the qualitative and quantitative data from the women with MS – Joint display analysis part 3.

We summarised the qualitative and quantitative data from the wwMS and nurses applying joint display analysis. Overall, the analyses show consistent quantitative and qualitative results regarding acceptance, usability, and appropriateness for the target group (wwMS in family planning). When comparing both data sets, wwMS in the DC group talked about a greater relief of worries in the interviews than we could observe by solely analysing the questionnaires. Qualitative data support the quantitative observations that the DC might contribute more to decision certainty and the reduction of worries about pregnancy in MS (see Figs 4–6).

**Fig 7:**
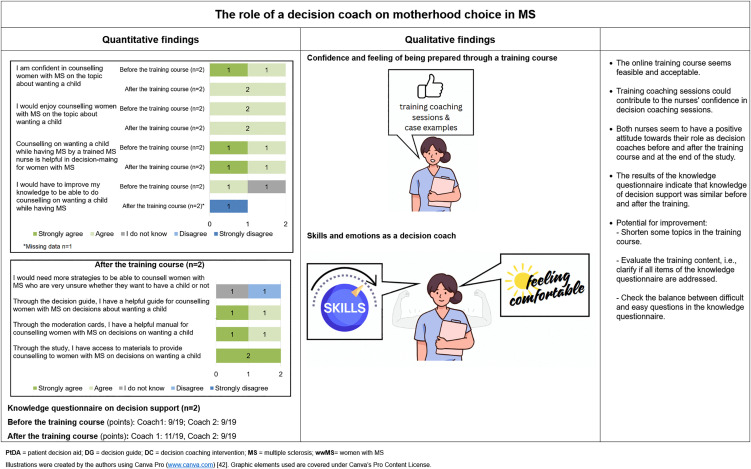
Merging the qualitative and quantitative data from the nurses – Joint display analysis part 1.

**Fig 8:**
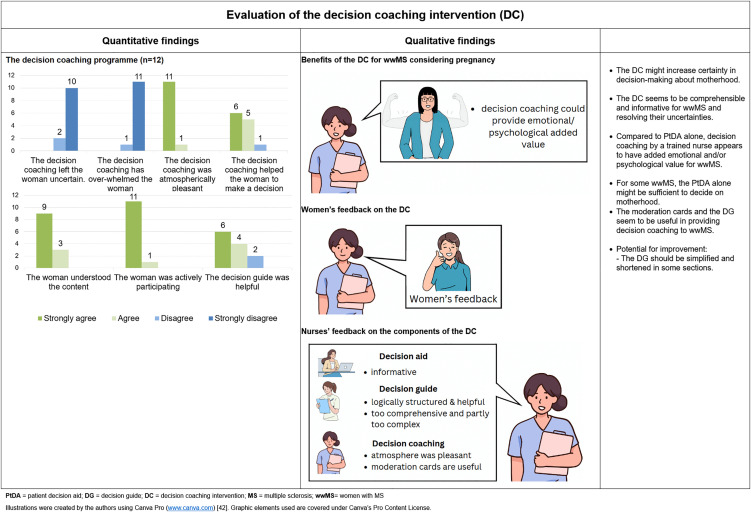
Merging the qualitative and quantitative data from the nurses – Joint display analysis part 2.

Nurses’ qualitative and quantitative data indicate that the online training course, including the training coaching sessions, helps to conduct decision coaching on motherhood choice. When comparing both sets of data, the nurses’ attitudes towards decision coaching on motherhood choice in MS appear to be positive following the training course and the final decision coaching session (see Figs 7-8).

Based on the analysis, we identified areas for improvement. For instance, the PtDA should be optimised for use on mobile devices (e.g., smartphones). Additional topics requested by women could be included in the PtDA group to increase the information value, e.g., regarding miscarriages and costs of an elective caesarean section. A few sections in the DG^pro^ could be shortened and simplified to improve usability for decision coaches and wwMS.

## Discussion

We piloted two decision support programmes on motherhood choice: a PtDA and a decision coaching programme with a PtDA. To our knowledge, our work provides the first decision coaching approach on motherhood choice for wwMS. Our pilot study showed that both programmes are feasible and acceptable for wwMS, having the potential to support wwMS in family planning decisions. Thereby, they might be able to reduce decisional conflict. Especially the decision coaching intervention may have the potential to enhance women’s confidence in their decision-making.

We assessed decision conflict as an exploratory primary endpoint using the DCS. The DCS is widely used in studies involving healthcare decisions, demonstrating good reliability [44]. For pregnancy-related decisions, Whybrow et al. [45] evaluated the effectiveness of ten PtDA in reducing decisional conflict using the DCS. Five of the included randomised controlled trials focused on PtDA for family planning in conjunction with MS [11] or other diseases (epilepsy, n = 30 [46], rheumatoid arthritis, n = 78 [47], depression [48,49]). These five studies demonstrated that a PtDA can significantly decrease decisional conflict and support informed decision-making regarding motherhood compared to usual care, which is consistent with our findings [45].

Due to its personalised approach, decision coaching might be especially beneficial for individuals to feel informed, supported, and confident about their personal values when making health decisions [12]. To explore the potential of our decision coaching intervention in relation to these aspects, we conducted exploratory group comparisons of the DCS subscales. Our group comparison for the informed and value clarity subscales indicated lower scores among the wwMS in the DC group at follow-up than among the wwMS in the PtDA group. This finding indicates that wwMS who received the DC might have experienced fewer struggles with decision-making. In terms of uncertainty and support, our group comparisons did not indicate differences in these subscales. The Cochrane review by Jull et al. used the DCS and its subscales to compare decision coaching with usual care and with a PtDA [50]. Whether decision coaching leads to individuals feeling informed, supported, and confident about their personal values was reported as uncertain, either because it was not measured or because the quality of evidence was very low [50]. Further research is needed to clarify whether decision coaching influences decision conflict, as well as related aspects such as uncertainty, value clarity, and feeling informed, in order to achieve a better understanding of the value of decision coaching for person-centred care. Due to the study design, our results provide only a limited contribution to this research area.

Our explorative group comparison shows no differences in worries at follow-up. Possible explanations could be that worries might take longer to decrease (e.g., the fear of postpartum relapses) than the study period. A pre-post comparison indicated a higher knowledge score for both groups after using the respective programme. Knowledge was also measured in three RCTs (MS [11], rheumatoid arthritis (RA) [47], epilepsy [46]) and one pilot RCT (depression [51]) on patient decision aids about pregnancy for women with different diseases. Knowledge increased in all of the trials’ groups. The increases in the intervention groups, which included a PtDA, were significantly higher. In terms of decision coaching, Jull et al. were unable to draw a definite conclusion regarding knowledge due to the very low evidence certainty [50]. They supposed that decision coaching in combination with a PtDA might improve knowledge compared to usual care alone. Yet, it remains uncertain whether decision coaching together with a PtDA could lead to increased knowledge compared to a PtDA alone [50]. A PtDA could possibly be sufficient to improve knowledge and decision coaching might not enhance this outcome further. However, increasing knowledge is not a primary aim of decision coaching, but rather part of a broader goal. Decision coaching aims to support individuals in making informed, value-congruent decisions. Key aspects are to clarify preferences and communicate them, to identify a person’s decision-making needs, and to help them understand information on benefits, harms, and all possible options [52].

Knowledge alone is not sufficient to make informed health decisions. This assumption is supported by a systematic review by Joseph-Williams et al. [53]. They propose that most PtDA focus on providing information without considering practical aspects of SDM [53]. In our PtDA group, we observed similar feedback. Interviewed wwMS said that the programme was helpful and informative, but a more personal/ emotional aspect was missing. In a qualitative study, Fragkoudi et al. demonstrated the need for emotional support in addition to information accessibility regarding reproductive planning with MS [54]. It appears that for a relevant number of wwMS, written information alone is insufficient, and personal contact seems necessary in the decision-making process. Prunty et al. illustrated the concerns of wwMS facing MS in a qualitative study. Named fears were being unable to care for their child or discontinuation of medication [5]. Thus, decision coaching can be an opportunity to address and overcome negative emotions.

A common topic in the qualitative interviews was the use of immunotherapy during pregnancy and the postpartum period. Several studies report how the worry about the effects of medication on pregnancies or children might impact family planning [54,55] and how these topics can be the cause of worries or anxiety [5,6]. Yet, knowledge about the teratogenic effects of MS treatment is often limited [56]. Until now, only beta-interferons have been approved for use during pregnancy and breastfeeding in Germany with certain limitations. A few other medications can also be used after a risk-benefit assessment [57,58]. However, the potential effects on pregnancy are rarely systematically investigated, and evidence regarding immunotherapies is scarce. In our PtDA, we included data provided by pharmaceutical companies, which collected information on pregnancies that occurred while individuals were taking medication. This information is not systematically published, making it difficult to obtain an up-to-date overview. More studies are needed to determine the effects of MS immunotherapies on fertility, pregnancy, and breastfeeding.

The wwMS in our pilot study reported no serious negative effects, such as anxiety, linked to our interventions. This is in line with current studies reporting that PtDA [9,46,47] and decision coaching [50] have no negative influences. However, we used no validated measurement instruments; instead, we asked women in the interviews how they felt after using the programme.

We obtained detailed information from wwMS on the programme’s acceptance, feasibility, and user-friendliness, which will be useful for further development and evaluation of your programmes. The feedback from the decision coaches was positive. They appreciated their new role, received good feedback from the wwMS, and the implementation of the coaching sessions was unproblematic. In contrast, the DECIMS study by Rahn et al. named several implementation-hindering barriers (e.g., personnel and time resources) [13]. Berger-Höger et al. reported positive experiences with coaching from nurses and physicians in their RCT on nurse-led decision coaching in breast-care centres as facilitators. Yet, the lack of time and insufficient commitment from physicians hindered the implementation [59]. However, our pilot study was conducted in a remote setting with only two nurses working in MS outpatient clinics, which provided good conditions for conducting research projects. In a future RCT, it must be determined how feasible the use of coaches will be from a financial perspective and how the additional task can be included in the nurses’ usual work.

### Limitations and strengths

Our study was not without limitations. Our initial sample size of 64 was not reached, and we could not extend the recruitment period. Recruitment during our pilot study was mainly conducted through written materials (e.g., flyers). Given the emotional nature of the topic of motherhood choice, it may be beneficial to involve the clinical staff of MS outpatient clinics in the recruitment process, thereby encouraging eligible women to participate in a more personal manner. Moreover, many approached women were not eligible to participate in our study because no decision regarding pregnancy/family planning was outstanding, and the women were confident in their decisions. Nonetheless, the recruited sample gives us sufficient information to evaluate the feasibility of the programmes.

Our exploratory group comparisons and pre-post comparisons provide only initial indications of the potential of the two programmes. Due to the small sample size and unequal group distribution, these results should be interpreted with caution. Future studies with larger and more balanced samples are needed to confirm these findings.

The PtDA and the DC programme were online programmes, which might have excluded women preferring face-to-face programmes. On the other hand, our programmes could be accessed regardless of location. Most wwMS had a high level of education. Thus, we could not rule out that wwMS with a lower level of education are less addressed by our programmes. However, we simplified the information in the PtDA to suit the reading level of secondary school students.

Our programmes were developed based on an extensive needs analysis and systematic literature searches following the MRC framework for developing and establishing complex interventions [16]. We adapted the contents of the PtDA based on expert feedback on MS and motherhood, as well as input from pharmaceutical companies. This provided the opportunity to include the most recent information and answer questions that the systematic literature searches could not clarify. We also adapted the programmes by integrating the feedback of wwMS. A similar iterative methodological approach was observed by Meade et al. in their PtDA on motherhood choice in RA, which showed good results [47]. They consulted experts and women with RA via online surveys and questionnaires regarding the content, readability, and structure of the PtDA. They shared a draft of their PtDA for the revisers to comment on [47].

Our programmes could serve as a blueprint for developing further support programmes, particularly those that include personal contact, such as decision coaching on motherhood choice for other medical conditions. A systematic review of health information about pregnancy [60] observed that women with other chronic diseases, e.g., type 1 diabetes mellitus, also have unmet information needs and wish for personalised information.

## Conclusion

The PtDA and the decision coaching programme are feasible and well-accepted programmes for women considering motherhood in MS. Data suggest that the decision coaching programme might have the potential to provide more confidence and clarity in the decision-making process than the PtDA alone. Consequently, it appears beneficial to pursue and refine this approach, testing its effectiveness in a full RCT.

## Supporting information

S1 Fig Overview of the study steps of the project Motherhood choice in multiple sclerosis (MoMS). (TIFF)

S2 Text Detailed information on the development phase and the intervention components. (DOCX)

S3 Text Detailed information on the feasibility/ alpha testing. (DOCX)

S4 File Randomised Pilot/Beta testing – results of the MPWQ. (DOCX)

S5 File Women’s expectations towards the support programme – baseline and follow-up. (DOCX)

S6 File Coding templates and summary tables for the qualitative data – randomised pilot/ beta testing. (DOCX)

S7 Table CONSORT extension to pilot and feasibility trials. (DOCX)

S8 Dataset Anonymised dataset (quantitative data). (XLSX)

S9 Data Study protocol. (PDF)
