# Supplementary figures and images for: Motherhood choice in multiple sclerosis (MoMS) – Pilot trial of web-based decision support
Source: PLoS One. 2026 Jun 12;21(6):e0351108. doi: 10.1371/journal.pone.0351108 (PMC13262864; doi:10.1371/journal.pone.0351108)

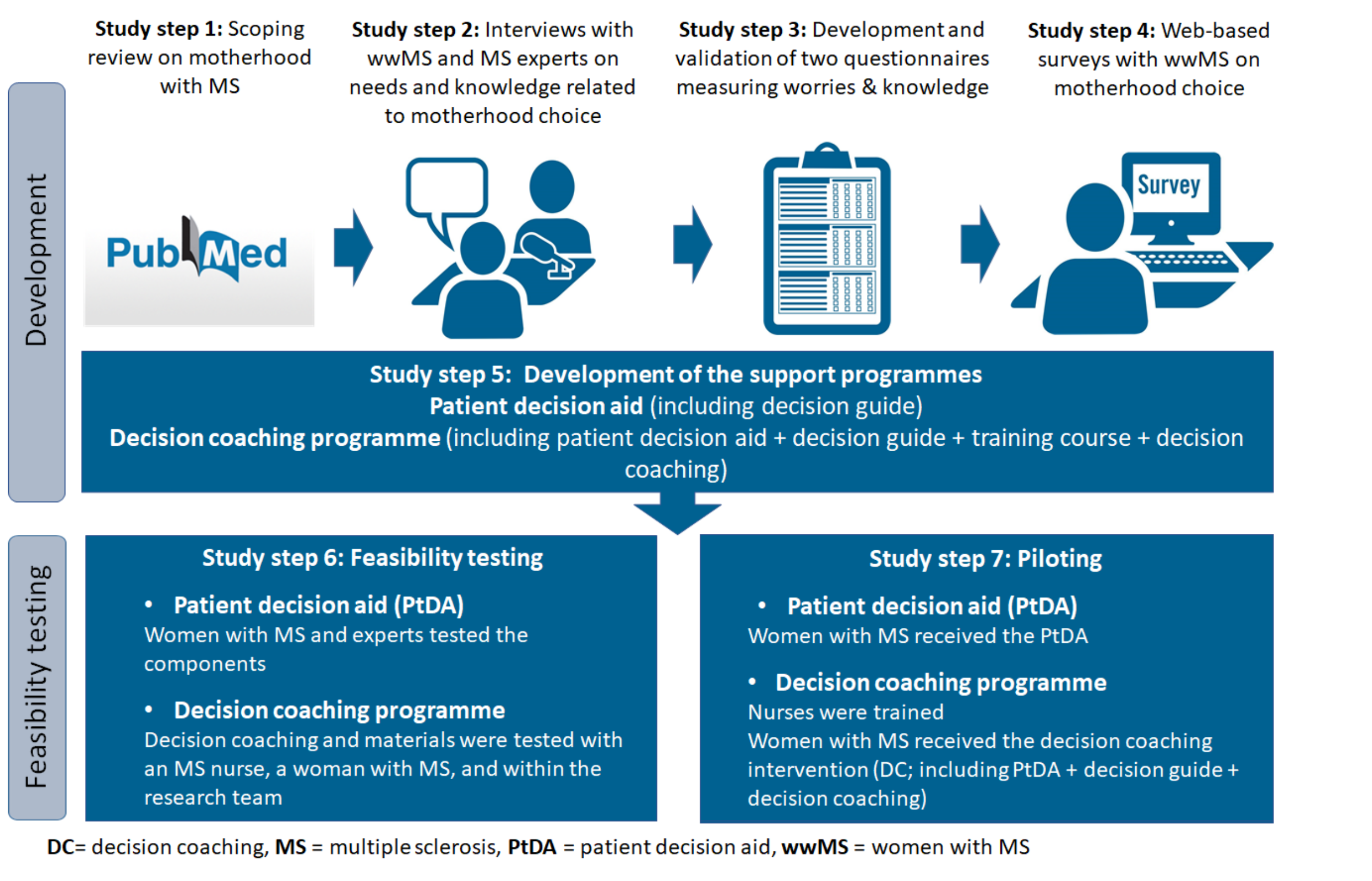

Supplement: S1 Fig — (TIFF) [file pone.0351108.s001.tiff]
