# Supplementary material for: Motherhood choice in multiple sclerosis (MoMS) – Pilot trial of web-based decision support
Source: PLoS One. 2026 Jun 12;21(6):e0351108. doi: 10.1371/journal.pone.0351108 (PMC13262864; doi:10.1371/journal.pone.0351108)
Supplement: S2 Text — (DOCX) [file pone.0351108.s002.docx]

## **S2. Detailed information on the development phase and the intervention components.**

### Theoretical basis

We focused on patient empowerment as an underlying principle [1,2] for the development of our programmes. We applied the Ottawa Decision Support Framework [3] and the Interprofessional Shared Decision-Making Model (IP-SDM) [4] to develop the programmes. The IP-SDM broadens the perspective of SDM by involving family members and decision coaches in the decision-making process. Thus, the IP-SDM supports the implementation of an interprofessional approach to SDM and was used to develop the decision coaching programme [5,6]. We followed the standards of the International Patient Decision Aid Standards (IPDAS) [7] and the principles of EBPI [8].

### Intervention components

We developed the following components for our two support programmes: 1) a web-based PtDA, 2) a workbook (decision guide; DG), 3) a training course for decision coaches, and 4) moderation cards for the nurses. Table A provides an overview of the developed components following the TIDieR checklist [9].

Table S2. Intervention components for the women with multiple sclerosis.

|  | PtDA | Decision guide | Decision coaching session | Training course for decision coaches | Moderations cards |
| --- | --- | --- | --- | --- | --- |
| Why | - To provide information about relevant topics from pre-pregnancy to motherhood so that wwMS can decide according to their values regarding motherhood choices. - To improve the quality of the decisions. | - To structure the decision-making process - To note important information about the decision-making process (e.g. own values and preferences) - To discuss the decision-making process with others (e.g. coach, partner, neurologist) | - To support the decision-making processes and help wwMS achieve informed and value-congruent decisions | - To educate and support nurses in conducting decision coaching sessions | - To support nurses in conducting decision coaching sessions |
| What | - Describes the health condition (MS disease and pregnancy) - Makes the motherhood choice and subsequent decisions explicit - Provides information on pregnancy / motherhood in MS (e.g. fertility, relapse risk, treatment management, progression, pregnancy) | - Helps wwMS clarify their values and preferences - Helps wwMS o structure their decision-making process - The extended version (DG^pro^) contains information on prognostic factors for the MS course and the opportunity to document the individual MS course | - Non-directive decision support - Helps to understand the web-based PtDA - Identification of support needs - Clarification of values and preferences - Encouragement to communicate their preferences and values to another person | - Traget group: Training course for nurses with MS expertise - Discusses the current state of research on motherhood in MS - Practices the use of supporting materials (moderation cards and DG^pro^) - Includes role plays with case examples to practice decision coaching on motherhood choice using the supporting materials | - Include information to follow the six SDM steps |
| Who provides | Online access through a study nurse | Sent by mail through the study nurse | A nurse who has received the training course | Interprofessional research team (AR, CH, and JH) | A nurse who has received the training course |
| How | Web-based | PDF version and print | Telephone or web conference tool Cisco Webex | Online via the web conference tool Cisco Webex | Print version |
| Where | Locally independent | Locally independent | Locally independent | Locally independent | Locally independent |
| When and how much | During the two-week study period, extent varying depending on the information need | Before, during and after the decision coaching session; two-week study period | 1 session (takes up to 1.5 hours) within 2 weeks after inclusion | 12-hour training course and one to two training sessions on the job | During the decision coaching session |
| MS = multiple sclerosis; PtDA = patient decision aid; wwMS = women with multiple sclerosis | | | |  |  |

**Patient decision aid (PtDA)**

We developed a web-based PtDA structured into nine chapters aiming to cover all topics from pre-pregnancy to motherhood and structured it accordingly (see Fig S2). The basis for our PtDA was a decision aid on motherhood choice for wwMS developed and evaluated by Prunty et al. [10].


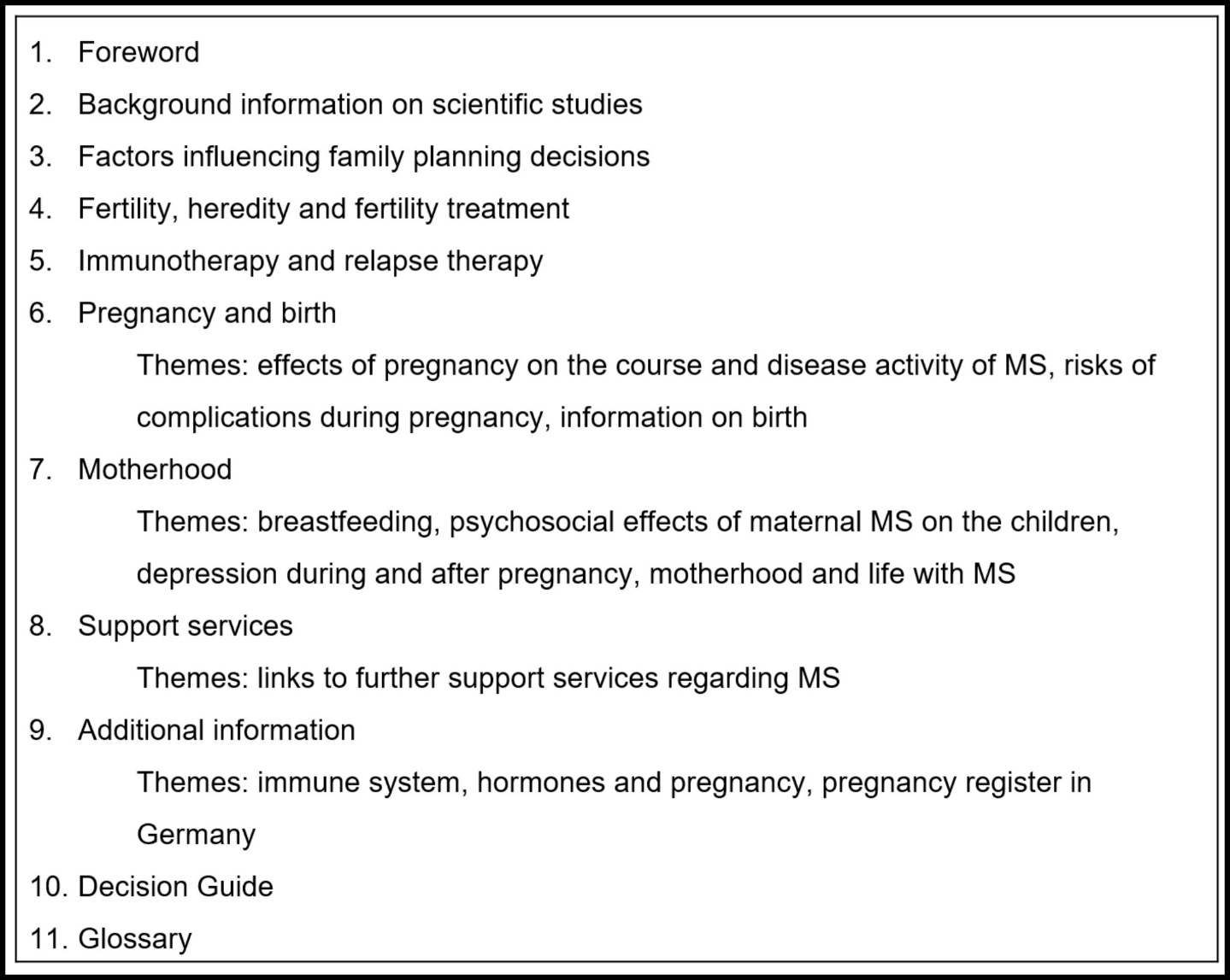


Figure S2. Content overview of the web-based patient decision aid on motherhood choice in multiple sclerosis.

We used the needs assessment results to determine the topics of the PtDA. For content development, we included literature from our initial scoping review and the current German guideline on MS [11].

We contacted pharmaceutical companies to receive safety data on immunotherapies regarding pregnancy and breastfeeding, and feedback on our drafts of the immunotherapy chapters. Studies used in the PtDA were assessed for their quality. For observational studies and systematic reviews, we used checklists from the Critical Appraisals Skills Programme (CASP) [12-14]. Assessment of RCTs was performed with the Cochrane Risk of Bias Tool [15]. We used the text-analysis tool Wortliga [16] to ensure the information in the PtDA matched the understanding of a secondary school student.

We developed a quality assessment system to help wwMS recognise the quality of the used studies (rating very low to high quality). For this system, we focused on explaining scientific studies, evidence-based medicine (EBM) principles, and showing the differing qualities. This quality assessment system aims to help wwMS to evaluate the information for themselves. Since most of the studies from the scoping review were observational studies, we created two videos with Microsoft PowerPoint [17] explaining observational studies.

**Decision guide (DG)**

As a guiding component, we developed a DG, a workbook on motherhood choice based on the Ottawa Personal Decision Guide [18] and a previously developed workbook for immunotherapy decisions in the DECIMS project [19]. The DG complements the PtDA and contains six steps of shared decision-making [20].

For the decision coaching programme, we produced an extended version of the DG (DG^pro^). Compared to the DG, the DG^pro^ contains information on prognostic factors for the MS course and the opportunity to document the individual MS course.

**Decision coaching and training course for nurses**

The decision coaching sessions, conducted by a nurse with MS expertise, cover topics related to motherhood/ pregnancy and MS to facilitate and support informed motherhood choices. Each session lasts up to 1.5 hours and is structured into six steps of SDM [20].

We developed moderation cards to support the decision coaches in performing the decision coaching. The moderation cards include information to follow the six SDM steps. The curriculum for the decision coach training course was guided by established educational theories [21] and the IP-SDM model [22]. A previously developed immunotherapy decision coaching curriculum [19] served as the basis. The curriculum includes a 12-hour training course for nurses with MS expertise and a training on the job. During the training on the job, the nurses performed and recorded one to two coaching sessions with wwMS considering pregnancy. After these training sessions, the nurses received feedback from JH. The training course consists of the current state of research on motherhood in MS and the use of supporting materials (moderation cards and DG^pro^). We developed role plays with case examples to practice decision coaching on motherhood choice using the supporting materials.

**References of the Appendix S2**

1. Castro EM, Van Regenmortel T, Vanhaecht K, et al (2016) Patient empowerment, patient participation and patient-centeredness in hospital care: A concept analysis based on a literature review. Patient Educ Couns 99:1923–1939. https://doi.org/10.1016/j.pec.2016.07.026
2. Bravo P, Edwards A, Barr PJ, et al (2015) Conceptualising patient empowerment: A mixed methods study. BMC Health Serv Res 15:1–14. https://doi.org/10.1186/s12913-015-0907-z
3. Stacey D, Légaré F, Boland L, et al (2020) 20th Anniversary Ottawa Decision Support Framework: Part 3 Overview of Systematic Reviews and Updated Framework. Med Decis Making 40:379–398. https://doi.org/10.1177/0272989X20911870
4. Ottowa Hospital Research Institut (2022) Interprofessional Shared Decision Making (IP-SDM) Model. https://decisionaid.ohri.ca/ip-sdm.html. Accessed 21 Jun 2024
5. Stacey D, Brière N, Robitaille H, et al (2014) A systematic process for creating and appraising clinical vignettes to illustrate interprofessional shared decision making. J Interprof Care 28:453–459. https://doi.org/10.3109/13561820.2014.911157
6. Légaré F, Stacey D, Gagnon S, et al (2011) Validating a conceptual model for an inter‐professional approach to shared decision making: a mixed methods study. J Eval Clin Pract 17:554–564. https://doi.org/10.1111/j.1365-2753.2010.01515.x
7. International Patient Decision Aids Standards Collaboration. (2019) IPDAS 2019: Criteria for Judging the Quality of Patient Decision Aids. https://www.ncbi.nlm.nih.gov/pmc/articles/PMC8474333/. Accessed 11 Jul 2023
8. Lühnen J, Albrecht M, Mühlhauser I, Steckelberg A (2017) Leitlinie evidenzbasierte Gesundheitsinformation. http://www.leitlinie- gesundheitsinformation.de/. Accessed 11 Jul 2023
9. Hoffmann TC, Glasziou PP, Boutron I, et al. Better reporting of interventions: template for intervention description and replication (TIDieR) checklist and guide. *BMJ* 2014; 348: g1687. <https://doi.org/10.1136/bmj.g1687>
10. Prunty MC, Sharpe L, Butow P, Fulcher G (2008) The motherhood choice: A decision aid for women with multiple sclerosis. Patient Educ Couns 71:108–115. <https://doi.org/10.1016/j.pec.2007.10.021>
11. Hemmer B et al Diagnose und Therapie der Multiplen Sklerose, Neuromyelitis-optica-Spektrum- Erkrankungen und MOG-IgG-assoziierten Erkrankungen, S2k-Leitlinie, 2021, in: Deutsche Gesellschaft für Neurologie(Hrsg.), Leitlinien für Diagnostik und Therapie in der Neurologie, Online: <https://www.dgn.org/leitlinie/diagnose-und-therapie-der-multiplen-sklerose-neuromyelitis-optica-spektrum-erkrankungen-und-mog-igg-assoziierten-erkrankungen> Accessed 11 Jul 2023
12. Critical Appraisal Skills Programme. CASP (Systematic Review) [Internet]. 2019. Available from: <https://casp-uk.net/casp-tools-checklists/> Accessed 11 Jul 2023
13. Critical Appraisal Skills Programme. CASP (Case Control Study) [Internet]. 2019; Available from: https://casp-uk.net/casp-tools-checklists/. Accessed 11 Jul 2023
14. Critical Appraisal Skills Programme. CASP (Cohort Study) [Internet]. 2019. Available from: https://casp-uk.net/casp-tools-checklists/. Accessed 11 Jul 2023
15. Higgins JPT, Altman DG, Gøtzsche PC, et al (2011) The Cochrane Collaboration’s tool for assessing risk of bias in randomised trials. BMJ Online 343:1–9. <https://doi.org/10.1136/bmj.d5928>
16. WORTLIGA Tools GmbH. (2021) WORTLIGA (Textanalyse-Software). https://wortliga.de/. Accessed 11 Jul 2023
17. Microsoft Corporation (2022) Microsoft PowerPoint
18. O’Connor, Stacey, Jacobsen (2015) Ottawa Personal Decision Guide©. [https://decisionaid.ohri.ca/docs/das/opdg.pdf.](https://decisionaid.ohri.ca/docs/das/opdg.pdf.%20Accessed%201%20Jul%202023)  Accessed 11 Jul 2023
19. Rahn AC, Köpke S, Backhus I, et al (2018) Nurse-led immunotreatment DEcision Coaching In people with Multiple Sclerosis (DECIMS) – Feasibility testing, pilot randomised controlled trial and mixed methods process evaluation. Int J Nurs Stud 78:. https://doi.org/10.1016/j.ijnurstu.2017.08.011
20. Elwyn G, Edwards A, Wensing M, et al (2001) Shared decision making observed in clinical practice: visual displays of communication sequence and patterns. J Eval Clin Pract 7:211–221. https://doi.org/10.1046/j.1365-2753.2001.00286.x
21. Roth H (1971) Pädagogische Psychologie des Lehrens und Lernens. Hermann Schroedel Verlag KG, Hannover
22. Stiggelbout AM, Pieterse AH, De Haes JCJM (2015) Shared decision making: Concepts, evidence, and practice. Patient Educ Couns 98:1172–1179. <https://doi.org/10.1016/j.pec.2015.06.022>
