# Supplementary material for: Motherhood choice in multiple sclerosis (MoMS) – Pilot trial of web-based decision support
Source: PLoS One. 2026 Jun 12;21(6):e0351108. doi: 10.1371/journal.pone.0351108 (PMC13262864; doi:10.1371/journal.pone.0351108)
Supplement: S3 Text — (DOCX) [file pone.0351108.s003.docx]

## **S3. Detailed information on the feasibility/ alpha testing.**

### Methodological procedure in the feasibility testing

In the feasibility testing we aimed to initially pretest and optimise the components of our support programmes. Thus, we iteratively tested the components with MS experts and wwMS.

**Participants and recruitment**

Health professionals with experience in the care for wwMS considering pregnancy, a patient representative and women with relapsing-remitting MS (RRMS) or clinically isolated syndrome (CIS) between 18 to 45 years were recruited to iteratively assess practicability and acceptance. We included wwMS with ongoing or not yet terminated family planning. The wwMS were recruited via telephone, accessing a study database of the *Institute of Neuroimmunology and Multiple Sclerosis* (INIMS).

The experts were suggested by research team members (AR, CH, KH) or a local MS society of the *German Multiple Sclerosis Society (Deutsche Multiple Sklerose Gesellschaft, DMSG)*. Participants had to give written informed consent.

**Testing the PtDA and decision guide (DG)**

Experts, followed by wwMS, received access to the PtDA and DG for two weeks. Afterwards, they filled out questionnaires focusing on practicability and acceptance. We developed semi-structured interview guides based on the results of these questionnaires. Interviews with experts and wwMS were performed by a researcher (JH) to obtain in-depth information about the usability of the components. We continuously refined the components based on the feedback and suggestions for improvement. We interviewed experts via telephone and wwMS via telephone or web conference.

**Testing the decision coaching programme**

For the usability of the decision coaching programme, we tested the decision coaching and the moderation cards. Research team members alternated between the decision coach and wwMS roles. Subsequently, one researcher tested the decision coaching with an independent MS nurse from the *University Medical Centre Hamburg-Eppendorf* (UKE) and a wwMS. The nurse took the perspective of a woman with MS considering pregnancy. Based on the feedback, we adapted the training course, the decision coaching and the moderation cards.

**Analysis**

For statistical data analysis, we used Microsoft Excel [23]. Demographic data and quantitative data from the questionnaires (evaluation questionnaires) were analysed descriptively. We summarised continuous data using medians, ranges, means and standard deviations. Categorical data were reviewed using frequencies.

We recorded all interviews, transcribed and analysed them with template analysis [24] using MAXQDA 2020 [25]. One researcher conducted the initial coding of the interview data and developed an initial coding template with themes and subthemes regarding the potential of the components. The results were discussed and modified within the research team. Afterwards, one researcher applied the final template to the interview data.

### Results of the feasibility/ alpha testing

Alpha testing took place from April to June 2021 with eight wwMS and five experts. Tables S3.1 and S3.2 show the characteristics of the participants. The coding template regarding feedback from the experts and wwMS can be found in Fig. S3.1. Table S3.3 provides an overview of the themes, subthemes, and illustrative quotes (see pages 7-18).

Table S3.1. Baseline characteristics.

|  | **Women with MS**  (N=8) |
| --- | --- |
| **Sociodemographic characteristics** | |
| **Age, mean (range)** | 34 (26-29) |
| **Education (highest degree)** |  |
| < 12 school years | 2 (25) |
| ≥ 12 school years | 6 (75) |
| **Maternity status (%)** |  |
| No children | 4 (50) |
| Children | 4 (50) |
| **Diseases specific characteristics** | |
| **Disease course** |  |
| RRMS | 7 (87) |
| Unclear | 1 (13) |
| **PDDS, Mean (range)** | 1 (0-3) |
| **Number of relapses in the last 12 months n (%)** | |
| None | 5 () |
| 1 | 2 () |
| 2 | 1 () |
| **Immunotherapy n (%)** | |
| Yes | 5 () |
| No | 3 () |
| **SD** = standard deviation; **RRMS** = relapsing-remitting multiple sclerosis; **PDDS** = Patient-Determined Disease Steps (26) | |

Table S3.2. Baseline characteristics.

|  | **MS-experts**  (N=5) |
| --- | --- |
| **Age, mean (range)*** | 53 (40-61) |
| **Function within the feasibility testing n (%)** | |
| Neurologist | 2 (40) |
| Psychotherapist | 1 (20) |
| Patient representative | 1 (20) |
| Member of a self-help organisation | 1 (20) |
| **Self-assessment of level of MS expertise n (%)*** | |
| Excellent | 0 (0) |
| High | 3 (75) |
| Moderate | 1 (25) |
| Limited | 0 (0) |
| **Self-assessment of level of expertise in MS and maternity/pregnancy n (%)*** | |
| Excellent | 1 (25) |
| High | 2 (50) |
| Moderate | 1(25) |
| Limeted | 0 (0) |
| ***** missing data for one expert; **MS =** multiple sclerosis | |

**Experts’ feedback and revisions**

Two experts did not complete the evaluation questionnaire, but one of them provided answers during the interview. Overall, experts gave the PtDA a mean score of 2 (1 = very good – 6 = unsatisfactory; range 1-3; missing data for one expert) and the DG a mean score of 2.5 (range 2-3, missing data for one expert).

The experts found the PtDA to be objective, logically structured and overall comprehensible. They liked the evidence-based approach and quality assessment of the literature. However, all experts stated that the PtDA was partly too comprehensive and suggested summaries. They found the PtDA trustworthy but had differing views on to what extent the PtDA is helpful in its current form, i.e. without counselling or being complex and lengthy. Two experts found the PtDA helpful for wwMS. One expressed concern that the PtDA might unsettle women already quite determined to have a child. Another expert did not see any additional value to existing information services. These experts suggested using the PtDA and DG within a counselling situation.

Some deemed the DG helpful, but it was partly too abstract. An expert stated that the emotional and psychological points of view were missing in the DG and DA.

Revisions: We shortened the PtDA and added missing information, e.g. about certain immunotherapies, psychosocial aspects and postpartum depression. In the initial scoping review, the psychosocial effects of maternal MS on the children were not included. Hence, a systematic review of psychosocial effects was conducted [27]. Graphic illustrations were added to the DA. We added instructions to simplify the DG.

**Feedback from wwMS and revisions**

We conducted two online focus groups with seven wwMS (3 to 4 per group) and one individual interview via telephone. WwMS gave the PtDA a mean score of 2.4 (1 = very good – 6 = unsatisfactory; range 2-5) and the DG a mean score of 2 (range 1-3, missing data for three wwMS). We prepared a printable summary of the PtDA based on the feedback from the evaluation questionnaire, which we showed during the interviews. The women thought that all important topics were covered in the PtDA and liked the summary. However, they found the PtDA partly too comprehensive and wished for shorter chapters and additional graphics. The quality assessment was pointed out to be informative and important. The PtDA was deemed comprehensible, objectively written and easily navigable.

Generally, the PtDA and DG were mostly considered supportive for a decision on motherhood, with varying extents. The participants wished for testimonials and regular actualisation of the webpage.

Revisions: We added more graphics to illustrate the contents of the PtDA in the form of summarising boxes at the beginning of each chapter (see Fig. S3.2) and shortened the chapters. Additional information was listed separately for interested readers to reduce comprehensiveness. We referred to another project for testimonials (Patient Experiences of Multiple Sclerosis, PexMS [28]) and included the chapter “Motherhood and living with MS”.

**Feedback on the decision coaching sessions and revisions**

Within the research team, the moderation cards were considered useful but too extensive. There was a lack of support for difficult situations (e.g., a woman not ready to make a decision). The nurse found the decision coaching helpful but remarked that it might be too detailed for some wwMS.

Revisions: We shortened the moderation cards and parts of the decision coaching and added guidance for difficult coaching situations.

*
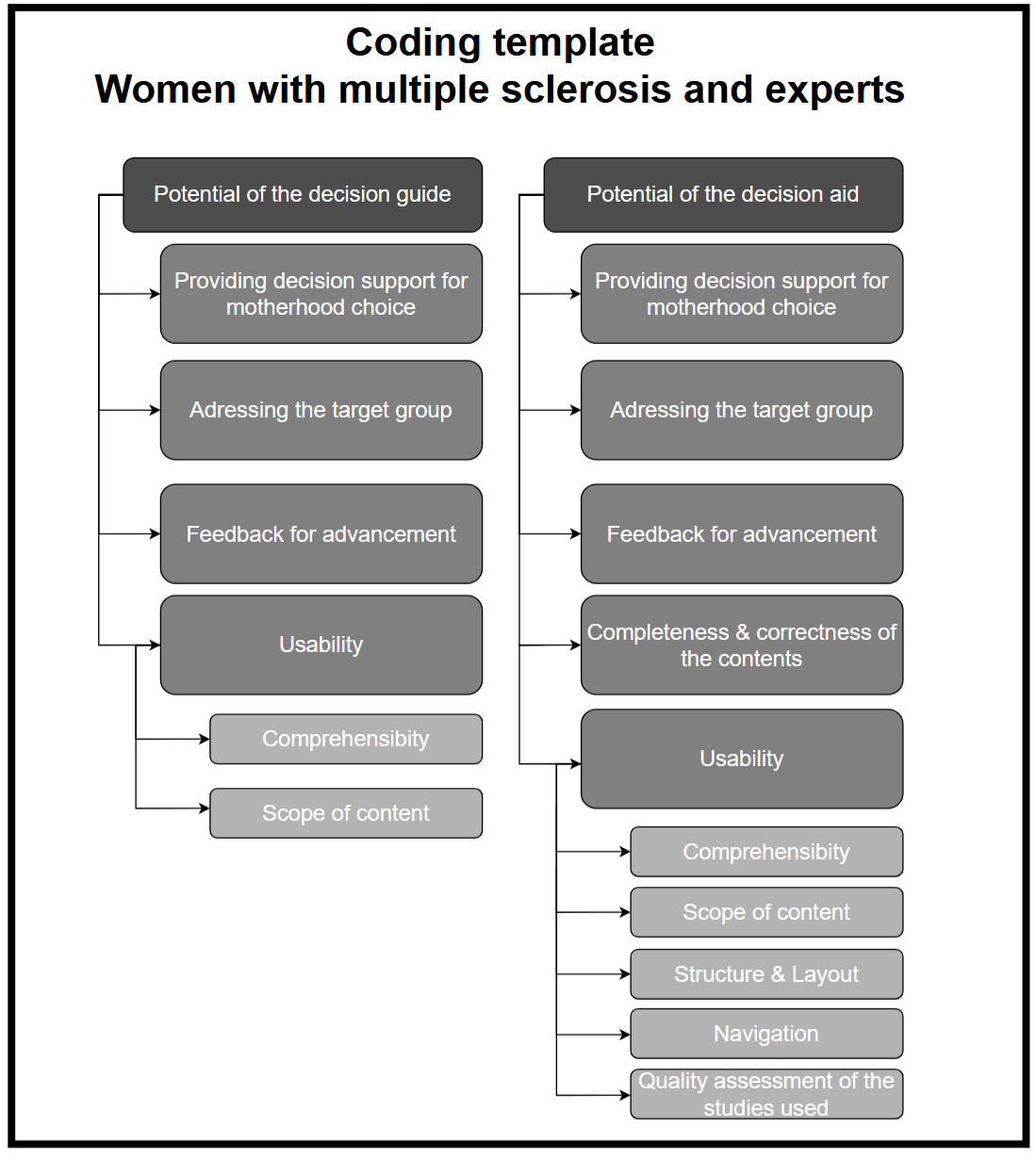
*

**Fig S3.1. Coding template for the evaluations of the interviews with the women with multiple sclerosis and experts - alpha testing.**

## **Table S3.2. Summary table of the themes with illustrative quotes – alpha testing.**

| **Overarching Theme** | **Theme** | **Illustrative quotes** |
| --- | --- | --- |
| **Potential of the decision guide** | Providing decision support for motherhood choice | “Ich habe sie mir weniger aktiv vorgestellt, so von Patientinnenseite, also das finde ich aber einen guten Punkt eigentlich, dass man da selber... dass es da diese Schriftfelder, wo man das aufschreiben muss, weil wie gesagt, ich glaube, das hilft, um sich das selber auch nochmal bewusst zu machen, dass man das dann nochmal verschriftlichen muss. Genau. Ich habe es mir passiver vorgestellt, ich habe gedacht, das sind dann einzelne Punkte, die man dann irgendwie so... also genau das ist jetzt dieser Decision Guide, glaube ich, auf den ich im Speziellen anspreche, genau.“ *Personal interview, wwMS*  WwMS 1: „Es ist sehr gut gemacht, und für Leute, die das wirklich auch schriftlich brauchen, oder vielleicht hätte es mir ausgedruckt und wäre es einfach durchgegangen, vielleicht hätte ich mir andere Notizen draufgemacht. Ich weiß jetzt nicht ob ich wirklich alles so ausgefüllt hätte, wie es da jetzt steht, aber, es ist auf jeden Fall eine schöne Grundlage, um dann irgendwas damit anzufangen.“ … WwMS 3: „Ja, dass du das mit zum Arzt noch nehmen kannst. Wenn man sagt, man hat irgendwie ein Kinderwunsch, also einfach nur als Idee, man kann es ja auch einfach mit einpacken und sagen: "Okay, dann nehme ich das mit und bespreche es beim Neurologen nochmal, meine Fragen und so weiter und sofort." Und dann hat man es nochmal so ein bisschen gebündelt. Also, ich finde das ganz gut gemacht.“ WwMS 1: „Ja, das ist eine gute Idee. Grad für solche Momente, wo man zur Konfrontation mit jemandem geht, wo man vielleicht sich vorher mal hinsetzt und vielleicht andere, weitere persönliche Fragen noch mit aufschreibt.“ WwMS 2: Ich habe auch nichts hinzuzufügen." *Focus group 2*  “Genau. Also ich... also für mich war es jetzt so, dass noch nicht so ganz klar wurde, also wenn ich mich jetzt als Patient vorstelle, ob sich nicht eben der eine oder andere vielleicht ein bisschen direkt damit überfordert fühlt. Ich glaube, dass es wichtig ist, darauf so hinzuleiten, dass man sagt, sprechen Sie erst mal mit jemandem und dann können sie sich hier vertiefen und dann kann man eben entweder erst mal alleine letztlich oder gemeinsam mit dem Arzt vielleicht noch diese Möglichkeiten, also da gibt es dann diese Möglichkeiten 1, 2, 3 und so weiter ausfüllen. Und das erschließt sich jetzt noch nicht so ganz intuitiv, glaube ich, für manche Patienten, was die dann... also wie dann der Weg genau ist sozusagen.  Also das hat sich mir noch nicht so ganz erschlossen und dann denke ich mir, vielleicht erschließt es sich den Patienten auch noch nicht so ganz, ehrlich gesagt, ja. Also ist es denn so... also bei dem Decision Guide habe ich ja dann „wo stehe ich“, „warum muss ich diese Entscheidung treffen“, das ist etwas, was der Patient dann alleine für sich zu hause alles mal niederschreibt und bringt das ganze Paket dann wieder mit zum Arzt, oder wie ist das? Das habe ich noch nicht so ganz verstanden, wie da genau der Weg gedacht ist." *Expert interview 4*  “Ich glaube tatsächlich, dass es einfach… Also wissen Sie, es gibt ganz viele… Ich bin ja ein großer Freund von… ich mache Listen und mache Priorisierungen und bin ja auch ein großer Freund von so Decision-Hilfen. Ich glaube aber, dass es eines der Themen ist, wo so viel an Angst und so viel aber auch auf der anderen Seite an Hoffnung dran ist, dass das nichts ist, was ich sozusagen per Fragebogen-Beurteilung oder Selbsteinschätzung wirklich gut geklärt kriege. Ich glaube wirklich, dass wenn jemand da im Entscheidungsprozess ist, einen Ansprechpartner braucht, mit dem er sozusagen diese Aspekte wirklich auch direkt erörtern kann.“ *Expert interview 1* |
|  | Addressing the target group | WwMS 1: „Und, ja, also das müsste man sich ja ausdrucken und dann wirklich auch handschriftlich vielleicht selbst oder mit dem Partner mal machen. Ich weiß nicht ob ich das tatsächlich, ob ich mich handschriftlich hingesetzt hätte, und dass jetzt wirklich ausgefüllt hätte, vor, was weiß ich, fünf Jahren. Aber selbst, wenn man das nicht so macht, wie es angelegt ist, dass man das dann alles schriftlich ausfüllt, sind es ja einfach auch Anregungen für Fragen, Fragenstellungen und. Insofern finde ich das auf jeden Fall erstmal sinnvoll.“ WwMS 3: „Sehe ich ganz genauso.“ WwMS 2: „Ja. Das macht ja auch jeder anders. Also, ich glaube, ich könnte es mir gut vorstellen, dass es auch viele, also ich wäre jetzt auch nicht jemand, die das ausdruckt und Mindmaps, sozusagen, oder Brainstorming macht, auch schriftlich, aber es gibt bestimmt viele, denen das dann hilft, das ist dann eigentlich eine ganz gute Idee auf jeden Fall.“ WwMS 1: „Also, ich kenne auch Leute, die sich so hinsetzen und wirklich solche Listen erstellen, und das schriftlich brauchen. Insofern ist es ja ein Angebot für die unterschiedlichen Typen, und ich kann mir schon sehr gut vorstellen, dass das wirklich einige so auch machen, wie das angedacht ist, mit ausdrucken und ausfüllen." *Focus group 2*  „… es gibt schon so ein Drittel, mindestens ein Drittel, die haben sehr klare Vorstellungen, also die wissen genau, sie möchten jetzt das und das planen, wo ich sagen würde, die brauchen das gar nicht. Die brauchen dann im Prinzip eigentlich nur die klar strukturierte Beratung, okay, was muss ich jetzt beachten mit meiner Therapie und mit der MS geht das, muss ich vorher was absetzen oder nicht. Also das ist so, gut, das ist mindestens ein Drittel der Frauen, die haben ganz klare Vorstellungen. Dann gibt es die, da würde ich sagen, da ist eine große Unsicherheit da, immer auch noch in Richtung, darf man überhaupt mit MS Kinder kriegen, wo man einfach auch ein bisschen aufklären muss, in der Richtung, weil es da ja immer noch all' die Meinungen gibt, dass das gar nicht möglich ist. Und dann gibt es eben die, wo es tatsächlich so ist, wo ich sagen würde, da würde wahrscheinlich das mit rein spielen jetzt, dass man... dass welche sich das aufschreiben, mit Möglichkeit 1, 2 und 3 und so weiter und dann vor allem auch gemeinsam mit dem Partner vielleicht dann überhaupt erst mal eine Entscheidung entwickeln.  Also das brauchen, weil sie eben alleine damit zu unsicher sind und nicht damit zurecht kommen und da ist, denke ich, wahrscheinlich so ein Coaching dann mit dabei sicher nicht schlecht, weil das ja so ein Prozess ist, den ein Paar dann auch erst mal ein Stückweit eben gemeinsam gehen muss, wo dann vielleicht sagt der eine ja, der andere sagt nein oder beide sind sehr unsicher, wo dann glaube ich so was niederschreiben und sich genau nochmal alle Pro's und Contra's anschauen und mit Leitung dabei, schon hilfreich sein kann." *Expert interview 4* |
|  | Feedback for advancement | Interviewer: „Fehlt Ihnen da auch der emotionale Aspekt?“ Expert: „Ja, irgendwie ja. Also wenn ich jetzt an meine eigenen Schwangerschaften denke – ich habe drei Kinder – da haben wir zum Beispiel beim ersten Kind, mein Mann und ich, wir haben … da gab es damals so ein bisschen, glaube ich, auch feministisch angehaucht und so, Berichte von Frauen, die Natürlich geboren haben, wie sie das gemacht haben. Diese Geburtshilfe wie heute, mit Familienzimmer und Badewanne, gab es noch nicht so richtig. Aber es war schon sozusagen in der Luft. Aber man fand das nicht so ohne weiteres irgendwo. Man musste dann genau gucken, in welches Krankenhaus man geht. Oder Hausgeburt, oder so. Und Berichte von Frauen oder Paaren oder Familien, wie die ganze Geburt gelaufen ist, das haben wir sehr sehr gerne gelesen. Also ich vor allen Dingen. Mein Mann ein bisschen weniger. Aber ich vor allem, im Sinne von: sich identifizieren, sich einstellen, sich einstimmen. Und in dem Bereich, in dem ich ja auch sehr viel arbeite, mit Verlusten, wenn Familienangehörige sterben, und wie es den Anderen geht, da lesen die das auch. Wie geht es anderen, die ein Kind verloren haben. Wie geht es anderen, wo die Mutter gestorben ist, oder der Vater gestorben ist, oder so. Und ich weiß nicht, ob das für MS-Kranke, die sich mit Kinderwunsch tragen, ob die nicht auch so was suchen. Aber man könnte einfach nur darauf hinweisen. Dieser Decision-Guide macht das eben NICHT. Also Decision-Guide, ich mag auch das Englische nicht. Ich finde, das suggeriert sozusagen: wenn sie den machen, dann haben sie ihre Decision. Und das ist unterkomplex für die Frage 'Will ich eine Familie gründen'." *Expert interview 2*  Expert: „Also ich glaube, da müssen Sie unbedingt noch hinterlegen, was damit gemeint ist.“ Interviewer: „Also mehr Hilfe, damit man sich das auch nutzbar machen kann.“ Expert: „Ja. Ja. Also immer Beispiele. Also da haben Sie ja geschrieben: zum Beispiel, warum muss ich diese Entscheidung treffen? Da müsste ja dann auch wieder ein Beispiel hin. Ja, mein Partner möchte es; oder ich bin schon 35; oder, ja, so was. Also das würde ich … hier geht das ja noch. Aber da unten wird es dann wirklich sehr anspruchsvoll.“ *Expert interview 5* |
|  | Usability | **Comprehensibility**  WwMS 2: „Es ist genug, was man ausfüllen kann, für die Menschen, die auch ganz viel schriftlich halt gerne machen. Das finde ich ganz gut. Und auch das Mindmap finde ich ganz praktisch. Also ich finde das im Großen, ja, also ich finde es gut. Ich habe mir das nur schon vor längerer Zeit angeschaut, ich habe es nur vergessen. Ich musste es mir jetzt gerade nochmal angucken.“ WwMS 1: „Also, es ist mir jetzt nichts präsent im Kopf, dass ich sagen müsste, das gefiel mir nicht, sondern, sondern. Nein, es ist-“ WwMS 2: „-Verständlich, gut." *Focus group 2*  Interviewer: „Sie haben sich ja auch den Entscheidungsleitfaden, den sogenannten [unverständlich 00:12:58] Guide angeschaut und mit einer Zwei bewertet. Gibt es etwas, was wir verbessern könnten an dem [unverständlich 00:13:07] Guide?“  Expert: „Ich finde ihn ein bisschen ja, ein bisschen abstrakt also es, ich weiß jetzt nicht wie ich das beschreiben soll aber es wäre jetzt für mich zum Beispiel, ich hätte den nicht genutzt, muss ich jetzt sagen. Er ist bestimmt gut aber deshalb auch die Zwei, weil ich denke, dass es bestimmt Leute gibt, die den toll finden, weil auch wichtige Fragen einfach geklärt werden aber mir ist ein Touch zu abstrakt und ein bisschen ein Beispiel, wie es gemeint ist, glaube ich, wird dem ein oder anderen helfen also ich erlebe halt immer wieder, dass ich wirklich so begriffsstutzige Menschen am Telefon habe, die dann aber halt einfach begriffsstutzig sind, weil sie überfordert sind mit ihrer Entscheidung, die sie treffen wollen und ich kann mir nicht vorstellen, dass sie damit zurechtkämen. Allerdings, wie gesagt, die Entscheidung [unverständlich 00:14:02] an sich umfangreich, dass ich für mich da jetzt so alles herausgezogen hätte und dass den gar nicht also, dass ich das sowieso gar nicht gebraucht hätte.“ *Expert interview 3*  „Na, der ist schon sehr anspruchsvoll. Also sage ich mal, der Beginn 'vor welcher Entscheidung stehe ich' … also eine Frau, die keinen Kinderwunsch hat, …“ *Expert interview 5*  **Scope of content**  WwMS 4: „Ich fand, der Decision Guide hatte Aspekte drin gehabt, ich weiß noch nicht, wenn man eh schon einen Kinderwunsch hat, ob man den dann so komplett ausfüllen würde, das alles machen würde.“ WwMS 3: „Mir wäre der wahrscheinlich auch zu viel Arbeit." *Focus group 1* |
| **Potential of the decision aid** | Providing decision support for motherhood choice | "Ich finde die Entscheidungshilfe toll, weil es gibt, glaube ich, viele MSlerinnen, die sich, die keine Kinder bekommen möchten, auf Grund dessen, weil sie Angst haben, wegen Ihrer MS, und es kann sehr viel Angst nehmen, diese Entscheidungshilfen, und deswegen finde ich das so gut, wie es aufgebaut ist. Ich selber hätte sie glaube ich nicht gebraucht, weil ich halt gute Leute um mich rum hatte, die direkt auch zu mir gesagt haben: "Mensch, das und das kannst du machen", "und das ist toll", und, die einem auch die Angst genommen haben, und die Entscheidungshilfe, wenn man halt wirklich was hat und keine Beratung bekommt, in dem Fall vom Neurologen, was halt eigentlich ziemlich wichtig ist, bei einer MS, kann die Entscheidungshilfe sehr viel helfen. Von daher, das finde ich ganz gut." *Focus group 2, wwMS 2*  "Genau, und ich hätte mir auf jeden Fall die Entscheidungshilfe gewünscht, für mich. Weil, mir ging es auch so, dass ich Google befragt habe, und im Grunde nur persönliche Erfahrungen von anderen Müttern, oder eventuellen Müttern gelesen habe, und mich das eher verwirrt hat, oder verunsichert hat. Aber wenn ich relativ schnell auf die Seite gekommen wäre, dann wäre das mir einfacher gefallen, zu sagen: "Ja." Wobei, für mich es so klar war, ich möchte ein Kind. Aber wenn man unsicher ist, hilft das einem noch viel mehr." *Focus group 2, wwMS 3*  Interviewer: „Inwieweit kann Ihrer Meinung nach eine Entscheidungshilfe für Frauen mit MS bei der Entscheidung ein Kind zu bekommen, unterstützen? „ Expert: Ich denke schon, dass es zum großen Teil der Entscheidung ausmacht, was also die Information ist einfach für eine Entscheidung zu treffen, sehr wichtig und ich finde jetzt in der Entscheidungshilfe wird schon alles, viele also so ziemlich alles gefragt also mir blieb jetzt keine Frage irgendwie für mich offen und das denke ich schon, dass das viel ausmacht, wenn man sich, gerade wenn unsicher ist und die, wenn man jetzt wirklich so hin- und hergerissen ist, ist eine gute Information, die einem halt ja auch zeigt, dass es jetzt nicht unbedingt, natürlich ist es jetzt schon ein Risiko eine Schwangerschaft einzugehen aber im Großen und Ganzen jetzt keine Katastrophe die hereinbrechen muss, weil das ist ja auch [unverständlich 00:04:27] Info hat und was man so liest ja halt auch extrem unterschiedlich und die Ansichten sind ja auch von vielen, es gibt ja auch ganz viele Frauenärzte, die dann völlig entsetzt sind, wenn man als kranke Frau vielleicht dann noch ein Behinderungsgrad hat, so ging es mir zumindest, da wird man ja fast schon also völliges Entsetzen, wenn man dann so sagt, man hätte gerne in Kind. Also und das fand ich jetzt bei dieser Entscheidungshilfe, das macht, denke ich, schon zuversichtlich, dass man auch alle Fragen, die man sich vielleicht selbst noch nicht gestellt hat, abarbeiten kann oder zumindest mal durchlesen kann und sich herausziehen kann, was man möchte." *Expert interview 3*  Interviewer: „Inwieweit kann Ihrer Meinung nach eine Entscheidungshilfe, Frauen mit MS bei der Entscheidung ein Kind zu bekommen, unterstützen?“ Expert:“ Also ich glaube, dass Ihr Material erstmal sehr gut ist für Frauen, die mit so einem Material umgehen können. Man braucht eine hohe Lesekompetenz. Auch das eine Video, was ich angeschaut habe, ist über 10 Minuten. Das ist lang. Und wenn ich dann bei Kohortenstudie angekommen bin, weiß ich vielleicht gar nicht mehr, was die Beobachtungsstudie war, weil ich ja gar nicht als Forscherin … Das sind Worte, die ich noch nie gehört habe. Das repräsentiert sich dann nicht gleich. Das ist für uns natürlich ganz was anderes. Aber wenn das jetzt Akademikerinnen sind, dann können sie das auch schnell erfassen. Es braucht aber menschliche Unterstützung. Am besten wäre, jemand kennt das gut.“ *Expert interview 2* |
|  | Addressing the target group | WwMS 3: „Ich würde sagen, vor allem neu diagnostizierte Patienten, weil man gerade da am Anfang sehr verunsichert ist mit allem, noch wenig Informationen hat. Ich glaube, da würde es am meisten helfen.“ WwMS 4: „Dem würde ich mich auch anschließen.“ WwMS 2: „Ja, und auch … Entschuldigung. Hatte ich vergessen, aber alles gut.“  WwMS 1: „Oder nicht nur neu-diagnostizierte, sondern auch welche, bei denen sich der Kinderwunsch erst später herausstellt.“ *Focus group 1*  „Ja, das glaube ich vor allem... genau, wie Sie sagen, wie ich auch schon in den vorherigen Fragen gesagt habe, ich könnte mir gut vorstellen, dass vielleicht Frauen, die einen schwereren Verlauf der MS haben, sich davon vielleicht... oder denen das weiter hilft. Dann auch Frauen, die vielleicht nicht das Glück haben, so ein gutes soziales Umfeld zu haben, mit einem sicheren Job, einer festen Partnerschaft, Betreuungsmöglichkeiten vielleicht auch die leicht verfügbar sind. Genau, um das einfach mal so für sich zu priorisieren und einzusortieren, ja." *Personal interview, wwMS* |
|  | Feedback for advancement | „Vielleicht die Studienanzahl ein bisschen komprimieren und auf die wichtigsten Studien beschränken und dass man vielleicht eine kurze Zusammenfassung am Ende eines Kapitels hat. Und, was wir auch schon angesprochen hatten, vielleicht ein paar mehr Grafiken." *Focus group 1, wwMS 4*  „… selbstverständlich, aber eine Aktualisierung, wenn dann neue Therapien auf den Markt kommen, aber das versteht sich glaube ich von selber. Aber ansonsten..." *Personal interview, wwMS*  „Ja. Also, bei manchen Kapiteln waren ja Zusammenfassungen. "Zusammengefasst kann man sagen." Irgendwie sowas. Und da habe ich manchmal gedacht, gerade bei Kapiteln, die jetzt für mich nicht so, wo ich nicht das Gefühl hatte, ich will jetzt alles lesen, ich habe jetzt gelesen aber, ob man das nicht irgendwie Layout technisch irgendwie andersfarbig hervorhebt, so dass man, auch wenn man durchblättert, auch Kapitel liest, wo man vielleicht jetzt nicht von Vornerein sagt, das lese ich mir alles durch, vielleicht genau diese Punkte mit Zusammenfassung trotzdem sofort sieht. Das ist ja jetzt bisher nicht besonders herausgestellt.“ *Focus group 2, wwMS 1*  WwMS 3: „Also solche Erfahrungsberichte, oder so kurze Statements wäre ich glaube ich wirklich cool darüber nachzudenken, ob man sowas mit rein nimmt, von Müttern mit MS.“ WwMS 2: „Finde ich auch eine coole Idee, ja. Finde ich auch gut.“ WwMS 3: „Also sonst war ja fast, also, war ja alles drin. Ist immer noch." *Focus group 2*  „Ich glaube, das was ich jetzt eigentlich schon so angesprochen habe, dass man vielleicht das noch ein bisschen modularer dann gestaltet. Wenn Sie eh' sagen, dass da auch vielleicht nochmal Unterstützung zur Seite gestellt wird, dass es eben nicht... dass man nicht so... nicht hinein fällt, in die Entscheidungshilfe ohne da dann wieder so vor einem riesen Berg an Informationen halt steht, die man ja dann auch für sich auch erst mal wieder bündeln muss. Deshalb glaube ich, ist so ein Coaching vielleicht gar nicht schlecht, ja. Mehr fällt mir jetzt auch nicht ein, nein." *Expert interview 4*  „Ja, habe ich eben schon gesagt. Vaterschaft adressieren. Auch der Vater als Co-Parent – nicht so reinschreiben, aber für uns jetzt hier – einer MS-Kranken. Also dieser familienorientierte Blick. Niemand ist allein krank. Es sind immer andere irgendwie mit betroffen.“ *Expert interview 2* |
|  | Completeness & correctness of the contents | WwMS 2: „Also ich finde schon, dass da sehr viel gestillt wird, viel Erwartung auch, und die Fragen auch gestillt werden, und dass man da auch, also das wirklich eine Entscheidungshilfe ist, dass man auch weiß: Okay, möchte ich das jetzt wirklich noch? Oder möchte ich es nicht? … Das ist halt das, solche, dafür ist die Entscheidungshilfe halt super. Also, dass man halt eine ganz normale Schwangerschaft haben kann. Bei keiner Schwangerschaft weiß man, ob das alles gut geht. Da muss man ja auch immer so sagen. Also dafür muss man keine MS haben, also das ist so, das finde ich ganz toll, was man da für Infos halt bekommt.“ WwMS 3: „Sehe ich ganz genauso. Also ich habe jetzt keine Frage, die ich hatte, bevor ich Schwanger, die jetzt nicht beantwortet wurde.“ WwMS 2: „Genau, ich auch nicht. WwMS 1: „Ja, also ich schließe mich dem an." *Focus group 2*  „Also für mich sind da keine Punkte offen geblieben. Ich finde das schon sehr, sehr umfangreich, also dass Sie da an jegliche Aspekte irgendwie gedacht haben. Nein, für mich bleiben da keine Fragen offen." *Personal interview, wwMS*  Interviewer: „Welche Informationen würden Sie sich für die Entscheidungshilfe zusätzlich wünschen?“ WwMS 4: „Ich bekomme ja zum Beispiel Tysabri und das war auch schon damals ein Thema. Da steht drin, man soll es absetzen in der Schwangerschaft, wenn es möglich ist. Und ja, dann wird beschrieben, dass es zu schweren Rebound-Schüben kommen kann, aber dann fehlt irgendwie die Information, ob ein anderes MS-Medikament sinnvoller wäre, um dem Kinderwunsch nachzugehen. Sollte man erst einmal auf ein anderes Medikament wechseln? Welches ist für den Kinderwunsch gut geeignet, welches Medikament? Und auch was konkret zu tun ist, wenn doch einmal ein Schub in der Schwangerschaft auftreten sollte.“ WwMS 3: „Dem würde ich mich anschließen. Das wären noch gute Informationen gewesen. Ich glaube auch, da hat man immer die meiste Verunsicherung." *Focus group 1*  Interviewer: „Hat die Entscheidungshilfe die Gebiete zum Thema Kinderwunsch und MS abgedeckt, die Ihrer Meinung nach für Frauen mit MS wichtig sind?“ Expert: „Ja, im Prinzip ja. Bis auf das Psychologische, was ich ja immer wieder sage." *Expert interview 1*  Interviewer: „Finden Sie, dass die Entscheidungshilfe für Frauen mit MS hilfreich ist, um sich über verschiedenen Themen zum Kinderwunsch bei MS zu informieren?“ Expert: „Ja, denke ich schon, weil es halt wirklich alle Fragen also ist jetzt auch was Erblichkeit angeht wird ja angesprochen, also es wird halt, ich finde es ist einfach umfangreich. Es wird an vieles, vieles gedacht und ich erlebe das immer wieder, dass mal Frauen anrufen und zwei, drei Fragen haben und ich denke okay gut, sie sind informiert und nach einer Woche später fragen sie mich dann noch mal und so zieht sich das dann über Wochen hin also von daher, das finde ich schon, dass eigentlich alles, wenn man sich jetzt wirklich unschlüssig ist oder man sich sicher ist, aber einfach noch mal durchgehen will, ob man wirklich an alles gedacht hat, so ging es mir zumindest, da finde ich schon, weil wirklich alles also mir hat jetzt nichts gefehlt und ich denke schon, dass wenn man eine Entscheidung treffen möchte, es wirklich eine Hilfe ist." *Expert interview 3* |
|  | Usability | **Comprehensibility**  Interviewer: „Wie würden Sie die Verständlichkeit der Informationen bewerten?“ WwMS: „Finde ich sehr verständlich." *Personal interview*  „Und dementsprechend war die Entscheidungshilfe für dieses Kind nicht weiter relevant, vielleicht für das zweite eher, fand das aber sehr informativ und vom Wissenschaftlichen her auch neutral gut zusammengefasst." *Focus group 1, wwMS 3*  WwMS 3: „Ja, genau. Bilder sind immer gut und machen das Ganze einfacher als so viel Text.“ WwMS 2: „Mir hätte das auch bei den Studien geholfen, die besser zu verstehen, als wenn man im Text nachlesen muss, ob die Wahrscheinlichkeit oder das Risiko jetzt hoch oder herunter geht et cetera, dass man auch mehr Grafiken in die Richtung hätte." *Focus group 1*  „Man braucht eine hohe Lesekompetenz. Auch das eine Video, was ich angeschaut habe, ist über 10 Minuten. Das ist lang. Und wenn ich dann bei Kohortenstudie angekommen bin, weiß ich vielleicht gar nicht mehr, was die Beobachtungsstudie war, weil ich ja gar nicht als Forscherin … Das sind Worte, die ich noch nie gehört habe. Das repräsentiert sich dann nicht gleich. Das ist für uns natürlich ganz was anderes. Aber wenn das jetzt Akademikerinnen sind, dann können sie das auch schnell erfassen. Es braucht aber menschliche Unterstützung. Am besten wäre, jemand kennt das gut. Aber das haben Sie ja auch drin, glaube ich, ne?" *Expert interview 2*  **Scope of content**  Interviewer: „Wie empfinden Sie diesen Umfang an Informationen in der Entscheidungshilfe?“ WwMS 3: „Ausreichend auf jeden Fall.“ WwMS 1: „Aber tatsächlich nicht erschlagend. Also, ich, es gibt ja Seiten, da fühlt man sich schon völlig überfordert, wenn man das so öffnet. Dieses Gefühlt hatte ich nicht, also ich fand das gut zu bewältigen.“ WwMS 3: „Nicht zu viel nicht zu wenig.“ WwMS 1: „Ja.“ WwMS 2: „Ja, so ein schönes Mittelding, einfach so zusammengestellt wurde." *Focus group 2*  „Also was ich sagen muss, ich finde die Entscheidungshilfe an sich gut aufgebaut, ich finde der Umfang der Informationen, der ist wirklich beeindruckend, also ich glaube, da kann man ganz, ganz viel zu finden. Also ich fand es als jetzt Mutter eines noch sehr jungen Kindes, ich muss dazu sagen, ich habe das auch nachts durchgearbeitet, weil man tagsüber gar nicht die Zeit dafür findet, fand ich es teilweise etwas sehr umfangreich. Also das war auch der Punkt, den ich angebracht habe, in dem Fragebogen, also was mir am Deutlichsten aufgefallen ist, dass ich mir wünschen würde, dass es ein bisschen aufgelockert wird, der Text irgendwie.“ *Personal interview, wwMS*  „Ja, ich finde es eben – was ich vorhin schon mal sagte – sehr detailliert, und mir fehlten halt die Zusammenfassungen. Also für jemand, der eben medizinisch überhaupt keine Vorbildung hat, der liest, und liest, und liest, und ihm fehlt aber sozusagen die Main-Message zu den einzelnen Punkten. Also im Detail ist alles da. Klar. Das ist überhaupt keine Frage. Aber so der Duktus des Lesens, finde ich, der ist etwas schwierig." *Expert interview 5*  “Es ist eher zu lang, aus meiner Sicht. Man muss scrollen. Es ist wie ein ganz langer Wikipedia-Artikel. Und das Vorwort sollte eher ein bisschen wie so ein Überblick sein. Also im wahrsten Sinne des Wortes wie so ein Editorial einleiten.” *Expert interview 2*  **Structure & Layout**  Interviewer: „Wie empfinden Sie das Verhältnis von Grafiken zu Text?“ WwMS 3: „Also es ist mehr Text natürlich. Wenn Sie das jetzt meinen.“ WwMS 1: „Aber das fand ich nicht schlimm. Mir ist auch nicht bewusst, was man jetzt noch verbildlichen sollte. Also, ich, das ist in Ordnung, dass mehr Text als Grafik da ist.“ WwMS 2: „Sehe ich auch so. Verhältnis ist ausgeglichen" *Focus group 2*  "Und bei anderen Unterkapiteln, da fand ich es teilweise, das hat einen etwas erschlagen mit dieser Textmenge und das ist ja auch teilweise dann auch ein relativ kleiner Text und wenig Punkte sind hervorgehoben, so visuell, so dass man auch nicht so gut durch den Text so hüpfen kann, irgendwie von Schlagwort zu Schlagwort. Man muss sich dann tatsächlich das alles... oder man könnte sich das alles durchlesen, genau, fand ich einfach ein bisschen... das wäre super, wenn man das noch ein bisschen weiter auflockern könnte. Ich glaube, das würde es noch ansprechender gestalten und ja. Nun muss ich natürlich auch dazu sagen, ich nehme an der Studie teil, weil ich Sie gerne in Ihrer Tätigkeit unterstützen will und nicht primär weil ich sozusagen diese Informationen suche, das ist ist dann eher ein sekundärer Effekt." *Personal interview, wwMS*  “Da kommen wir im Grunde wieder auf diese vielen Zahlen und Statistiken zu sprechen. Die haben das Ganze so ein bisschen schwierig gemacht. Man arbeitet sich nicht so durch einen Text und legt sich dann die Zahlen nebeneinander und guckt, wie es ausschaut. Da wäre wirklich eine Grafik viel besser gewesen, wo man schnell einmal drüber guckt und die Informationen bekommt, die man benötigt. Es war zu viel Text." *Focus group 1, wwMS 3*  Interviewer: „Hmm (bejahend). Fanden Sie die Anordnung und die Reihenfolge der Kapitel in der Entscheidungshilfe sinnvoll?“ Expert: „Absolut. Ja. Ja, die ist gut. Das ist tatsächlich gut gemacht." *Expert interview 1*  Interviewer: „Fanden Sie, dass die Texte mit dem Layout klar präsentiert werden?“ Expert: „Ja. Eben auch sehr nüchtern. Es ist sozusagen vom Inhalt und dem Layout stimmig. Aber es ist eine nüchterne, eben wissenschaftliche Darstellung.“ *Expert interview 2*  **Navigation**  WwMS 2: „Ich fand das nicht schwierig, sich zurechtzufinden. Es war eigentlich alles selbsterklärend und mehr oder weniger intuitiv. Dadurch, dass auch das Inhaltsverzeichnis im Grunde jederzeit präsent war und man jederzeit darauf Zugriff hatte, war das vom Zurechtfinden kein Thema, finde ich.“ WwMS 4: „Ich hatte auch keine Probleme, mich zurechtzufinden. Durch das Menü konnte man sicher ganz gut die einzelnen Themen sehen und anklicken. Man konnte immer auf ‚zurück‘ und ‚weiter‘ klicken.“ WwMS 3: „Dem würde ich mich anschließen." *Focus group 1*  “Das finde ich gut gemacht. Also das mit den Reitern oben auf der Website, mit dem Inhalt und dem Inhaltsverzeichnis und so, genau, kann man sich gut zurecht finden, meiner Meinung nach." *Personal interview, wwMS*  "Ich weiß nicht, ob es dafür ausgelegt ist, dass man es auf dem Smartphone oder Tablet nutzt, aber da finde ich es noch nicht richtig dargestellt. Die Verknüpfungen waren bei mir zu mindestens nicht [unverständlich 00:12:04] immer nur weiter." *Focus group 1, wwMS 1*  Interviewer: „Haben Sie sich in der Entscheidungshilfe leicht zurechtgefunden?“ Expert: „Ja. Ich bin nun auch da … Ja. Man kommt gut immer wieder zurück, und man hat immer diese Unterkapitel. Das fand ich gut.“ *Expert interview 2*  Interviewer: „Fanden Sie sich in der Entscheidungshilfe leicht zurecht?“ Expert: „Ja, also jetzt in dem Gesamt-Tool, nicht nur im Decision-Guide ne… Ja, ja, auf jeden Fall.“ *Expert interview 1*  **Quality assessment of the studies used**  WwMS 2: „Ich würde ich anschließen, aber ich fand das mit den Studien eigentlich ziemlich gut, bei den meisten. Manche waren mir auch zu viel, dann bin ich auch drüber geflogen einmal, aber ich finde es eigentlich auch ganz nett, zu wissen, woher die Informationen eigentlich kam. Weil man ganz oft irgendetwas hört im Sinne von die Schubrate ist geringer während der Schwangerschaft et cetera, aber man weiß gar nicht so richtig, woher das kam, wie gut die Studien sind, wie die durchgeführt worden, welche Aussagekraft das hat. Und das fand ich wiederum eigentlich an den Stellen ziemlich gut. An anderen Stellen, muss ich auch zugeben, dachte ich dann eher: „Das ist mir jetzt auch gerade egal eigentlich.“ Wie zum Beispiel, wie lange ich im Krankenhaus bin. Da würde ich zustimmen, das fand ich nicht so wichtig. Und mit USA stimmt natürlich auch, dass das dann gar nicht so eine Aussagekraft hat für uns in Deutschland oder in Europa an sich. Aber ansonsten fand ich es eigentlich gut, zu wissen, woher die Aussagen kommen. Vor allem, weil die meisten Studien eigentlich keine wirklich gute Aussagekraft hatten." *Focus group 1, wwMS 2*  „Interessant. Aber ich muss auch dazusagen, ich komme aus Studien, von der Arbeit her auch. Für mich sind solche Informationen immer interessant und wichtig und das war jetzt auch so verständlich erklärt, aber ich bin da ein bisschen voreingenommen." *Focus group 2, wwMS 2*  "Ich habe mir da auch eine Notiz gemacht, also was ich zum Beispiel sehr, sehr gut finde ist dieses Bücher [unverständlich 00:10:45] irgendwie zu der Qualität der Studie, das fand ich super, weil man da auf einem Blick direkt weiß, okay, wo befinde ich mich hier sozusagen, das fand ich super." *Personal interview, wwMS*  „Also was ich gut fand, ist wirklich diese Evidenzbasierung. Wie Sie sozusagen das erklären, was heißt evidenzbasiert. Auch mit diesen Büchern. Das ist zum Beispiel eine Visualisierung, ne. Von den Studien. Verstehen Sie, was ich meine? Diese kleinen Bücherstapel, die Sie da …“ Expert interview 2  „Ja, ich denke, dass es schon hilfreich ist, aber es sind halt... ich glaube, das sind immer so Sachen, die jetzt insgesamt nicht so die größte Relevanz haben. Das ist ja sogar eher was, was dann auch einen Arzt interessiert. Ich glaube, das ist so... also es ist für jemanden, für eine Patientin mit einer sehr wissenschaftlichen Herangehensweise, die es natürlich gibt, es gibt die hoch aufgeklärten und sehr strukturiert denkenden und wie auch immer, wie gesagt, sehr wissenschaftlichen herangehenden Patientinnen, für die das glaube ich wichtig ist.  Aber für viele, viele Patientinnen glaube ich, ist das jetzt nicht das Wesentliche, dass sie gucken, oh wie ist denn da die Qualität, sondern das Wesentliche ist das Inhaltliche so, wo sie dann eben in ihrer Unterscheidung unterstützt werden.“ *Expert interview 4* |


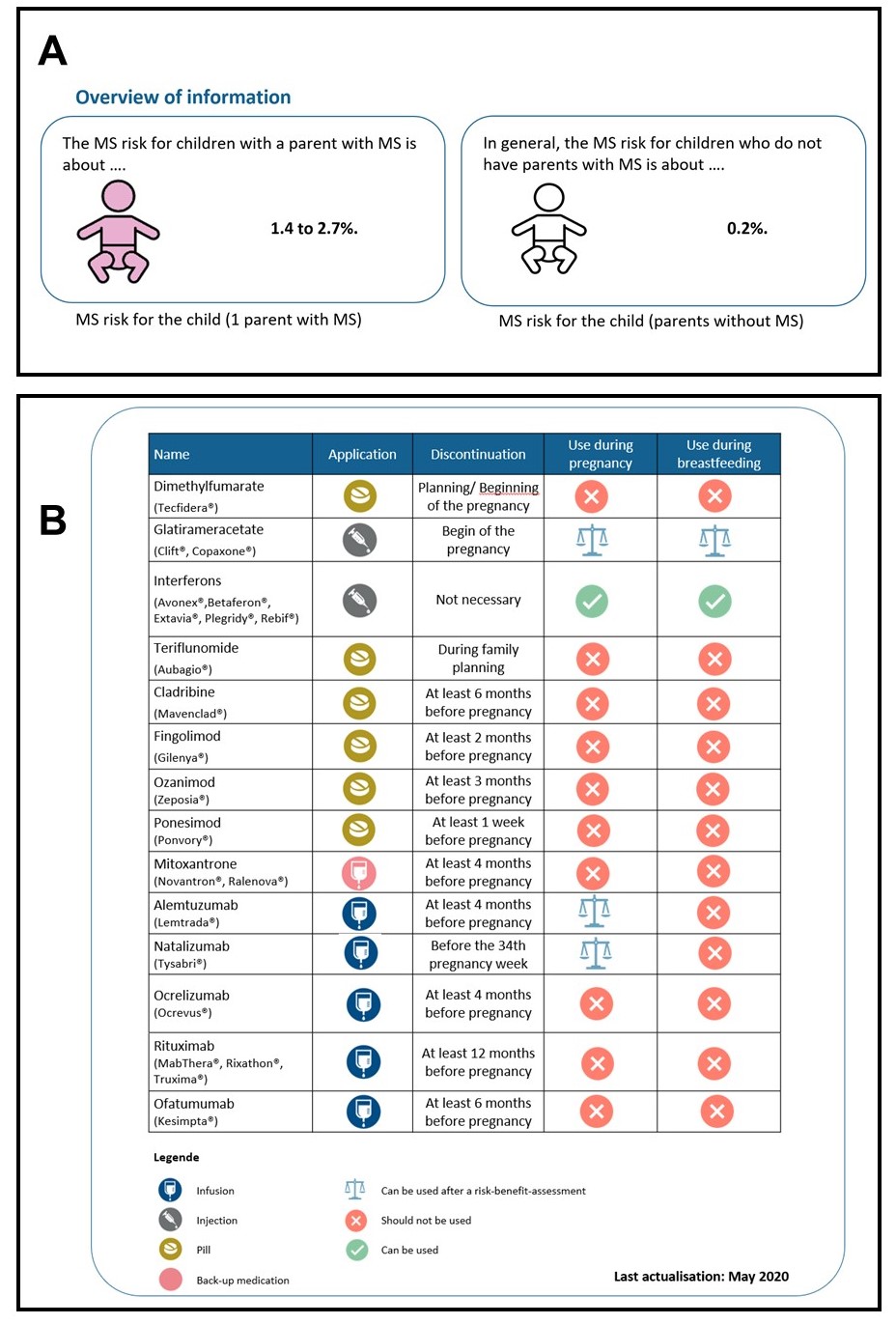


**Fig S3.2. Exemplary infoboxes of the patient decision aid: Heredity and immunotherapies – translated to English**.

**References**

1. Microsoft Excel [Internet]. 2022 [cited 2022 June 23]. Available from: <https://www.microsoft.com/de-de/microsoft-365/excel?market=d>
2. King N Template Analysis. <https://research.hud.ac.uk/research-subjects/human-health/template-analysis/> Accessed 5 Oct 2022
3. VERBI Software. MAXQDA 2020 [Internet]. VERBI Software 2019, MAXQDA 2020, computer program, VERBI Software, Berlin; 2020 [cited 2023 July 11]. Available from: <https://www.maxqda.com>
4. Learmonth YC, Motl RW, Sandroff BM, et al (2013) Validation of patient determined disease steps (PDDS) scale scores in persons with multiple sclerosis. BMC Neurol 13:. https://doi.org/10.1186/1471-2377-13-37
5. Haker M, Peper J, Haagen M, et al (2022) The Psychosocial Impact of Parental Multiple Sclerosis on Children and Adolescents: A Systematic Review. Int J MS Care. https://doi.org/10.7224/1537-2073.2021-075
6. Sippel A, Scheiderbauer J, Eklund D, et al (2022) Development and evaluation of a website with patients experiences of multiple sclerosis: a mixed methods study. BMC Neurol 22:1–14. <https://doi.org/10.1186/s12883-022-02663-9>
