# Supplementary material for: Motherhood choice in multiple sclerosis (MoMS) – Pilot trial of web-based decision support
Source: PLoS One. 2026 Jun 12;21(6):e0351108. doi: 10.1371/journal.pone.0351108 (PMC13262864; doi:10.1371/journal.pone.0351108)
Supplement: S4 File — (DOCX) [file pone.0351108.s004.docx]

## **S4 File. Randomised Pilot/Beta testing – results of the MPWQ.**


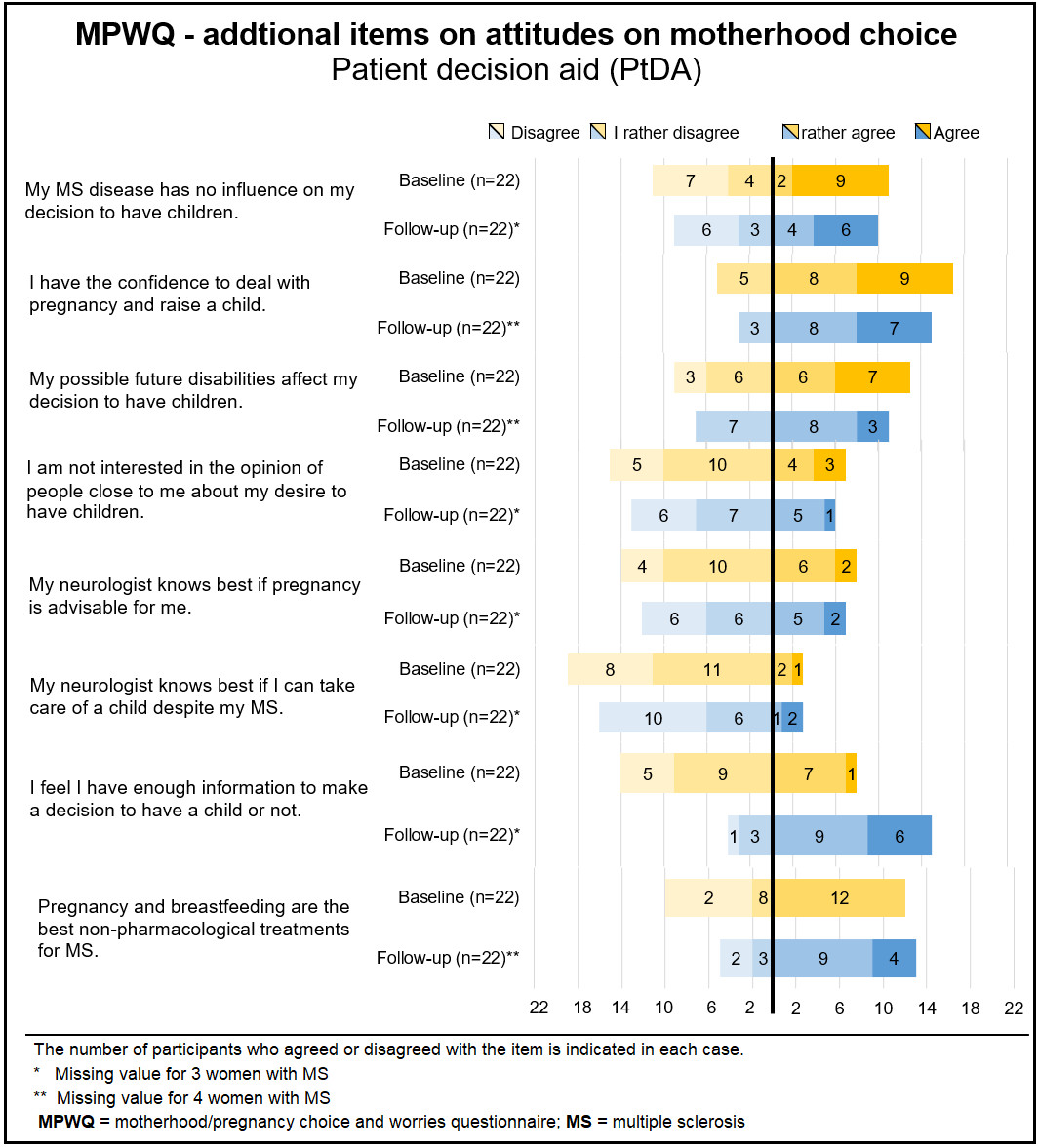


**Fig S4.1. Results of the PtDA group regarding the additional items on attitudes of the motherhood/pregnancy choice and worries questionnaire (MPWQ).**


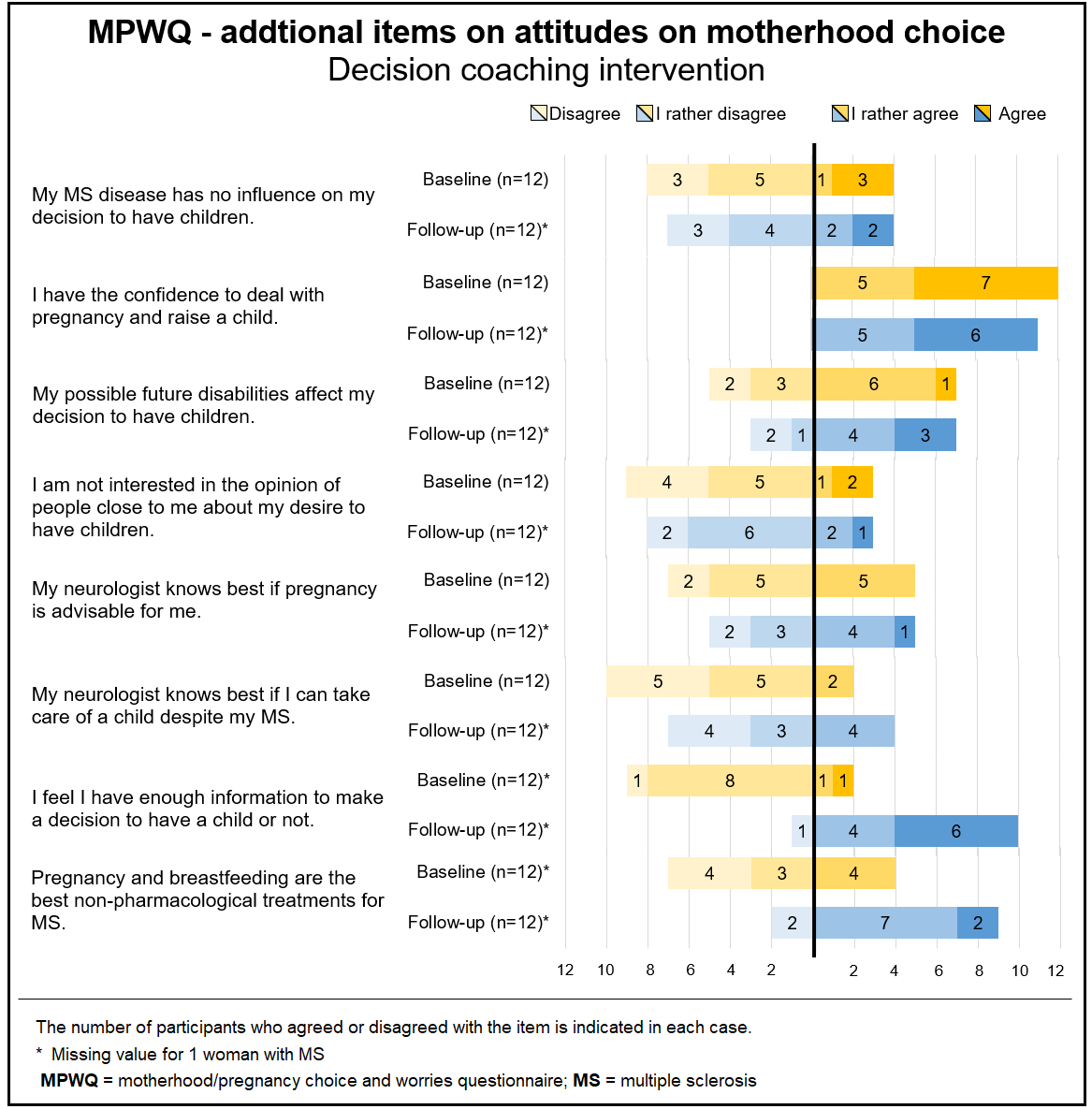


**Fig S4.2. Results of the decision coaching group regarding the additional items on attitudes of the motherhood/pregnancy choice and worries questionnaire (MPWQ).**


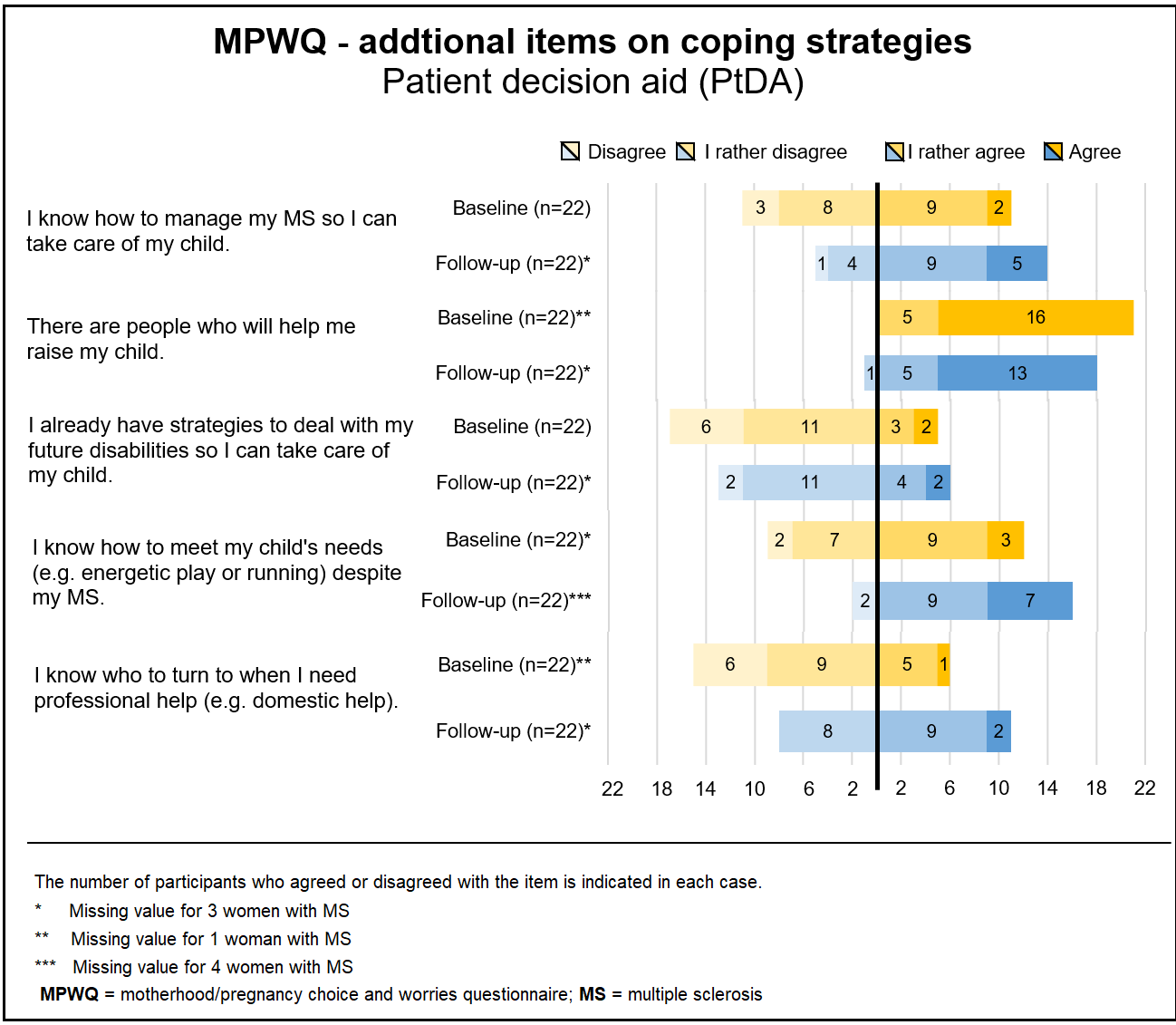


**Fig S4.3. Results of the PtDA group regarding the additional items on coping strategies of the motherhood/pregnancy choice and worries questionnaire (MPWQ).**


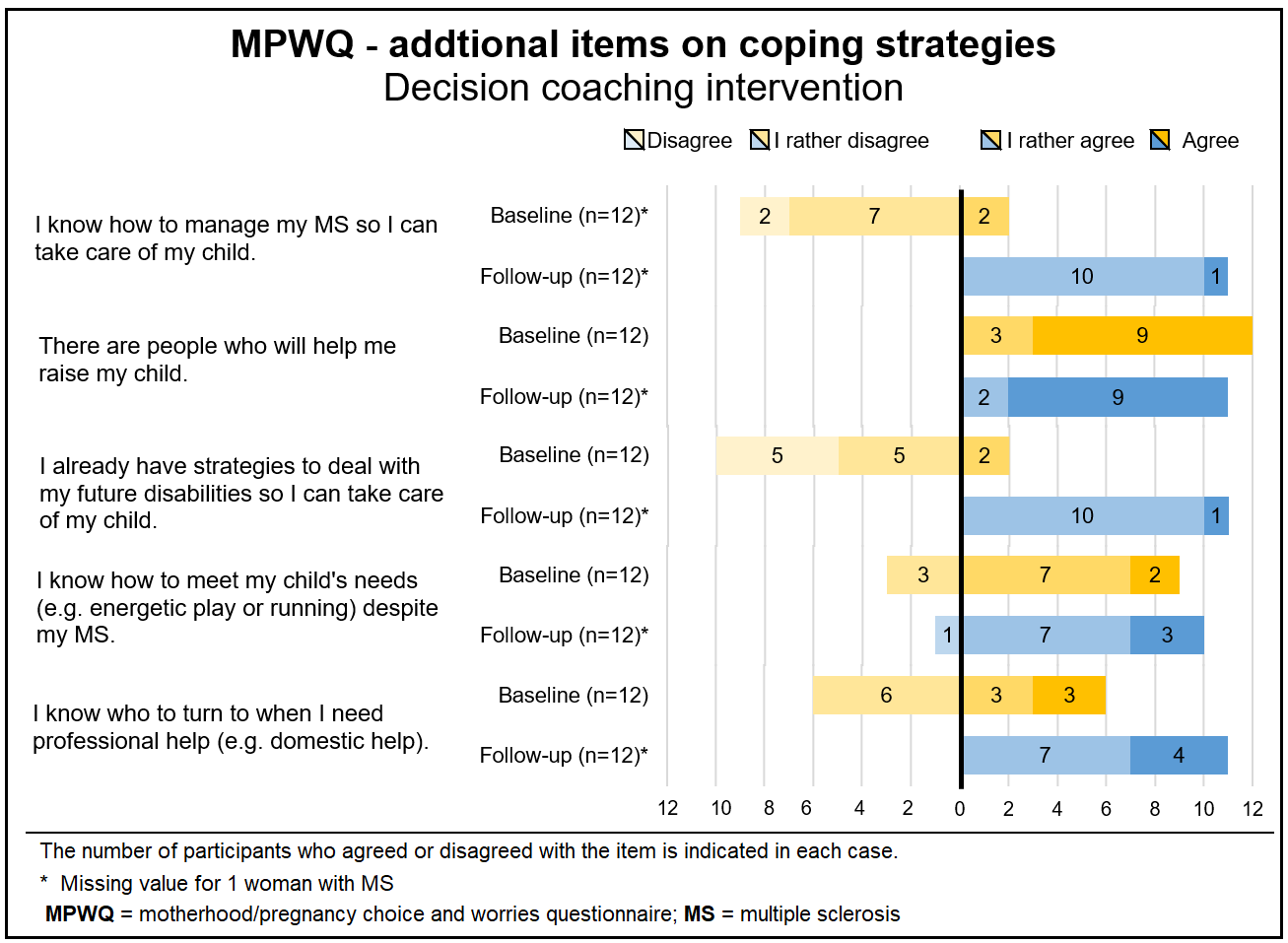


**Fig S4.4. Results of the decision coaching group regarding the additional items on coping strategies of the motherhood/pregnancy choice and worries questionnaire (MPWQ).**
