# Supplementary material for: Motherhood choice in multiple sclerosis (MoMS) – Pilot trial of web-based decision support
Source: PLoS One. 2026 Jun 12;21(6):e0351108. doi: 10.1371/journal.pone.0351108 (PMC13262864; doi:10.1371/journal.pone.0351108)
Supplement: S5 File — (DOCX) [file pone.0351108.s005.docx]

## **S5. Women’s expectations towards the support programme – baseline and follow-up.**


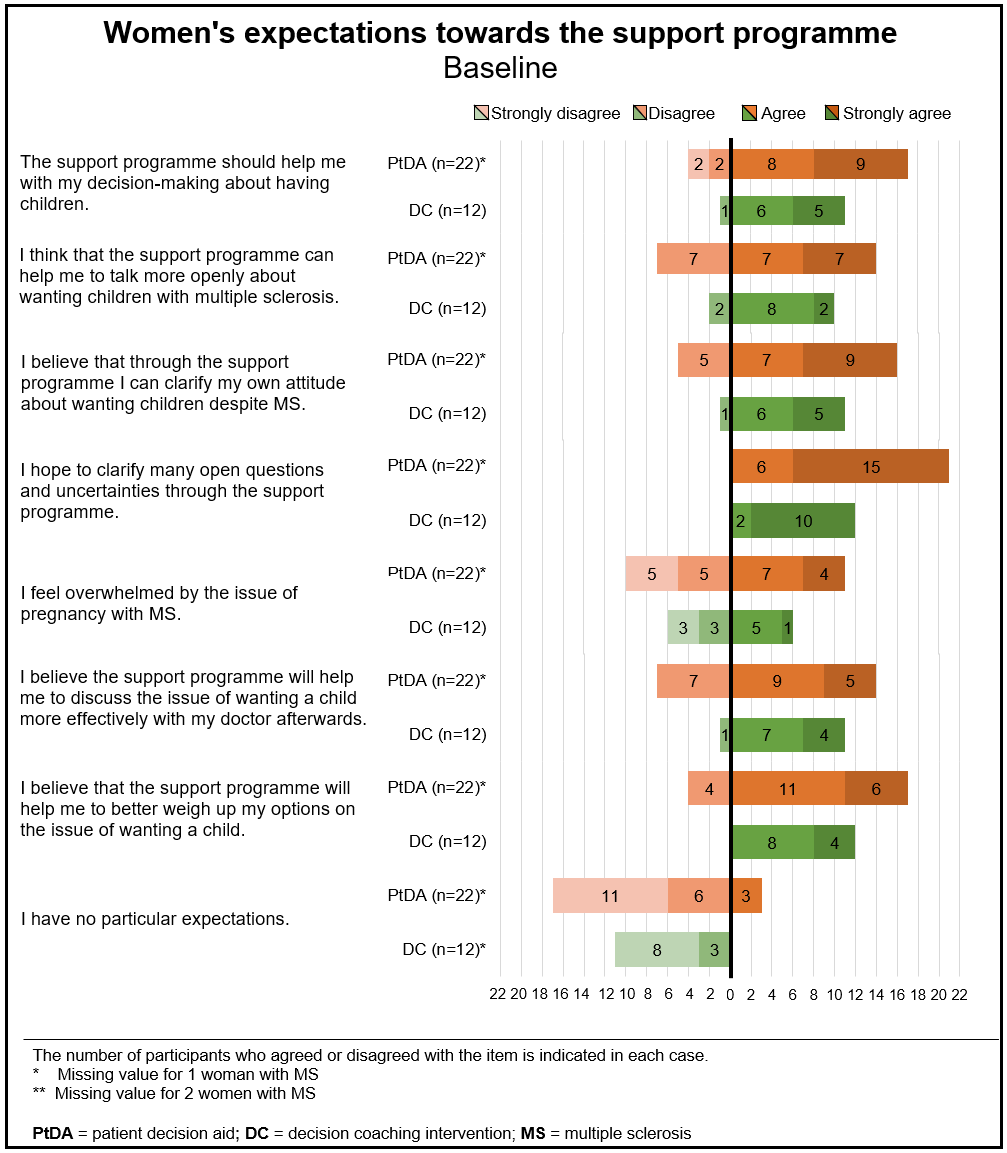


**Fig S5.1. Women’s expectations towards the support programme – baseline.**


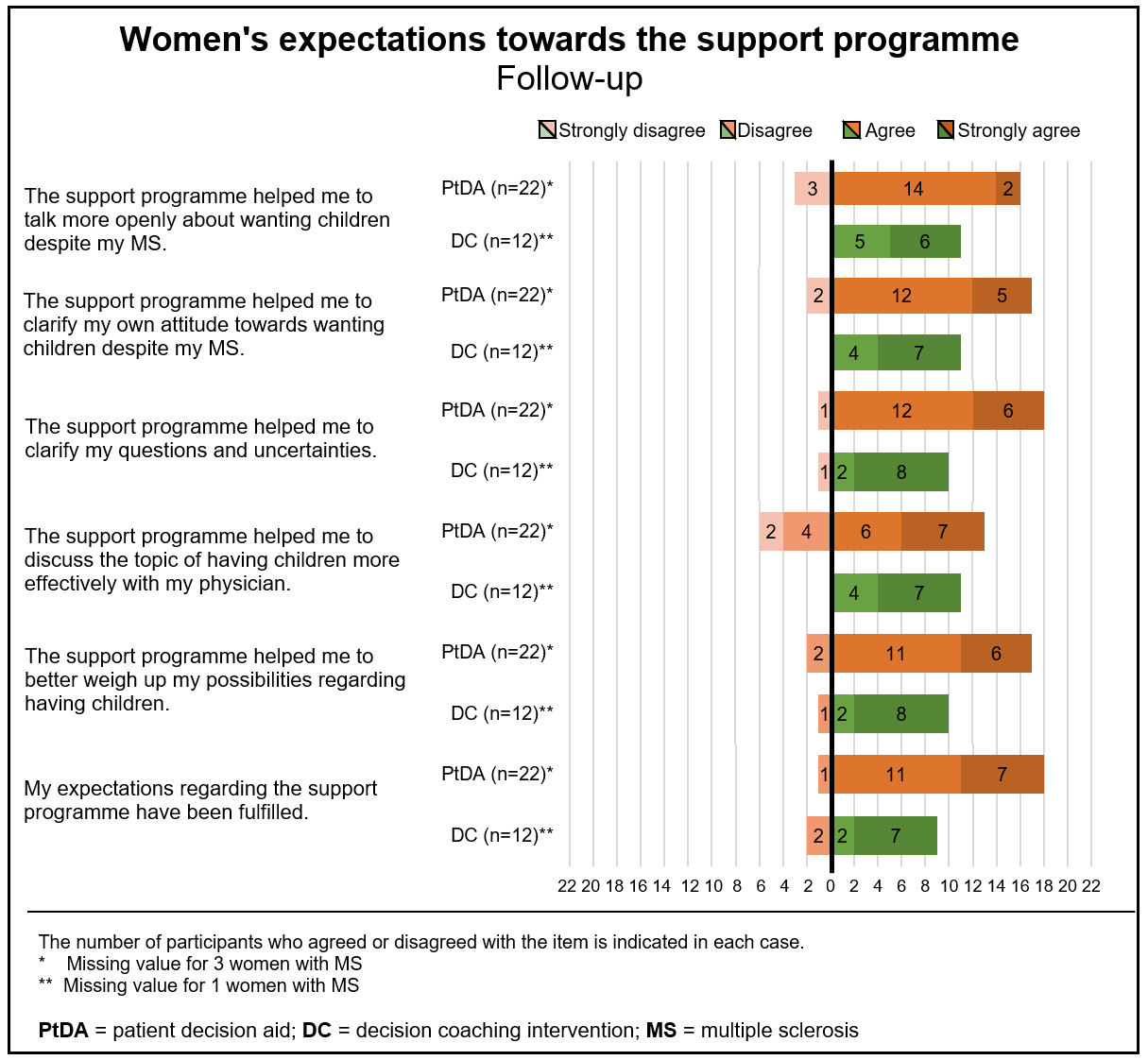


**Fig S5.2. Women’s expectations towards the support programme – follow-up.**
