# Supplementary material for: Motherhood choice in multiple sclerosis (MoMS) – Pilot trial of web-based decision support
Source: PLoS One. 2026 Jun 12;21(6):e0351108. doi: 10.1371/journal.pone.0351108 (PMC13262864; doi:10.1371/journal.pone.0351108)
Supplement: S6 File — (DOCX) [file pone.0351108.s006.docx]

## **S6. Coding templates and summary tables – randomised pilot/ beta testing.**


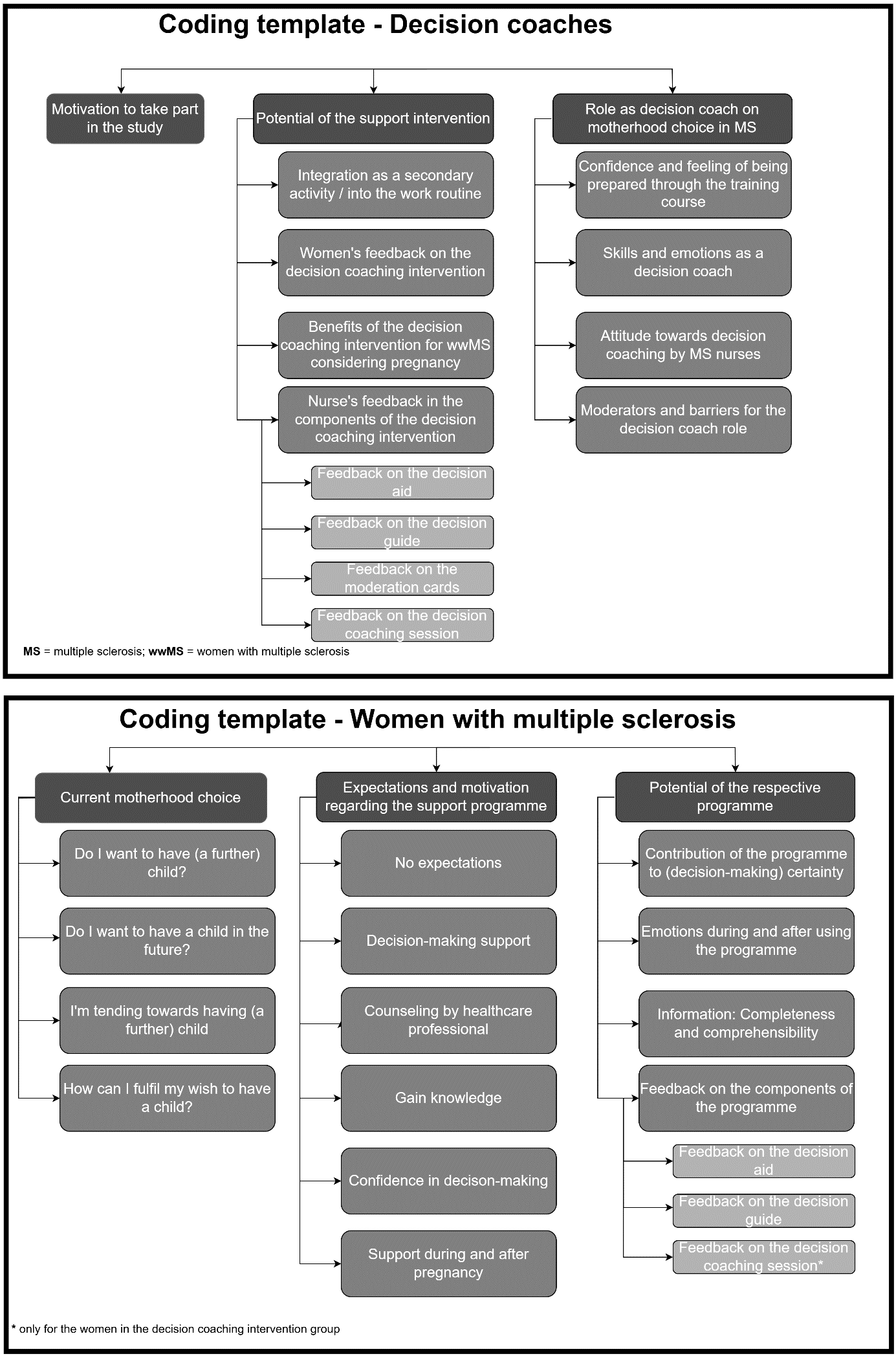


**Fig S6.1. Coding template for the interviews with the women with multiple sclerosis.**

## **Table S6.1. Summary table of the themes with illustrative quotes – women with multiple sclerosis.**

| **Overarching Theme** | **Theme** | **Illustrative quotes** |
| --- | --- | --- |
| **Current motherhood choice** | Do I want to have (a further child? | Interviewer: „Und als Sie dran teilgenommen haben, ging es dabei bei Ihnen um die Frage, möchte ich ein Kind bekommen, ja, nein oder hatten Sie da schon eine Tendenz und haben, ja, Sicherheit gesucht in Form von Informationen?“ WwMS: „Erstes und Drittes würde ich sagen, also ich war mir noch nicht so sicher, ob ich ein Kind haben möchte und natürlich, um Informationen zu bekommen. Also mehr Informationen, als ich bisher hatte dazu.“ *PtDA1*  „Weil ich halt ein Kind schon habe, was ich aber nicht in dem Wissen dieser Erkrankung bekommen habe, sondern da war ich meiner Meinung nach halt noch gesund. Und genau, da war jetzt ein bisschen die Frage, zweites Kind ja oder nein. Ja.“ *DC4* |
|  | Do I want to have a child in the future? | „Das war, glaube ich, ein Beitrag und weil ja bei mir noch ein Kinderwunsch besteht, dachte ich, okay, mache mal mit, kann nicht schaden. Ja und bis jetzt eben noch nicht den richtigen Partner gehabt, aber jetzt kam halt die MS-Diagnose dazwischen, aber ja, ich dachte, mehr Infos oder so kann man nie genug haben. Deswegen habe ich da mitgemacht.“ *DC27* |
|  | I am tending towards having (a further) child | „ … weil ich eigentlich immer hundert Prozent sicher war, Kinder kriegen zu wollen, aber durch den schlechten Verlauf ich dann jetzt eben doch nicht mehr sicher war. Und ja, das eigentlich super gepasst hat jetzt in dem Fall, dass ich dann einerseits die Studie unterstützen kann und andererseits vielleicht für mich da auch noch ein bisschen Klarheit draus ziehen kann.“ *PtDA29*  „Also es ging eigentlich hauptsächlich um mehr Sicherheit. Also war es schon klar, dass wir das beide wollen so und ja, also mein Neurologe meinte auch gleich ganz am Anfang schon: "Ja, Sie brauchen keine Sorgen haben, Sie können auf jeden Fall ein normales Leben führen und auch Kinder kriegen und so." Ja, von daher war es einfach nur mehr Sicherheit und nochmal ein bisschen mehr Background-Wissen sozusagen.“ *DC22* |
|  | How can I fulfil my wish to have a child? | „Und dann eben die Hoffnung hatte, dass ich durch das MoMS-Portal auch Hilfe bekommen, Unterstützung für eben die Entscheidung zu treffen, wann der Zeitpunkt ist. Mein Partner war sich da auch immer sehr unsicher, ob das so funktioniert, ob das alles so gut und ob wir das schaffen können usw., und das hat uns dann doch schon sehr geholfen.“ *PtDA5*  „Also mir ging es so um grundliegende Entscheidungen mit medikamentösen Einstellungen oder wie es mir körperlich gehen könnte in ein paar Jahren. Und da Erfahrungen sammeln von vielleicht auch anderen Müttern.“ *DC10* |
| **Expectations and motivation regarding the support program** | No expectation | „Also, ich hatte jetzt keine hohen Erwartungen. Lag aber daran, dass ich jetzt einfach neutral das gesehen habe und dachte mir, oh, das wird vielleicht mal/ Ich würde mal das so formulieren: Ich habe jetzt nicht gedacht, dass ich jetzt die Lösung aller Probleme da bei dieser Teilnahme jetzt haben werde, also dass was jetzt so sein könnte.“ *PtDA9*  Interviewer: „Gab es noch weitere Erwartungen an das gesamte Programm?“ WwMS: „Nee, tatsächlich jetzt nicht, weil …, bei irgendwas mal mitzumachen und weiß auch nicht, wie sowas sonst abläuft. Aber ich war einfach nur gespannt, ob ich viel Neues erfahre, was auf mich zukommt, vor allem von dem Gespräch, von diesem Coaching und war da auch sehr positiv angetan von der Dame, die das mit mir geführt hat, ja.“ *DC32* |
|  | Decision-making support | „Und, genau, dann habe ich mich entschieden halt bei dieser Studie mitzumachen, da ich auch bei Power-MS das Portal oft genutzt habe, um etwas nachzulesen. Und dann eben die Hoffnung hatte, dass ich durch das MoMS-Portal auch Hilfe bekommen, Unterstützung für eben die Entscheidung zu treffen, wann der Zeitpunkt ist.“ *PtDA19* |
|  | Counseling by healthcare professional | „Und die Motivation war eigentlich eher, dass man nochmal so Kontakt vielleicht auch zu entsprechenden Spezialisten hat, die vielleicht auch nochmal, ja, Hilfestellungen oder Rat geben können, vielleicht einfach für den Verlauf oder die Ängste und Sorgen, die halt damit einfach konfrontieren mit der, also mit einer Autoimmunerkrankung und entsprechendem Kinderwunsch. Weil ich halt ein Kind schon habe, was ich aber nicht in dem Wissen dieser Erkrankung bekommen habe, sondern da war ich meiner Meinung nach halt noch gesund. Und genau, da war jetzt ein bisschen die Frage, zweites Kind ja oder nein.“ *DC4* |
|  | Gain knowledge | „Vielleicht, ja, wirklich mehr Information nochmal zu bekommen, die Infos alle so gedeckelt zu kriegen also, dass man sich nicht durch 25 verschiedene Seiten im Internet klicken muss, sondern, dass man dann eine Seite hat, auf die man zugreifen kann und wo man sich dann durch die verschiedenen Themenschwerpunkte durchklicken kann und eben ganz einfach und komplikationslos an fundierte Infos kommt.“ *PtDA29*  „Nee, tatsächlich jetzt nicht, weil …, bei irgendwas mal mitzumachen und weiß auch nicht, wie sowas sonst abläuft. Aber ich war einfach nur gespannt, ob ich viel Neues erfahre, was auf mich zukommt, vor allem von dem Gespräch, … .“ *DC32* |
|  | Confidence in decision-making | „Ja und das beschäftigt einen dann halt schon, gerade, weil ich eigentlich immer hundert Prozent sicher war, Kinder kriegen zu wollen, aber durch den schlechten Verlauf ich dann jetzt eben doch nicht mehr sicher war. Und ja, das eigentlich super gepasst hat jetzt in dem Fall, dass ich dann einerseits die Studie unterstützen kann und andererseits vielleicht für mich da auch noch ein bisschen Klarheit draus ziehen kann.“ *PtDA29* |
|  | Support during and after pregnancy | „Ja, ich habe so ein bisschen erwartet oder gedacht, dass man so vielleicht auch so im Verlauf so ein bisschen im Kontakt ist mit eventuellen Ansprechpersonen. Also das war jetzt so im Voraus auch nicht so ganz klar und wurde jetzt auch nicht so ganz deutlich, ob man jetzt/ Ich wusste zum Beispiel jetzt auch nicht, soll ich mich jetzt nochmal melden jetzt, wo ich schwanger bin, ist auch so ein bisschen der Zwiespalt. … . Das war mir halt alles nicht so richtig bewusst und da hätte ich mir so ein bisschen gewünscht, dass man da vielleicht noch so im weiteren Austausch einfach bleibt oder was zumindest so, ja, so der Nachgang dieses Gespräches oder dass man dann entsprechend nochmal irgendwie Infos bekommt, wie das Ganze dann so weiter läuft, ja. Aber ansonsten hatte ich erstmal ja nicht die Erwartung, dass ich da jetzt da direkt so eine Begleitung während der Kinderwunschzeit sozusagen bekomme. “ *DC4* |
| **Potential of the respective program** | Contribution of the program to (decision-making) certainty | „Durch die Information, die man jetzt zusätzlich jetzt bekommen hat, finde ich, ist man ja aufgeklärter. Und das gibt schon Sicherheit, finde ich. Also man kann ja jetzt sagen: "Ich weiß das und das schon" oder "durfte das kennenlernen, die Information." Ja. Also es hat schon Sicherheit gebracht, finde ich, die Information in der Hinsicht. Das ist halt in der Entscheidung noch bisschen schwierig, wo ich aber nicht ganz genau sagen könnte, "ja, ich will es unbedingt", weil meine Angst halt da noch zu groß ist wegen den Schüben. Also das beherrscht mich noch mehr gerade, als den Wunsch. So doof das klingt.“ *PtDA1*  „Ich würde/ Also, wie gesagt, ich habe jetzt das mir mal so grob durchgelesen. Es war ganz interessant, aber ich habe ehrlich gesagt/ wenn Sie mich jetzt nach vier Wochen oder drei Wochen wieder fragen, wüsste ich jetzt gar nicht mehr, wie genau es aufgebaut war und was ich jetzt davon/ ich habe mir ein paar/ zumindest habe ich so grobe Zahlen im Kopf gehabt, aber die habe ich jetzt inzwischen auch wieder vergessen, deswegen würde ich auch eher sagen, es wäre vielleicht irgendso ein persönlichen Gespräch oder irgendwie so ein Telefongespräch wäre, glaube ich, sinnvoller, um darüber zu/ damit man sich das auch merkt als wenn ich mir das jetzt mal schnell durchlese. Irgendso für mich mal schnell nebenbei durchzulesen.“ *PtDA9*  „Also mir hat es wirklich sehr geholfen, also natürlich hat man vorher schon irgendwo eine Präferenz, dass man sagt: "Mensch, man würde eigentlich Kinder haben wollen, aber es war schon noch, dass ich sage, ja, irgendwo abhängig, dass ich sage, ich möchte mich mit den Informationen auseinandersetzen. Ich möchte wissen, welche Möglichkeiten ich habe während der Schwangerschaft Medikamente zu nehmen, ob es überhaupt die Möglichkeit gibt, et cetera. Und von dem her war es wirklich, sage ich mal, wichtig, dieses geballte Wissen irgendwo, ja, zur Verfügung gestellt bekommen zu haben und mich deswegen entscheiden zu können. “ *DC21*  „Also ich denke gerade, wenn man sich noch nicht so ganz sicher ist oder eben, wenn man, ja, also ich finde immer, wenn man sich mit dem Thema auseinandersetzt, dann gibt es halt viele Infos und das ist einfach alles so, ja, prägnant und informativ. Und deswegen fand ich es eigentlich sehr gut und das unterstützt einen schon in der Entscheidung.“ *DC22* |
|  | Emotions during and after using the program | „Ich muss sagen, kurz danach habe ich mir paar Gedanken darüber gemacht, ob das irgendwie einen Zusammenhang hat, weil das was ich mitgenommen habe ist, dass es/ das Kinderwunsch oder generell Familienplanung schon prozentual gesehen Einfluss drauf hat. Dann hat man sich schon bisschen Gedanken darüber gemacht, aber, ich sage mal, am nächsten Tag, oder so, habe ich das auch schon wieder vergessen oder ausgeblendet.“ *PtDA9*  „Genau, man ist einfach ruhiger, weil man weiß, es gibt Handlungsoptionen, es gibt Möglichkeiten, es ist einfach nicht das große Abschlusskriterium und ja. Man fühlt sich sicherer. Also ich fühle mich sicherer durch das Wissen. Mich macht Wissen sowieso sicher. Deswegen war das für mich wirklich ein wichtiger Punkt, dass ich das einfach abklären konnte, ohne dass ich mich dafür immer erklären muss, dass ich diese Fragen habe.“ *PtDA20*  „Ja, das ist tatsächlich immer ein Thema, das ist super informativ, aber gleichzeitig von der Stimmung her zieht es dich runter. Es geht mir aber, egal über was ich, wenn es um die MS geht, wenn ich dann sehe, es zieht mich immer runter. Für mich ist die Devise, nur das Nötigste oder mit der MS nur das Nötigste mich auseinanderzusetzen, weil es mir dann psychisch besser geht.“ *DC32*  „so das hat mir so von den Dingen, die jetzt da angeboten wurden, also Entscheidungsleithilfe, auch nochmal die Homepage und das persönliche oder telefonische Gespräch. Das hat mir eigentlich auch nochmal so ein paar Ängste und Sorgen genommen oder auch zumindest das Gefühl gegeben so, man wird verstanden und man ist da irgendwie auf dem richtigen Weg.“ *DC4* |
|  | Information: Completeness and comprehensibility | Interviewer: „Hat Ihnen etwas für die Entscheidungsfindung gefehlt?“ WwMS: „Wir haben uns alle Punkte gut durchgelesen. Wir hatten da unterschiedliche Schwerpunkte für uns persönlich auch, und Sie haben uns beiden sehr weitergeholfen. Also, es war jetzt nichts, wo wir dachten: Boah, da möchte ich mich aber nochmal nachlesen. Und irgendwie fehlt das da. (...) Oder dazu habe ich jetzt keine Information gefunden, oder so. Also, da gab es keinen Punkt. Bei keinem von uns.“ *PtDA19*  „Nee, das meiste waren eigentlich Informationen auch über mein Medikament, ob das möglich ist, eine Schwangerschaft, inwiefern Risiken bestehen, worauf man achten muss und so. Und das ist eigentlich, ja, was man so im Internet nicht findet, ist mehr oder weniger alles beantwortet worden.“ *DC27* |
|  | Feedback on the components of the program | **Feedack on the decision aid**  „Also ich fand es sehr gut, ich fand spannend, ich habe mich da auch an zwei Abenden mit beschäftigt, einmal alleine und einmal dann zusammen mit meinem Freund. Und ich fand, dieses Unterstützungsprogramm hat mir ein bisschen mehr geholfen, als dieser Leitfaden. Also ich habe mich durch diese Homepage geklickt. Und habe die verschiedenen Kapitel durchgelesen. Teilweise kannte ich die Informationen auch schon. Teilweise hatte ich da noch nichts von gehört oder das hat sich gegenseitig ergänzt. Und von daher, ich fand es echt gut, ich habe da viel draus mitnehmen können aus den beiden Dingen, wobei ich persönlich jetzt die Homepage für mich besser fand noch, als den Leitfaden.“ *PtDA20*  „… ich finde, dass das Programm sehr gut aufgebaut ist, sehr verständlich ist, sehr leicht zugänglich ist, also es ist kein Wirr Warr mit tausenden Unterseiten, oder so, sondern es ist alles klar und strukturiert aufgebaut und verständlich auch formuliert und geschrieben, sodass auch jemand, der da jetzt nicht so viel Ahnung von medizinischen Fachbegriffen hat, das gut verstehen kann. Also, das hat sowohl mein Partner verstanden, der damit nicht so viel zu tun hat, wie ich, die mich schon viel mit der Krankheit überhaupt beschäftigt hat und auch aus dem Gesundheitsbereich komme, sodass ich da so ein bisschen Fachchinesisch auch verstehe. Genau, von daher hat das da echt alle Erwartungen erfüllt.“ *PtDA19*  „Ich fand das sehr gut und vor allem sehr übersichtlich. Dieses Onlineportal quasi, das fand ich sehr gut, da das, ja, das Wichtigste direkt herausgestochen ist vom Design auch her. Also man musste sich nicht ewig durch die Seiten quälen, um die richtigen Infos zu bekommen. Das war immer kurz und knapp auf den Punkt gebracht. Das fand ich sehr gut.“ *DC10*  „Und die Online-Plattform an sich fand ich auch richtig, richtig gut, weil ich selbst google mal und möchte irgendwie zu einem Thema was wissen, kann man da stillen oder welche Medikamente kann ich nehmen. Und für mich muss ich wirklich sagen, hat sich dadurch einfach, ja, das erleichtert, dass ich alle Informationen an einem Ort hatte und nochmal nachlesen konnte und wirklich aus fundierten, ja, Quellen, wo ich mir sicher bin, das stimmt jetzt auch. Und ich meine, sonst im Internet muss man natürlich schon nochmal, ja, irgendwie drauf achten, ist es zuverlässig, et cetera? Und da konnte ich mich einfach darauf verlassen, dass ich weiß, da kann ich eben, ja, vertrauen und konnte die Informationen bekommen, die ich gesucht habe.“ *DC21*  **Feedback on the desicion guide**  „Also leider muss ich sagen, ich glaube, den Leitfaden, den habe ich nur grob überflogen und hatte nicht so den Bezug zu mir dafür gefunden. Und das andere fand ich sehr gut, fand ich sehr gut aufgebaut, sehr gut, dass alle Themen mit angesprochen wurden, fand ich sehr gut. (...) Sehr hilfreich.“ *PtDA1*  „Der bietet einfach die Möglichkeit, dass all das, was man aufgenommen hat, nochmal in kurzer Übersicht und für sich jede Frage gezielt einfach oder jedes Themengebiet einfach einmal in Übersicht zu beantworten oder zu sehen. Wie soll man das beschreiben? Und gerade, weil es dann ja auch viele Informationen sind, einfach nur zu sehen übersichtlich, das ist die Faktenlage. Die Möglichkeiten bestehen. Die Medikamente ja, die Medikamente nein. Ja. Einfach, selbst wenn man dann nochmal irgendwie was nachlesen will oder so oder einfach nochmal für sich kurz gucken will, kann man da eben einfach mal draufgucken und sagen: "Ja, so ist es." Oder man kann sich vielleicht sogar ausdrucken, weiß ich nicht: Ich sage mal so: Wenn man jetzt mit seinem Partner im Gespräch ist und er jetzt vielleicht nicht so ein Thema ist oder Ähnliches, kann man nicht auch geben. Für ihn ist es vielleicht nochmal leichter, das so im Überblick zu sehen und man kann dann ein Gespräch darüber führen, eine Diskussion führen. Weil ich gehe jetzt natürlich/ Also ich habe jetzt wirklich die komfortable Luxussituation, mit meinem Partner wirklich viel geredet zu haben, viel sich darüber ausgetauscht zu haben. Und er ist nicht betroffen von der Erkrankung. Aber für ihn waren eben Kinder auch sehr wichtig.“ *PtDA20*  „Ich fand den angenehm, vor allem halt im Austausch mit Partner oder Familie, da man da nochmal so die Standpunkte vergleichen konnte in einem kurzen/ Ja, das war jetzt nicht so groß, deswegen konnte man das gut besprechen gemeinsam.“ *DC10*  „Also es waren schon etliche Infos dabei, die mich beruhigt haben, andererseits in diesem Decision Guide werden ja auch so Gegenüberstellungen gemacht von Faktoren, die negativ oder, ja, negativ für eine Schwangerschaft oder für eine Entscheidung gegen die Schwangerschaft sein könnten. Das war dann, wenn man das so plastisch vor sich sieht, war das schon ernüchternd, sage ich mal. Aber die Informationen waren sehr gut.“ *DC27*  **Feedback on the decision coaching session**  „vor allem von dem Gespräch, von diesem Coaching und war da auch sehr positiv angetan von der Dame, die das mit mir geführt hat, ja.“ *DC32*  „Alles in allem wirklich super, wobei ich wirklich sagen muss, dass das Coaching natürlich an sich nochmal, ja, das i-Tüpfelchen war und wirklich für mich mit am besten, weil man einfach mit einer Person persönlich nochmal drüber sprechen kann. Jemand, der sich mit dem Thema selbst auseinandergesetzt hat, der auch die richtigen Fragen vielleicht nochmal stellt oder auch sichergeht, ob man alles richtig verstanden hat.“ *DC21*  „Also was ich vielleicht gut fände, also jetzt für, nicht jetzt unbedingt für meinen Fall, aber so grundsätzlich, glaube ich, hätte ich es gut gefunden, wenn das vielleicht so zwei Gespräche mit ein bisschen Abstand gewesen wären. Also, sodass man, gerade wenn große Unsicherheiten bestehen, vielleicht dann erstmal so ein Aufklärungsgespräch bekommt und dann nochmal so ein Reflexionsgespräch zum Beispiel angeboten wird. Ich glaube, das wäre nochmal für so Entscheidungen, die man dann wirklich trifft, vielleicht nochmal sinnvoller gewesen, vielleicht so im Abstand von, ich weiß nicht, zwei oder vier Wochen, dass man nochmal drüber nachdenken kann, wenn man jetzt wirklich vielleicht wirklich große Zweifel hat, ob man noch, ja, Kinder bekommen möchte oder nicht.“ *DC4*  Interviewer „Wie empfanden Sie das gewählte Medium zur Durchführung des Decision Coachings? Bei Ihnen wurde das ja per Telefon durchgeführt.“ wwMS: “Fand ich gut. Also wer das mit Video machen will, der kann das bestimmt auch mit Video machen, aber Telefon war für mich in Ordnung.“ *DC27* |

PtDA = Patient decision aid, DC = decision coaching intervention


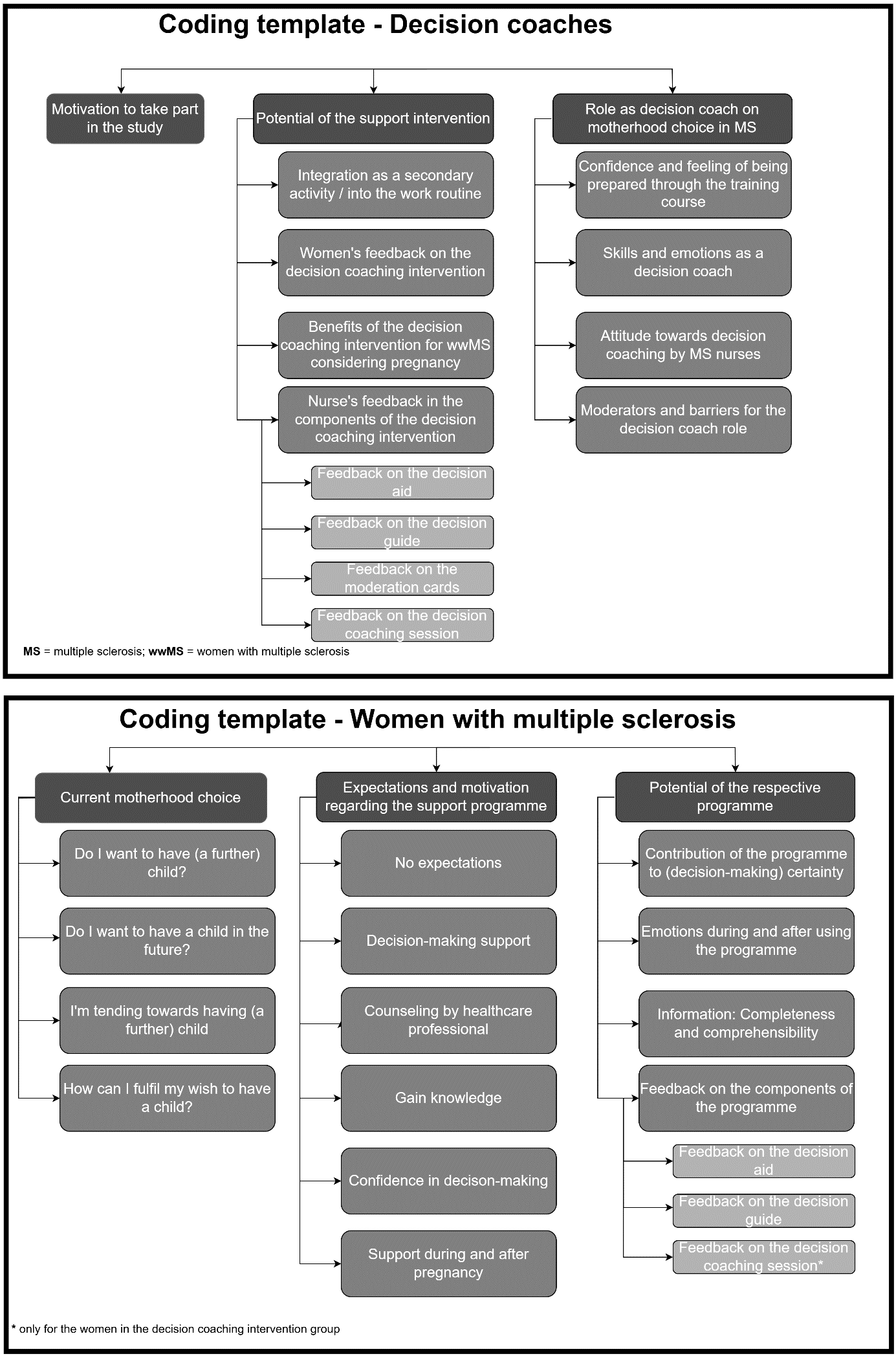


**Fig S6.2. Coding template for the interviews with the decision coaches.**

## **Table S6.2. Summary table of the themes with illustrative quotes – decision coaches.**

| **Overarching Theme** | **Theme** | **Illustrative quotes** |
| --- | --- | --- |
| **Motivation to take part in the study** |  | „Sie [Arbeitgeber] hat mich gefragt, ob ich, weil ich ja sowieso schon Frauen berate beziehungsweise interviewe, ob ich nicht Lust habe und das interessant finden würde, an dem Projekt teilzunehmen. Das fand ich sehr interessant, einfach zu gucken, wie die Frauen im Vorfeld vielleicht schon damit umgehen, mit der Erkrankung und mit Schwangerschaft und so weiter und ob ich da nicht unterstützend dazu beitragen kann, dass eine Schwangerschaft entsteht, ohne Angst und Sorge. Das fand ich spannend.“ *Nurse1*  „Also warum ich teilgenommen habe, weil ihr mich gefragt habt und [Arbeitgeber] gefragt habt, ob ich da nicht Lust zu habe sozusagen, so. Genau, deswegen habe ich teilgenommen. (...) Und MS und Erfahrung und Schwangerschaft kann ich ja eigentlich nur sagen, dass es ja in [Projektname] ja öfter auch mal wieder auftaucht, wenn da Frauen eben mal sagen, dass sie aber noch Kinderwunsch haben und wir hier die Schwanger-MS-Kohorte ja betreuen, ne?“ *Nurse2* |
| **Potential of the support intervention** | Integration as a secondary activity / into the work routine | „Das ist ja, ich habe ja auch so eine beratende Tätigkeit da, das ist ähnlich und ich telefoniere da viel mit Frauen. Also es ist mir nicht schwergefallen und es hat mich auch nicht irgendwie großartig belastet. Ich sage das mal so, neben meiner jetzigen Tätigkeit. Also das spielte schon gut zusammen.“ *Nurse1*  „Ja, soweit der Chef mir dafür Zeit einräumt, sehr gerne. Ja, je nachdem, wie der Schwerpunkt in den Ambulanzen darauf gelegt wird, ne? Aber auf jeden Fall kann ich mir das weiter vorstellen, das zu machen.“ *Nurse2* |
|  | Women’s feedback on the decision coaching intervention | „Also die haben eigentlich durchweg positiv reagiert. Ja, also die waren sehr dankbar für die ganzen Informationen und auch für die Fragen, die sie stellen durften und auch beantwortet bekommen haben und sind eigentlich nach dem Coaching gestärkt rausgegangen, auch im Rahmen des Pilotcoachings.“ *Nurse1*  „so ist MoMS ja auch, es ist eine dankbare Arbeit. Die Patienten sind meistens sehr dankbar. Nee, nicht meistens, eigentlich alle sind sehr dankbar, dass es jemanden gibt, der sich Zeit nimmt, dass man das mal mit denen aufdröselt, so.“ *Nurse2* |
|  | Benefits of the decision coaching intervention for wwMS | „Also die sind schon sehr viel positiver in Bezug jetzt auf eine bevorstehende Schwangerschaft rausgegangen. Viele waren sehr unsicher und diese Unsicherheiten, die noch da waren, die konnten geklärt werden und ich habe am Schluss auch immer gefragt, ob das Coaching für sie positiv war und was gebracht hat und die haben alle gesagt, das war so toll, ich bin jetzt in meiner Entscheidung nochmal ein bisschen weiter.“ *Nurse1*  „Ich glaube, dass denen das hilft sozusagen, also dass es denen wahrscheinlich schon mehr hilft, als nur das Onlineportal zu haben, so, weil sie irgendwie das Gefühl haben, da ist nochmal jemand, der das wirklich nur wissen will, wie es bei mir ist und wie es um meine Belange steht und hier kann ich nochmal irgendwie sozusagen alles sagen. Und es ist ja eher so, im ganzen Gesundheitswesen, dass die Leute eher nicht viel Zeit haben. Und da haben sie mal, quasi ist dann jemand sozusagen, so. Ich glaube, dass sie das auch, die reinen Fakten sozusagen auch mitkriegen könnten, also nur durch das Onlineportal. So. Dass sie es aber so, sage ich jetzt mal, so psychologisch gesehen, glaube ich, ist das total sinnvoll.“ *Nurse2* |
|  | Nurse’s feedback on the components of the decision coaching intervention | **Feedack on the decision aid**  „Das hat einen sehr großen Mehrwert zu dieser Plattform und da stehen natürlich alle Informationen drin, die auch in diesem Coaching vorkommen.“ *Nurse1*  „Ich glaube, dass sie das auch, die reinen Fakten sozusagen auch mitkriegen könnten, also nur durch das Onlineportal. So. Dass sie es aber so, sage ich jetzt mal, so psychologisch gesehen, glaube ich, ist das total sinnvoll.“ *Nurse2*  **Feedback on the desicion guide**  „Also ich finde diesen Decision -Guide schon recht hilfreich, um den roten Faden zu behalten, dafür ist es ja auch da. Und dass einfach die Frauen mitgucken können, wo sind wir grade, welche Fragen können aufgeschrieben werden, was können die hinterher noch benutzen und so weiter. An einigen Stellen fand ich es für mich jetzt ein bisschen schwierig, aber im Großen und Ganzen ist das schon sehr hilfreich gewesen, um das Gespräch zu gestalten einfach, ne? Mehr fällt mir da jetzt grad nichts zu ein.“ *Nurse1*  „Das und irgendwie, genau, dann mit diesen Möglichkeiten, ich finde das einfach total schwierig das zu formulieren und nachher habe ich schon gesagt, wenn die fragen: "Hä? Wie meinen Sie das jetzt?" Wo ich auch schon zu denen gesagt habe: "Ja, ich finde es auch irgendwie schwierig." Also so, was ist denn die Möglichkeit? Ein Kind zu bekommen oder keins zu bekommen? Oder so? Oder was? Ich finde es einfach total schwierig, irgendwie diese Möglichkeiten zu benennen, wenn es eigentlich dann nur zwei gibt, bekomme ich jetzt ein Kind oder bekomme ich keins oder bekomme ich ein weiteres oder kein weiteres sozusagen und auch dann noch mit dieser Bewertung, dann noch, dass die für sich bewerten. Also ich hatte da auch das Gefühl gehabt, dass die auch Schwierigkeiten haben, aber ich habe es natürlich auch nicht gut verkaufen können, wie sie es jetzt/ Weil mir das selber nicht so richtig irgendwie einleuchtet.“ *Nurse2*  **Feedback on the moderation cards**  „Also diese Moderationskarten sind das ja, ne? Die waren total hilfreich. Da sind ja auch Formulierungsbeispiele mit drauf gewesen und so weiter und das hat mich total unterstützt, das war richtig gut. Und ja, ich hatte zu Anfang so ein bisschen Schwierigkeiten, tatsächlich habe ich mich an diesem Bogen, den die Frauen auch hatten, so ein bisschen orientiert. Das war aber nicht gut, weil die Reihenfolge da anders ist, ne? Deswegen, aber die Moderationskarten haben mich total unterstützt und die waren richtig klasse gewesen.“ *Nurse1*  „Also finde ich, ich finde das mit den Moderationskarten so auch ganz gut und hilfreich.“ *Nurse2*  **Feedback on the decision coaching session**  „Das hat einen sehr großen Mehrwert zu dieser Plattform und da stehen natürlich alle Informationen drin, die auch in diesem Coaching vorkommen. Aber es ist schon was anderes und das hat mir eine Frau auch wirklich deutlich gesagt, ob man das nur für sich alleine liest oder ob man das erklärt bekommt, ne? Und dass man da vielleicht auch nochmal Rückfragen haben kann, wenn Unklarheiten da sind, dass man das einfach nochmal besser erklärt bekommt halt, ne? Genau. Das ist unabdinglich sozusagen.“ *Nurse1*  „Ich weiß nicht, ob man irgendwie nochmal abfragen müsste, wie gut sind sie jetzt eigentlich aufgeklärt über die MS oder ist das Krankheitsbild denen jetzt überhaupt klar, was denn passiert. Ist das jetzt überhaupt wichtig für diesen Schwangerschaftsteil? Ja irgendwie schon, man muss ja wissen, womit man zu tun hat. Wenn man jetzt nochmal anfängt, … , die ganzen Diagnosekriterien, das führt ja ein bisschen weit sozusagen. So. Aber ich hatte bei einigen so das Gefühl, dass denen eigentlich auch nicht so ganz klar ist, was bedeutet jetzt eigentlich Schübe. So und wenn denen eigentlich nicht klar ist, was sind eigentlich Schübe, können sie es eigentlich auch nicht einschätzen, was es bedeutet, wenn sie ein kleines Kind haben und dann Schübe haben oder irgendwie so.“ *Nurse2* |
| **Role as decision coach on motherhood choice in MS** | Confidence and feeling of being prepared through the training course | „Das Training, das war sehr gut, weil man das dann von vorne bis hinten natürlich einmal durchgesprochen hat, mit den Frauen und konnte dann selber gucken, wo hakt das bei mir, wo bin ich unsicher, wo muss ich nochmal besser gucken. Und dann mit dem Feedback natürlich auch, das ich bekommen habe, was da eben verbessert werden kann oder was gut war oder so. Das habe ich dann für das nächste Gespräch immer mitgenommen und das war eine super Sache, mit den Gesprächen“ *Nurse1*  „Aber in der Schulung habe ich erstmal, also das war ja auch irgendwie, auch die Beispiele, die wir da hatten, die waren ja manchmal gar nicht so einfach, diese Übungsbeispiele. Und ja genauso ist es ja aber auch in der echten Welt. Also weil man ja auch von bis, mit Therapie und auch mit, die letzte hatte ja auch Chemos und so, ist ja auch alles nicht so ohne. Also da war ja schon irgendwie eigentlich alles dabei sozusagen, weil wir damals in der Schulung gesagt haben, dieses Beispiel, schon mit Therapie und so, das wäre gar nicht drauf gekommen.“ *Nurse2* |
|  | Skills and emotions as a decision coach | „Im Gespräch mit den Frauen? Ach, das war alles immer ganz entspannt und war angenehme Atmosphäre. Die Frauen waren sehr interessiert, was natürlich sehr geholfen hat bei dem Gespräch. Einige waren vorab auch schon selber informiert, sodass da gar nicht mehr so viel/ Es mussten nur noch kleinere Fragen geklärt werden, ich sage das mal so und ich habe mich da eigentlich ganz wohl gefühlt. Und die Frauen, die waren sehr dankbar für die Informationen, die sie bekommen haben und für die Unterstützung, die sie durch dieses Coaching erfahren haben.“ *Nurse1* |
|  | Attitude towards decision coaching by MS nurses | „Es ist, glaube ich, schon was anderes für die Frauen, wenn sie mit geschultem Personal sprechen, als wenn sie jetzt vor Ärzten sitzen und in Arztpraxen sind, da ist ja oft Zeitdruck da und ja, vielleicht traut man sich da auch nicht so richtig so intensiv zu fragen. Wahrscheinlich aufgrund des zeitlichen Problems in Praxen, ne? Und deswegen ist das schon hilfreich und sinnvoll, also doch, dass das dann jemand macht, der sich die Zeit dafür auch nehmen kann.“ *Nurse1*  „Ach gut. Also ich, prinzipiell mache ich das ja gerne mit dem Decision-Coaching und fand auch so, also die Frauen waren alle nett sozusagen. Also es war immer eine nette Gesprächsatmosphäre, also die haben ja auch alle freiwillig teilgenommen, das hat ihnen ja keiner aufgedrückt sozusagen. So. Also ich hatte jetzt nicht das Gefühl irgendwie oder ich hatte auch nicht das Gefühl, da jetzt irgendwie unsicher zu sein oder wenn ich da irgendwie selber nicht genau wusste, wo man das findet, also ich habe jetzt auch kein Problem das zu benennen oder auch zu sagen, ist ja ein neues Ding hier, muss ich selber erstmal gucken oder irgendwie so. Also so. Nee, ich sage mal so, rundherum gut.“ *Nurse2* |
|  | Moderators and barriers for the decision coach role | Interviewer: „Gab es vielleicht Faktoren, die deine Tätigkeit gefördert haben oder die das behindert haben? Also wie so Störfaktoren, sage ich jetzt mal?  Nurse: „Nein, ich habe/ Da fällt mir jetzt nichts ein, nee, eigentlich nicht.“ *Nurse1*  „Also irgendwie eher, dass ich denke, ich habe eh schon so viel zu tun und denke, ach nee, jetzt muss ich das auch. Obwohl ich denke, brauchen wir das jetzt oder braucht die Frau das jetzt überhaupt nochmal, so eine Stunde Gespräch mit mir. Aber so, also weißt du, so? Das eher so ein bisschen in der Motivation, dass ich dachte, irgendwie so.“ *Nurse2*  „Das ist eigentlich so die meiste Motivation dann auch für MoMS so, man wird ja quasi belohnt dafür, dass man wertgeschätzt wird sozusagen.“ *Nurse2* |
