# Supplementary material for: Motherhood choice in multiple sclerosis (MoMS) – Pilot trial of web-based decision support
Source: PLoS One. 2026 Jun 12;21(6):e0351108. doi: 10.1371/journal.pone.0351108 (PMC13262864; doi:10.1371/journal.pone.0351108)
Supplement: S9 Data — (PDF) [file pone.0351108.s009.pdf]

Study Protocol, Version 4.0 from 16.12.2021

MoMS

Motherhood choice in multiple sclerosis and systemic lupus erythematosus – a mixed methods study

**Title:**	Motherhood choice in multiple sclerosis and systemic lupus erythematosus – a mixed methods study
**Acronym:**	**MoMS**
**German Titel:**	Entscheidungsfindung zur Mutterschaft bei Multipler Sklerose und systemischem Lupus erythematodes - eine mixed-methods Studie
**Protocol Date:**	16.12.2021
**Study type:**	Mixed-methods study
**Principal Investigator:**	Prof. Dr. phil. Anne Rahn<br>Nursing Research Unit<br>Institute for Social Medicine and Epidemiology University of Lübeck<br>Ratzeburger Allee 160<br>23538 Lübeck<br>Germany<br>E-mail: a.rahn.ext@uke.de
**Co Investigator:**	Prof. Dr. C. Heesen<br>Institute of Neuroimmunology and Multiple Sclerosis<br>University Medical Centre Hamburg-Eppendorf<br>Martinistraße 52<br>20246 Hamburg<br>Germany<br>Tel.: 040-7410-54076<br>Fax: 040-7410-56973<br>E-mail: heesen@uke.de

Content

Content 2
Abbreviations 3
List of figures 4
List of tables 4
1 Protocol synopsis 5
2 Participating centres/investigators 7
3 Funding 7
4 Introduction 8
4.1 Background and study rationale 8
4.2 Aims of the study 10
4.3 Design 11
4.4 Study process 11
5 Research plan and methods 12
5.1 Systematic Reviews 12
5.2 Qualitative Interviews 13
5.3 Questionnaire development and translation 14
5.4 Web-based survey 16
5.5 Development of the decision support tool and the decision coaching programme 17
5.6 Qualitative feasibility testing of the decision support tool and the decision coaching programme 18
5.7 Randomised pilot 19
5.8 Multinational web-based survey 21
6 Data management 24
7 Ethics and dissemination 25
7.1 Consent or assent 25
7.2 Protocol amendment 25
7.3 Confidentiality 25
7.4 Data monitoring 25
7.5 Declaration of interests 25
7.6 Access to data 25
7.7 Dissemination policy 26
8 Authorship eligibility guidelines 26
9 Timetable and Milestones 27
10 References 30
11 Appendices 34
12 Signature Sheet 35

Abbreviations

CPS	Control Preference Scale
DA	Decision Aid
DCP	Decision Coaching Programme
DCS	Decisional Conflict Scale
DFG	Deutsche Forschungsgemeinschaft [German Research Foundation]
DMSG	Deutsche Multiple Sklerose Gesellschaft [German MS Society]
DST	Decision Support Tool
EBM	Evidence based medicine
EBPI	Evidence based patient information
FG	Focus Group
IPDAS	International Patient Decision Aid Standards
KKNMS	Kompetenznetz Multiple Sklerose [German Competence Network Multiple Sclerosis]
LKM	Leipziger Fragebogen zu Kinderwunschmotiven
MPWQ	Questionnaire on motherhood/ pregnancy choice and worries
MCKQ	Motherhood choice knowledge questionnaire
MCKQ-R	Motherhood choice knowledge questionnaire - revised
MS	Multiple Sclerosis
PRAQ-2	Pregnancy-Related Anxiety Questionnaire-Revised 2
PwMS	People with MS
RCT	Randomised Clinical Trial
SLE	Systemic Lupus Erythematosus
UKD	Universitätsklinikum Düsseldorf [University Medical Center Düsseldorf]
UKE	Universitätsklinikum Hamburg – Eppendorf [University Medical Center Hamburg Eppendorf]

List of figures

Figure 1- particular steps in both the development and feasibility testing phase. 11
Figure 2- Specifying the study steps with regard to the two different autoimmune disorders multiple sclerosis (MS) and systemic lupus erythematosus (SLE). EBPI= evidence based patient information. 12

List of tables

Table 1: Gant chart of “motherhood choice in SLE/MS – a mixed methods study”........**Fehler! Textmarke nicht definiert.**

1 Protocol synopsis

TITLE OF STUDY	Motherhood choice in multiple sclerosis and lupus erythematosus – a mixed methods study
OBJECTIVE(S)	1) To explore the knowledge as well as information needs regarding pregnancy among MS (multiple sclerosis) and SLE (systemic lupus erythematosus) patients and their physicians.<br>2) To develop and validate standardized tools to assess knowledge, concerns and decisional conflict regarding pregnancy among MS and SLE patients: translation and revision of the motherhood choice knowledge questionnaire (MCKQ, Prunty et al., 2008), development of a questionnaire on motherhood/pregnancy choice and worries (MPWQ) and conduction of web-based surveys.<br>3) Development of a web-based evidence-based patient information (EBPI) & decision aid (DA) and a decision coaching programme for women with MS considering pregnancy.<br>4) Feasibility testing of the decision support tool (EBPI + DA) and the decision coaching programme (EBPI + Coaching) for women with MS considering motherhood comprising qualitative feasibility testing and a randomised pilot study.
STUDY PROCESS	The study consists of 2 phases and 8 steps. Study steps 1 - 4 will address MS and SLE. The following study steps 5 - 8 will focus on MS. In phase 1, the development of the outcome measures and interventions will take place. Therefore, systematic reviews (MS & SLE) (step 1) and qualitative interview studies (step 2) will be conducted. Based on these, two novel questionnaires (MCKQ-R and MPWQ) will be developed and piloted in cross-sectional studies (step 3). The questionnaire MCKQ-R will be validated in a web-based surveys (step 4). Finally, a web-based tool for delivering evidence-based patient information (EBPI) will be developed. This will be extended by a decision aid for the decision support tool (DST) and a decision coaching programme (DCP) (step 5). In phase 2, the decision support tool and the decision coaching programme will be tested for feasibility. Thus, a qualitative feasibility testing will be carried out first (step 6). Afterwards, the DST and DCP will be tested for feasibility in a randomised pilot (step 7). The questionnaire MPWQ and MCKQ-R will be validated in a multinational web-based surveys for Czech Republic, Denmark, Germany, Italy, Ireland, Netherlands, Spain and United Kingdom (step 8).
RESULTS	1) Questionnaires to measure needs, concerns (MPWQ) and knowledge (MCKQ-R) of women with MS and SLE considering motherhood<br>2) A web-based decision support tool and a decision coaching programme to support women with MS in their decisional conflict of motherhood choice<br>3) Results on the feasibility of the evaluated intervention components.<br>4) Preliminary data on the potential of decision coaching including the EBPI or the decision support tool (MS) for further evaluation.
STUDY TYPE(S)	Mixed methods study
Participants	Women with MS/SLE (18-45 years) considering motherhood

STUDY DURATION	31 months
SUMMARY	Multiple sclerosis (MS) and Lupus erythematosus (SLE) are chronic diseases that particularly affect women between the ages of 18 and 45. Therefore, the issue of pregnancy is often very important for women with MS and SLE. However, misunderstandings, misinformation and uncertainties about MS/SLE and pregnancy are common among both patients and professionals. For this reason information provision and decision support is highly relevant and demanded for women with SLE and MS.<br>Systematic reviews and qualitative interviews will be conducted to explore the evidence base as well as needs, knowledge and concerns of women with MS/SLE considering motherhood. Further, questionnaires will be developed and validated to measure the patients' knowledge and concerns. Moreover, a web-based evidence-based patient information (EBPI) for women with MS considering motherhood will be developed. Building on this, a decision support tool and a decision coaching programme will be developed to support women with MS considering motherhood to decrease their decisional conflict. The programmes will be evaluated by a qualitative study and a randomised pilot for feasibility.<br>This mixed methods study could lay the foundation for future studies concerning women with chronic diseases considering motherhood.
PARTICIPATING CENTERS	• Institute of Neuroimmunology and Multiple Sclerosis, University Medical Center Hamburg-Eppendorf, Hamburg (Germany)<br>• Department of Neurology, St. Josef University Hospital, Bochum (Germany)<br>• Department of Rheumatology, University Hospital Düsseldorf (Germany)

2 Participating centres/investigators

Prof. Dr. phil. Anne Rahn **(principal investigator)**
Institute for Social Medicine and Epidemiology
University of Lübeck
Ratzeburger Allee 160
23538 Lübeck

Prof. Dr. med. Christoph Heesen
Institute of Neuroimmunology and Multiple Sclerosis
University Medical Centre Hamburg-Eppendorf
Martinistraße 52
20246 Hamburg

Julia Peper
Institute for Social Medicine and Epidemiology
University of Lübeck
Ratzeburger Allee 160
23538 Lübeck

Prof. Dr. Stefan Gold
Department of Psychiatry Campus Benjamin Franklin
Charité Universitätsmedizin Berlin
Hindenburgdamm 30
12203 Berlin

Cooperation partner:

PD Dr med. Kerstin Hellwig
St. Josef-Hospital
Gudrunstraße 56
44791 Bochum

Prof. Dr. U. Panzer
III. Medizinische Klinik und Poliklinik
Universitätsklinikum Hamburg Eppendorf
Martinistr. 52
D- 20246 Hamburg

PD Dr. med. Rebecca Fischer-Betz
Universitätsklinikum Düsseldorf
Moorenstr. 5
40225 Düsseldorf

3 Funding

Deutsche Forschungsgemeinschaft
Kennedyallee 40
53175 Bonn

4 Introduction

4.1 Background and study rationale

Autoimmune disorders represent a heterogeneous group of chronic diseases that are increasing in prevalence. In MS, about 2,300.000 people worldwide and around 220.000 in Germany are affected, whereas more than 30.000 people are affected by SLE in Germany (Brinks et al., 2014; Browne et al., 2014; Holstiege, Steffen, Goffrier, & Bätzing-Feigenbaum, 2017). Many autoimmune disorders disproportionately affect young women of child-bearing age. The sex ratio for multiple sclerosis (MS) is approximately 3:1 and rising, while the sex ratio in systemic lupus erythematosus (SLE) is up to 4:1 (Brinks et al., 2014; Holstiege et al., 2017) . For some of these diseases including MS, the rise in incidence over the past decades seems to increase especially in the female group of patients (Koch-Henriksen & Sorensen, 2010).
Women with autoimmune diseases such as MS, who are contemplating pregnancy often express concerns about whether they will pass the disease on to their babies, whether having a disease like MS or taking medications will harm the baby, and whether their disease will worsen leaving them unable to care for their babies (Payne & McPherson, 2010; M. Prunty, L. Sharpe, P. Butow, & G. Fulcher, 2008). Evidence from the last 20 years indicates that in general, pregnancy has neither a negative impact on the long-term disease course of mothers with MS nor their babies (Vukusic & Marignier, 2015). As disease activity is strongly ameliorated during pregnancy but may rebound postpartum, breastfeeding and return to immunotherapy after a delivery have to be discussed by the patients and their physicians (Finkelsztejn, Brooks, Paschoal, & Fragoso, 2011). In general, PwMS have been advised to discontinue treatment when they want to become pregnant. However, the advent of immunotherapies including biologicals have raised concerns about the safety of discontinuing the medication in the mother on one hand and of the risk of exposing the foetus on the other (Vukusic & Marignier, 2015).
In contrast, in women with SLE, a rare but often more severe form of autoimmunity, many issues arise. Women with SLE have a strongly increased risk of disease exacerbations and other complications including hypertension, nephritis or pre-eclampsia during pregnancy. These are all factors that can negatively influence the pregnancy and partly explain an augmented risk of foetal loss for women with SLE (Moroni & Ponticelli, 2016). While women with SLE have been advised not to become pregnant in the past, this attitude is changing enabling pregnancies supported by treating physicians (Lazzaroni et al., 2016).

Knowledge about the impact of pregnancies on autoimmune diseases is increasing as well as experiences with immunotherapies and their impact on mothers and unborn children in pregnancies. Experience has recently led to EULAR (European League Against Rheumatism) recommendations suggesting that pregnancy might be safe in SLE if certain safety precautions are taken (Andreoli et al., 2017). In MS several 100 of pregnancies have been reported with immunotherapies leading to elaborated risk estimates (Alroughani et al., 2016). Nevertheless, in clinical practice, uncertainties and lack of specific knowledge about pregnancy in MS and SLE are common both on the patient and even health professionals' side (i.e. family physicians, general neurologists, or obstetrician-gynaecologist) leading to a decreased realisation of motherhood (Clowse, Chakravarty, Costenbader, Chambers, & Michaud, 2012; Herndon, 2004; Thone, Thiel, Gold, & Hellwig, 2017). What women with MS or SLE know about having a baby and how this, along with their personal preferences and risk taking attitude, influences their family planning decisions, however, has not been thoroughly studied. As well health professionals' attitudes have not been investigated systematically.

Medical decision-making should be based on an exchange of information between at least two persons (e.g. patient and physician) as defined in the German "Patientenrechtegesetz" (No

author, 2013). Evidence-based patient information (EBPI) is a prerequisite to enable informed decisions. EBPI are based on a transparent methodological procedure by considering the current scientific evidence. They provide comprehensive, understandable, transparent, unbiased and objective information on health decisions. The benefit and harms of measures is based on patient-relevant outcome parameters such as mortality, morbidity and health-related quality of life (Lühnen J., 2017). Decisions aids aim to facilitate treatment decision-making in a shared decision-making process (SDM). They are based on EBPI and personal values and preferences are taken into account (Drug & Therapeutics, 2013). SDM is currently regarded as the ideal approach in medical decision-making based on the ethical principle of patient autonomy and on patient preferences (Stiggelbout, Pieterse, & De Haes, 2015). Here both, the physician and the patient, participate actively and responsibly in the decision-making process. The concept of decision coaching is a promising approach for beneficial and supportive encounters between patients and healthcare professionals, due to the potential of informed decision-making based on EBPI as well as SDM and a focus on patients' values (Stacey, Kryworuchko, et al., 2013; Stacey et al., 2008). It is non-directive approach, where a trained health care professional provides support in order to prepare a physician consultation (Stacey, Kryworuchko, et al., 2013).

Regarding informed decision-making in motherhood, there are only a few studies on information needs of women with MS considering motherhood. In one qualitative study on this issue, Prunty et al. (Prunty et al., 2008) reported substantially negative attitudes from health professionals as well as family members regarding family planning in women with MS, which adds to concerns of the women about their own and their child's health. Furthermore, many women felt time pressure. Payne et al. (Payne & McPherson, 2010) have shown that in contrast to other women, more people than just the partner, are involved in pregnancy choices in women with MS, especially physicians and women's mothers. However, it is not clear what women with MS know, how they perceive the different risks associated with pregnancy, and what risk they are willing to accept. Women with SLE tend to have a decreased number of pregnancies, the reasons for this includes concerns about the course of pregnancy, the risks for their babies and their care-giving capacities (Clowse et al., 2012).

As outlined above, decisions on forming or enlarging a family are challenging for women with MS/SLE. The information to be conveyed is complex, and it may be difficult to weigh up the risks and benefits of the different options available. People in Germany prefer a shared therapy decision making process (B. Braun & Marstedt, 2014) and people with MS (PwMS) explicitly demand active roles in the therapy decision-making process (Solari et al., 2013), as well as verified information. They make intensive use of the Internet (Colombo et al., 2014; Haase, Schultheiss, Kempcke, Thomas, & Ziemssen, 2012; L. Lorefice et al., 2013; Lorena Lorefice et al., 2013; Marrie, Salter, Tyry, Fox, & Cutter, 2013).

The implementation of decision aids in the field of motherhood choice as for example epilepsy and rheumatoid arthritis resulted in increased knowledge and even more important in lower decisional conflict (McGrath, Sharpe, Lah, & Parratt, 2017; Meade, Dowswell, Manolios, & Sharpe, 2015).

While there is no evaluated decision aid (DA) for women with SLE available, Prunty et al.(M. C. Prunty, L. Sharpe, P. Butow, & G. Fulcher, 2008) tested the efficacy of a motherhood choice decision aid in 194 women with MS in Australia showing that it significantly decreased decisional conflict and increased decision certainty, knowledge as well as self-efficacy. Although, development of the decision aid followed the CREDIBLE-criteria of the Ottawa decision framework (O'Connor et al., 2001), it did not meet the more recent criteria of evidence-based patient information (Bunge, Mühlhauser, & Steckelberg, 2010), the patient focused perspective of evidence-based medicine (EBM), and IPDAS (International Patient Decision Aids Standards

Collaboration, 2019). There is also a need for an update of evidence as the Australian decision aid was last updated in 2011 in Italy during a translation procedure. Further, some options are missing as for example the option to adopt a child as identified in focus group meetings in Italy (personal communication with A. Solari).

Greenhalgh et al. (Greenhalgh, Snow, Ryan, Rees, & Salisbury, 2015) highlighted important "biases" of evidence-based medicine against patients and carers calling for more focus on individual experiences and decision support networks than on patient physician encounters. While high quality information is at the crucial centre of any intervention to enhance patient participation in health-related decision-making, reflection about personal values, preferences and goals as well as the role of social support is highly relevant, particularly in pregnancy. Systematic reviews on shared decision-making interventions show that decision coaching can be an important factor to enhance patient autonomy (Stacey, Kryworuchko, et al., 2013; Stacey et al., 2014). In combination with decision aids, decision coaching can increase participation in decision-making processes, reduce costs, and lead to intervention-specific positive outcomes compared to decision aids alone (Stacey, Macartney, Carley, Harrison, & Costars, 2013). Results are in particularly promising when nurses offer the coaching. True balancing of the information is crucial (Abhyankar et al., 2013) and especially relevant in the sensitive area of motherhood choice. In fact, the non-directive provision of support is at the core of the decision coaching concept as described by Stacey (Stacey, Kryworuchko, et al., 2013). Given the medical and psychological complexity of the issue, women with MS or SLE, in Germany might benefit from decision coaching on motherhood choice in addition to a decision aid.

In summary, this project aims to explore the clinical needs, knowledge gaps, and decisional conflicts with regard to pregnancy and motherhood of young women with autoimmune disorders. We will study two paradigmatic disorders: MS and SLE, the two most common autoimmune diseases in young women. MS and SLE share general aspects of immunotherapy and genetic risk associated with autoimmunity, but have distinct characteristics with regard to disease activity during pregnancy. This comparison will therefore be highly informative regarding clinical and psychological issues related to pregnancy in autoimmunity. In this respect, the development and evaluation of decision support programmes on motherhood choice in SLE and MS will serve as a blueprint for future developments of similar interventions in chronic diseases.

4.2 Aims of the study

The first specific aim of this project is to explore the knowledge stage as well as the attitudes, needs and anxieties towards pregnancy among MS and SLE patients and their physicians or healthcare employees. Therefore, two systematic reviews (one for MS and one for SLE) will be conducted in this area. In the next step, a new questionnaire will be developed (questionnaire on motherhood/ pregnancy choice and worries, MPWQ). A second questionnaire addressing knowledge will be translated and revised (motherhood choice knowledge questionnaire – revised, MCKQ-R). Both questionnaires will be tested for feasibility and validity in cross-sectional studies.

The further steps of the intervention will focus exclusively on MS.

The following feasibility testing and the pilot of the DST and the DCP will give an idea whether the decision coaching programme on motherhood choice or simply providing a decision aid is best suited for decision-making. We hypothesise that the decision aid plus coaching will lead to more positive (e.g. lower decisional conflict, (Buchholz, 2011)) outcomes than the decision aid alone. But we also recognise potential barriers as for example that the coaching is provided by an external nurse and that the coaching will require new resources.

4.3 Design

A multiphase mixed methods study (Creswell & Plano Clark, 2011) will be conducted to develop and evaluate the multicomponent programme. Qualitative and quantitative methods will be used intentionally to acquire a comprehensive impression of study processes. The study conduct will be based on the MRC framework for developing and evaluating complex interventions (Craig et al., 2008; Moore et al., 2015) for a step-wise approach. In phase 1, we will stepwise develop the DST and DCP. For this reason, we will conduct systematic reviews on particular topics (see 5.1) as well as qualitative interviews. Further, we will develop, adapt and validate questionnaires. In phase 2, the feasibility testing of the developed decision support tool and the decision coaching programme will follow (Arain, Campbell, Cooper, & Lancaster, 2010; Eldridge et al., 2016) including a qualitative feasibility testing as well as a randomised pilot.

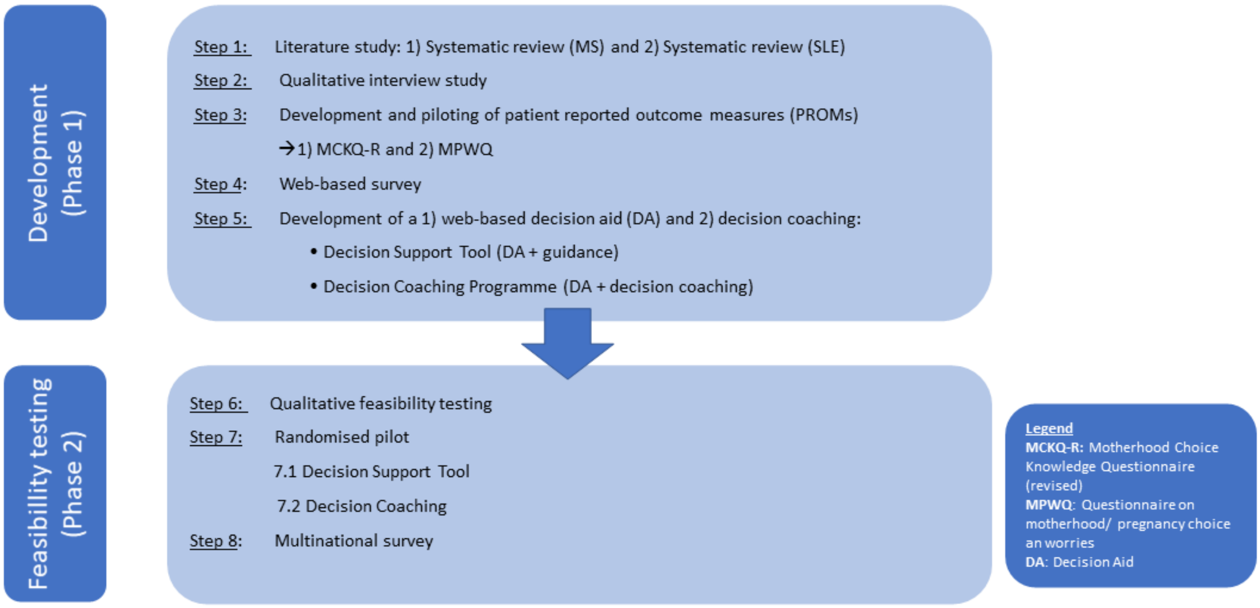

Figure 1- particular steps in both the development and feasibility testing phase.

The project will be based on the UK MRC frameworks for developing and evaluating complex interventions (Craig et al. 2008; Moore et al. 2015) (phase 1 and 2).
The “Throughput-Model” lays the foundation for a continuous optimisation of care by considering the complexity of interventions and context factors (input) as well as the observation process (throughput) when defining intervention outcomes (Pfaff et al., 2017b). This project mainly focusses on input by the development of a complex intervention. The intervention addresses patients, but also nurses are involved as they are trained to deliver the intervention. The context factors are specifically addressed by an accompanying process evaluation (Graham F. Moore et al., 2015). Throughput will be addressed by qualitative and quantitative measures as described under specific aim 2. Outcomes will be tested and further defined by the feasibility study. Further, the feasibility phase will help to explore the output and based on the analysis, the input may be modified within a future randomised clinical trial (RCT).

4.4 Study process

The study consists of 2 phases and 8 steps. Study steps 1 - 4 will address MS and SLE. The following study steps 5 – 8 will focus on MS (see Figure 1). Therefore, the project stops for SLE after the web-based survey.

5 Research plan and methods

Overall, the project is guided by the principles evidence-based medicine (Sackett, Rosenberg, Gray, Haynes, & Richardson, 1996) and evidence-based health information (Lühnen J., 2017) as well as the concepts of SDM (Elwyn et al., 2001a) and decision coaching (Stacey, Kryworuchko, et al., 2013). The "Theory of Planned Behaviour" (Ajzen, 2011), which was already used in earlier studies, forms the overall theoretical basis concerning behavioural psychology.

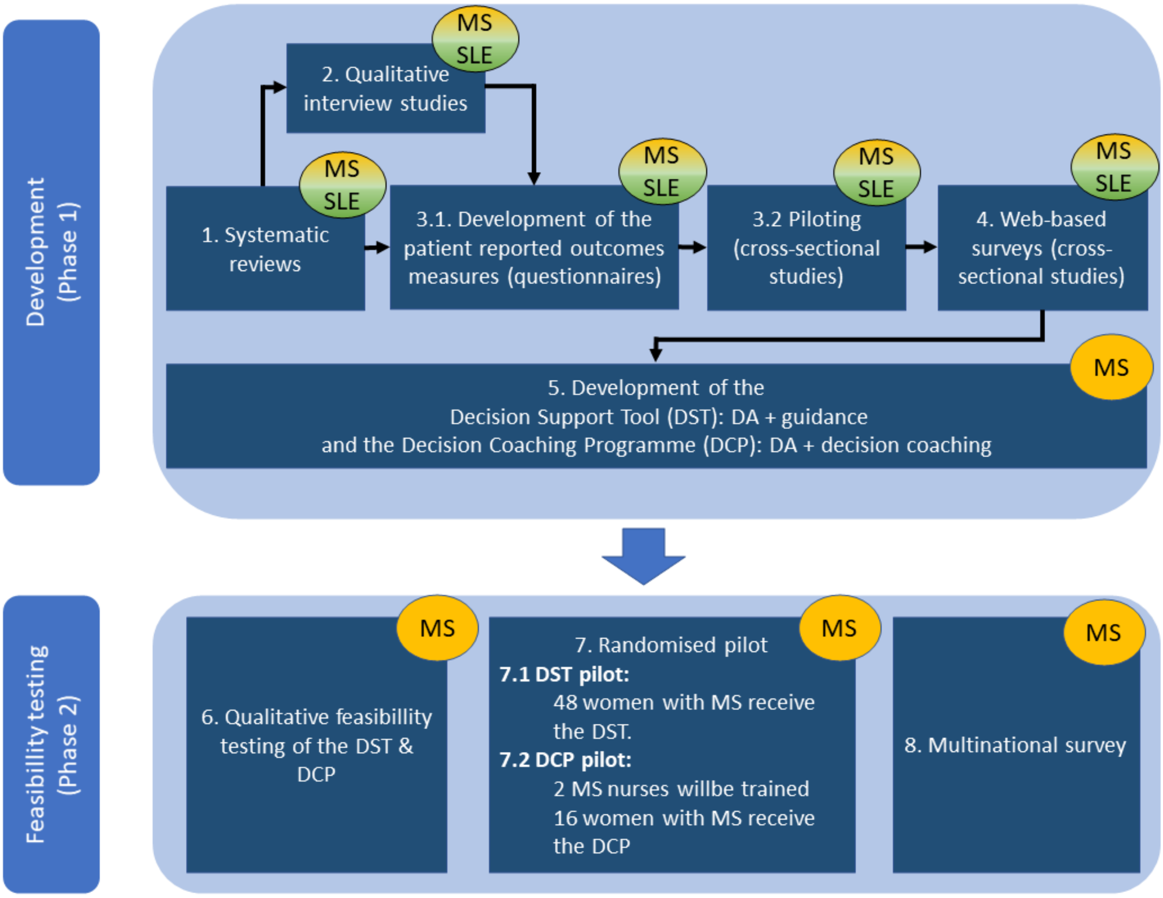

Figure 2- Specifying the study steps concerning the two different autoimmune disorders multiple sclerosis (MS) and systemic lupus erythematosus (SLE). DA= Decision Aid.

In phase 1 - development of the outcome measures and the intervention programmes will take place (steps 1-5) and in phase 2 the feasibility of the decision support tool and the decision coaching programme will be tested (steps 6-7, figure 2).

5.1 <u>Systematic Reviews</u>

5.1.1 Procedure

As a first step, systematic literature reviews on MS and SLE will be conducted to explore all available qualitative and quantitative data on patients' knowledge and attitude towards pregnancy as well as their needs on family planning. This includes various issues like the influence of a patient's pregnancy to the course of MS/SLE, the meaning of the social environment and also the aspect of career. Additionally, the evidence on immunotherapies in particular with

regard to (a future) pregnancy and lactation period in women with MS/SLE will be reviewed. Nevertheless, the effect of fertility treatment, e.g. regarding the course of MS/SLE, will be explored.

Therefore, literature searches will be performed in MEDLINE (via Ovid) according to defined questions using PICO and variants as well as defined study selection criteria. Two reviewers will screen the findings (title/abstract), identified full-texts and perform a risk of bias evaluation of included studies. Subsequently, the data extraction of the identified studies will follow by one researcher. Another researcher will control the extracted data. Endnote and Rayyan will be used for bibliographic management and screening.

5.1.2 Expected results

The systematic reviews of the relevant literature will provide the evidence base for the patient interview and questionnaire development outlined below.

5.2 <u>Qualitative Interviews</u>

5.2.1 Study setting

Focus groups (FG) with women with MS/SLE will be conducted by two researchers (JP and AR) in different centres (e.g. Bochum and Hamburg for MS and Düsseldorf and Hamburg for SLE. In addition, personal in-depths interviews with patients, patient representatives, neurologists and rheumatologists will be performed by telephone.

5.2.2 Participants

The focus groups and the personal interviews will be conducted with women with MS and SLE aged 18-45 years considering motherhood, being pregnant, or parenting. Only persons, who are fluent in German and have provided signed informed consent will be included.

Neurologists and rheumatologists will be eligible to participate when they have a special expertise (MS or SLE) and have provided signed informed consent.

5.2.3 Procedure

The structured interview guidelines will be developed based on the results from the literature searches and other materials as for example the Australian decision aid on motherhood choice (M. Prunty et al., 2008). One interview guide will address experiences, counselling and information needs as well as concerns of women with SLE/MS to be used in focus groups and personal interviews and the other one will address physician's perception regarding motherhood in MS/SLE.

Before starting the focus group and the personal interviews, the participants have to provide informed consent and to fill a short questionnaire collecting demographic data and some specific information. Each interview will be audio-recorded and transcribed verbatim.

5.2.4 Expected results

The analysis will provide important information on information needs, experiences and concerns of women with MS/ SLE and the perception of experts on the topic. The thematic analysis will lead to the development/ revision of the questionnaires and will provide important information for the development of the decision support tool and the decision coaching programme.

5.2.5 Sample Size

Each focus group meeting will consist of 5-10 participants plus two moderators. It is planned to have two focus groups with women with MS and two with women with SLE. The personal interviews will be conducted with women with MS (n=5) and SLE (n=5), with neurologists (n=3) and rheumatologists (n=3).

5.2.6 Recruitment

Participants for the focus group meetings and the interviews will be recruited from the MS clinic at the University Medical Center Hamburg Eppendorf (UKE, Universitätsklinikum Hamburg-Eppendorf), the MS pregnancies registry at the Joseph Hospital in Bochum (MS) and the SLE registry of the University Medical Center Düsseldorf (UKD, Universitätsklinikum Düsseldorf) (SLE). The physicians will be recruited throughout Germany.

5.2.7 Participant timeline

The focus groups interviews will take about 90 minutes. The personal interviews will be scheduled for 30-60 mins.

5.2.8 Data analysis and statistical methods

A qualitative thematic content analysis (V. Braun & Clarke, 2014) using deductive and inductive categories will be performed by JP. The phases of the thematic analysis include: 1) familiarisation with the data, 2) generalising initial codes, 2) searching for themes, 4) reviewing themes, 5) defining and naming themes and 6) producing the report (V. Braun & Clarke, 2014). A computer-assisted qualitative data analysis software (MAXQDA 2018) will be used to manage and organise the data.

5.3 Questionnaire development and translation

5.3.1 Study setting

Two cross-sectional studies will be performed with women from the MS pregnancies registry in Bochum (PD Dr. Kerstin Hellwig) and SLE registry in Düsseldorf (Prof. Dr. Fischer-Beetz) to test the developed questionnaire and gather further information on information needs. The conduction of the studies will be web-based.

5.3.2 Participants

The cross-sectional studies will include women (18– 45 years) with MS/SLE considering pregnancy. Only persons, who state that they are fluent in German and who have provided signed informed consent will be included.

5.3.3 Procedure

The motherhood choice knowledge questionnaire (M. Prunty et al., 2008) will be revised and translated (MCKQ-R) based on the literature reviews and interviews. In addition, based on the qualitative data obtained in the interviews and the results of the literature reviews, a questionnaire on motherhood/pregnancy choice and worries (MPWQ) will be developed. Both questionnaires will be qualitatively evaluated with think-aloud (Jaspers, Steen, van den Bos, & Geenen, 2004) and teach back methodology and refined. Afterwards, the questionnaire will be piloted in cross-sectional studies to test basic psychometric properties. The studies will be performed using UNIPARK (UNIPARK & QuestBack, 2019), a widely established web-based

survey methodology and will be conducted according to appropriate reporting (Eysenbach, 2004).

5.3.4 Expected results

The analysis of the cross-sectional studies will lead to a final version of two different questionnaires for women with MS/SLE considering motherhood. The first one is the motherhood choice knowledge questionnaire (**MCKQ-R**) MS/SLE and the second the motherhood/pregnancy choice and worries questionnaire (**MPWQ**). Further, additional questions on information needs of women with SLE/ MS will be used to plan intervention programmes according to the needs of women with MS/ SLE.

5.3.5 Sample Size

Both cross-sectional studies will include about 100 participants.

5.3.6 Recruitment

Participants will be recruited via the MS pregnancies registry Joseph Hospital in Bochum (MS) and the SLE registry of the UKD Dusseldorf (SLE) via mailing lists. Informed content and the cross-sectional survey will be conducted online via UNIPARK.

5.3.7 Participant timeline

The mean duration of the participation in the cross-sectional study will take about 45 minutes.

5.3.8 Data analysis and statistical methods

The raw data of the cross-sectional studies will be exported from the online database UNIPARK. Data analysis will be performed with the software programme SPSS (version 24/25) and demographic data will be analysed using descriptive statistics.

The newly developed questionnaires will be evaluated for their psychometric properties using standard procedures as previously described (Gold et al., MSJ 2001) and modified if necessary. This includes assessments of item difficulty and score distributions (floor/ceiling effects), reliability (internal consistency) and aspects of validity (construct validity, convergent and discriminatory validity). An item analysis is planned to determine the item difficulty, item discriminatory power and item distribution. Values between 0.20 and 0.80 are expected for the item difficulty, and the item discriminatory power should be between 0.40 and 0.70. For the determination of the internal consistency of the questionnaire, Cronbach's alpha will be calculated; hereby, for a high internal consistency of the test, the values should be above 0.70. Reproducibility over time will be assessed by Pearson product moment and intraclass correlations (ICC). Appropriateness of the questionnaires will be tested by examining score distributions and floor/ceiling percentages on each scale. In general, a percentage of less than 20% is considered satisfactory. Construct validity will be assessed by exploratory factor analysis (Bühner, 2006).

5.4 Web-based survey

5.4.1 Study setting

Two web-based surveys will be conducted across Germany in cooperation with patient organisations the German Multiple Sclerosis Society (DMSG, Deutsche Multiple Sklerose Gesellschaft) and Lupus Selbsthilfe e.V.), who will inform about the survey on their websites. The studies will be performed using UNIPARK.

5.4.2 Participants

The participants will be women with MS/SLE considering pregnancy at child-bearing age between 18 and 45 years. Available internet access is mandatory. Only persons, who state that they are fluent in German and who have provided signed informed consent will be included.

5.4.3 Procedure

These web-based surveys including the MCKQ-R and the MPWQ will clarify worries and concerns but also the knowledge of women with MS and SLE regarding pregnancy. The survey will be conducted using UNIPARK and will consist of a demographical and disease specific section, followed by the MCKQ-R, the MPWQ and the Decisional Conflict Scale (DCS) (Buchholz, 2011). In addition, a slightly modified version of the Control Preference Scale (CPS) (Degner, Sloan, & Venkatesh 1997), the Pregnancy-Related Anxiety Questionnaire-Revised 2 (PRAQ-2) (Mudra et al. 2019) and the extension of the Leipzig Questionnaire of Motives to have a Child (LKM) (Ernst, Brähler, Wild, Faber, Merzenich, & Beutel 2020) will be included. Degner, L. F., Sloan, J. A., & Venkatesh, P. (1997). The control preferences scale. Canadian Journal of Nursing Research Archive, 29(3).

5.4.4 Expected results

The web-based survey will on the one hand clinically validate the MCKQ-R and the MPWQ and on the other hand gathering the percentage of women with MS and SLE considering pregnancy as well as their worries, concerns, knowledge and decisional conflict (explored with the MCKQ-R, DCS, and the MPWQ). Therefore, patient reported outcome measurements for an intervention on elaborating motherhood choice in MS and SL are available after the analysis.

5.4.5 Sample Size

Each web-based survey will include at least 100 women with MS/SLE at the childbearing age (18-45 years) considering pregnancy.

5.4.6 Recruitment

The participants recruited via the websites of the DMSG and Lupus Selbsthilfe e.V. In addition, centres and local self-help organisations will be asked to inform potential participants via mailing lists.

5.4.7 Participant timeline

The mean duration of the participation in the web-based survey will take about 45 minutes.

5.4.8 Data analysis and statistical methods

See 5.3.8 (Questionnaire development and translation: data analysis and statistical methods).

5.5 Development of the decision support tool and the decision coaching programme

In this part, the development and evaluation of the tools will be exclusively on MS. However, the methodological approach would be suitable for SLE and could be carried out in a subsequent project with additional funding

The **decision support tool** (DST) will consist of a web-based evidence-based patient information platform and a decision aid. Whereas the **decision coaching programme (DCP)** will be based on the web-based patient information platform and a decision coaching programme.

The web-based evidence-based patient information, the decision aid and the coaching programme (training for nurses and the coaching intervention) will be developed based on the steps described above (literature reviews, qualitative interviews, web-based surveys) and previous work (online information platform on immunotherapies (DECIMS-Wiki) and coaching programme (Rahn et al., 2018). The programmes will be developed based on the principles of EBPI (Lühnen J., 2017) and the knowledge transfer will reflect educational concepts (Roth, 1971). Experts and expert patients will be involved in the development.

Decision support tool

A web-based evidence-based information platform, the DECIMS-Wiki (DECIMS, decision coaching in multiple sclerosis, http://wiki2.kkn-ms.de/), on multiple sclerosis focusing immunotherapy options was developed by our group (Rahn et al., 2018). The DECIMS-Wiki is hosted by the German Competence Network Multiple Sclerosis (KKNMS). The decision support tool on motherhood choice will be added to the DECIMS-Wiki as an additional chapter. Therefore, the whole DECIMS-Wiki will be copied and renamed for the feasibility phase and the new chapter will be added. Only the participants of this study will gain access.

The following topics will be addressed by the EBPI (considering results from preliminary work including the systematic review to identify the evidence base):

- Risks of exacerbations and progression of MS due to pregnancy, childbed and lactation (short and long-term)
- Genetic MS risk for children of women and men with MS
- Meaning of MS for the course of pregnancy and childbirth
- Risks of immunotherapies for the unborn (pregnancy) and new-born (lactation)
- Risk of fertility treatment for the course of MS
- Meaning of the social environment and impact on career of motherhood with MS

The online EBPI will be complemented by a decision aid to facilitate individual preference-based decision-making for the decision support tool group. The decision aid will be printable to allow the participants to share the information with others as for example physicians and relatives.

Decision coaching programme

Training course
The curriculum for the training course for decision coaches will follow the train-the trainer principle, the knowledge transfer will be guided by established educational theories (Roth, 1971) and concepts and will include six SDM steps following a previous developed immunotherapy decision coaching curriculum (Rahn et al., 2018). It will specifically address the evidence, the

use of the decision support tool and the workbook on motherhood choice. Further, role plays with case examples and simulated patients will be performed to practice decision coaching on motherhood choice using supporting materials.

Decision coaching
The coaching will be provided by phone or via a web conference. There will be one decision coaching session up to 1.5 hours per women with MS. The decision coaching will be guided by a workbook on motherhood choice following six SDM steps (Rahn et al., 2018) comprising 1) reviewing the problem, (2) key message, (3) information about pros and cons of each option, (4) expectations of the patient, (5) decision, and (6) arrangements (Elwyn et al., 2001b). Further, the online EBPI will be used during the decision coaching.

The specific aim is the development of the decision coaching programme and the decision support tool. The web-based EBPI will be developed for both inventories (DST and DCP). In addition, a particular decision aid will be created for the DST, whereas a specific workbook on motherhood choice will be designed for guiding the DCP.

5.6 Qualitative feasibility testing of the decision support tool and the decision coaching programme

5.6.1 Study setting

The feasibility testing of the DST will be proceeded by phone or web conference. The training course for the decision coaches will take place in the UKE in Hamburg. In case the infection situation of the Covid-19 pandemic does not allow an in-person training course, the training will be conducted online.

5.6.2 Participants

Selected nurses and neurologists with a MS background will be included. Additionally, expert patients and women with MS considering motherhood will participate. Only persons, who have provided signed informed consent will be included.

5.6.3 Procedure

First of all, the decision support tool and the coaching programme will first be presented selected women with MS, physicians and nurses. During this phase, the programme will be continuously developed based on the feedback using qualitative methods (think aloud, teach-back) (Buber, 2009; DeWalt et al., 2011). The feasibility testing (Bowen et al., 2009) is considered as a test of practicability (e.g. concerning content, graphical presentations, clarity, readability) and acceptance (e.g. concerning illustrations, adequacy of examples, understandability) of the content of the coaching programme and the decision support tool. The interviews will be conducted via Cisco Systems' online meeting software WebEx, provided by the UKE, or telephone. The selected women with MS will be interviewed in focus groups. All interviews will be recorded. During that phase, it will be figured out if the coaching should be delivered by phone or web conference. While telephone coaching can be conducted easily, a web conference would have the benefits of allowing participants to see each other and work together through a possible split of the screen.

5.6.4 Expected results

The specific aim of the qualitative feasibility testing is considered as a continuous revise as well as a test of practicability and acceptance of the coaching programme and the decision support tool.

5.6.5 Sample Size

For the feasibility testing of the DCP and the DST, nurses (n=4), physicians (n=4), expert patients (n=2) and women with MS (n=10) will be included.

5.6.6 Recruitment

The participants for the qualitative feasibility testing will be recruited in Hamburg, Bochum, Kiel and Bonn as well as via mailing lists.

5.6.7 Participant timeline

In the qualitative feasibility testing, the participants (expert patients, PwMS, nurses and physicians) will be interviewed. Each interview will take around 45 – 90 minutes.

5.6.8 Data analysis and statistical methods

Analysis of the qualitative data obtained during the feasibility phase will be conducted after the transcription of the interviews according to thematic content analysis (V. Braun & Clarke, 2014). For details of the thematic analysis see Data analysis and statistical methods5.2.8.

5.7 Randomised pilot

5.7.1 Study setting

Two MS nurses from two different university centres (Hamburg and Bochum) will be trained for the DCP There will be two groups; one group receives the decision support tool and the other group the decision coaching programme. The participants will be recruited across Germany.

Reminders are sent via a neutral email address, which is not assigned to the project. The compilation of the declaration of consent (including voluntariness) and the handling of all data collected in the context of the study are carried out according to the recommendations of the Hamburg Chamber of Physicians (Hamburg, 2016).

5.7.2 Participants

Women, aged 18 years or older with suspected (Miller, Chard, & Ciccarelli, 2012) or relapsing-remitting MS (Thompson et al., 2018) considering motherhood are eligible. Women cannot participate when they have no Internet access or information uptake is affected by severe cognitive deficit. In addition, two MS nurses will be trained for the DCP. All participants have to give informed consent in advance.

5.7.3 Procedure

After providing informed consent and filling in the baseline questionnaires (see 5.2.3), a study nurse of the MS outpatient clinic of the University Medical Center Hamburg-Eppendorf will

randomise eligible study participants into the two groups (1:3 allocation ratio) using block randomisation. After successful randomisation, the study nurse will provide the access to the information platform and the DST or DCP to the participating women. The participating women will be blinded as to whether they belong to the DST or DCP group.

Decision support tool: women with MS will receive the decision support tool. They will have access to the tool for two weeks and will be asked to fill in questionnaires after that period. All questionnaires will be sent by post with a stamped addressed envelope.

Decision coaching programme: 2 experienced MS nurses will be trained in the developed motherhood choice decision coaching programme. Afterwards, each nurse will provide the coaching programme for women with MS. It is intended that women will have one coaching session within two weeks after they gained access to the information platform. The coaching sessions will be audiotaped by the nurse. The women with MS will be asked to fill in questionnaires again after the decision coaching session. All questionnaires will be sent by post with a stamped addressed envelope.

At the beginning of their participation in the study, women with MS in both groups will be encouraged to make an appointment with their neurologist to discuss motherhood choice after the intervention.

After they have finished the intervention, at least 10 participants from each group will be interviewed by phone using a semi-structured interview guide addressing the feasibility. It will be addressed as well whether the topic was discussed with a neurologist. The nurses will be interviewed after they have provided the last coaching session at the end of the study. The semi-structured interviews will focus on the feasibility of the whole intervention. The randomised pilot will be conducted according to the CONSORT extension, a guidance for pilots and feasibility trials, which may be followed by a future RCT (Thabane et al., 2016).

5.7.4 Expected results

The following outcomes will be measured in both groups. Decisional conflict (Buchholz 2011) was the primary outcome measure in the study of Prunty et al (M. C. Prunty et al., 2008) on motherhood choice in women with MS and will be applied as a key outcome measure in this project to explore possible effects. Further, knowledge (MCKQ-R) (M. C. Prunty et al., 2008) and attitude and worries towards pregnancy will be assessed (MPWQ). A process evaluation questionnaire addressing the feasibility of the intervention will be developed and women will be asked to fill in after the corresponding intervention. Here, also possible unintended consequences like anxiety towards immunotherapies are addressed. In addition, a slightly modified version of the Control Preference Scale (CPS) (Degner et al., 1997) will be included to assess the women's preferences for involvement in decisions about immunotherapies when they want to have a child.

Further, after the coaching sessions, the exploratory ratings of the extent of SDM (explored with MAPPIN'SDM) (Kasper, Hoffmann, Heesen, Köpke, & Geiger, 2012) will be conducted based on an audio-analysis. MAPPIN'SDM enables to calculate convergent validities between assessments from different perspectives (here e.g. patient, MS nurse and reviewer). Additionally, perceived SDM will be addressed in both groups by questionnaires.

5.7.5 Sample Size

For the decision support tool pilot, 48 patients will be recruited.

For the decision coaching programme, two groups will be formed, each group with n=1 nurse and n=8 women with MS, so in total n=2 nurses and n=16 women with MS.

5.7.6 Recruitment

Participants will be recruited online via MS groups and self-help organizations.

5.7.7 Participant timeline

The participant in both groups will be able to use the information platform for two weeks and receive questionnaires afterwards.

Additionally, participants in the coaching group will receive a decision coaching session by a trained MS nurse with a duration of maximal 1.5 hours within 10-14 days after the received access to the information platform. After the decision coaching session, participants will be asked to fill in some questionnaires (see above).

After they have finished the intervention, at least 10 participants from each group.l,, will be interviewed by phone using a semi-structured interview guide addressing the feasibility within four weeks. It will be addressed as well if the topic was discussed with a neurologist. The nurses will be interviewed after they have provided the last coaching session at the end of the study. The semi-structured interviews will focus on the feasibility of the whole intervention.

5.7.8 Data analysis and statistical methods

The analysis of the data obtained in the randomised pilot will be performed primarily descriptively. Data will be summarised and displayed visually when appropriate. For exploration, a t-test for independent samples is used to assess whether there are differences in decisional conflict, knowledge (MCKQ) and attitude/worries towards pregnancy (MPWQ) between the two groups. The analysis of the recorded decision coaching sessions will be performed based on MAPPIN'SDM in order to assess the involvement of women with MS in the coaching session (Kasper et al., 2012).

Qualitative and quantitative data obtained in the randomised pilot phase will be analysed separately as described. For the description of the thematic analysis of the qualitative data see Data analysis and statistical methods 5.2.8. Afterwards, the data will be merged using a joint display table and discussed (Creswell & Plano Clark, 2011).

5.8 <u>Multinational web-based survey</u>

5.8.1 Study setting

The multinational web-based survey will be conducted across different countries in cooperation with local self-help organisations. For instance, in Germany the self-help organisation ‘Deutsche Multiple Sklerose Gesellschaft’ (DMSG) is involved and will inform about the survey on their website. Previously, a qualitative evaluation with think-aloud will be conducted. In Germany, the qualitative evaluation will take place in the MS outpatient clinic of the University Medical Center Hamburg-Eppendorf. It is planned that the following countries will participate in the web-based study: Czech Republic, Denmark, Germany, Italy, Ireland, Netherlands, Spain and United Kingdom. The multinational survey will be performed using LimeSurvey (LimeSurvey GmbH, 2021), a widely established web-based survey tool.

5.8.2 Participants

The participants for the survey and qualitative evaluation with think-aloud will be women with multiple sclerosis (MS) at child-bearing age between 18 and 45 years. Women who either have

already at least one child or who are dealing or have dealt with the issue motherhood are eligible to participate. Available internet access is mandatory for the web-based survey. Only women who indicate that they are fluent in the local national language and who have provided signed informed consent will be included.

5.8.3 Procedure

The qualitative evaluation with think-aloud and the web-based survey will include two questionnaires. The questionnaire on motherhood/pregnancy choice and worries (MPWQ) is designed to measure concerns, attitudes and coping strategies regarding pregnancy and motherhood. The motherhood choice knowledge questionnaire (MCKQ-R) will clarify the knowledge on MS and pregnancy. Both questionnaires were piloted in a cross-sectional study conducted primarily with women from a cohort exploring the modulation of immune function during pregnancy in MS (see 5.3 Questionnaire development and translation). The final results of piloting are currently being analysed. After this analysis, the final number of scales of the MPWQ (concerns, attitudes and/or coping strategies) will be identified. The validation of both questionnaires will be performed across Germany in cooperation with the DMSG before the multinational survey starts (see protocol 5.4 Web-based survey on page 17).

The web-based survey will consist of a demographical and disease specific section, a modified version of the Control Preference Scale (CPS) (Degner et al., 1997), followed by the MPWQ and MCKQ-R.

For the assessment of the convergent validity, the web-based survey will be included the validated Decisional Conflict Scale (DCS) (Buchholz et al. 2011) and Hospital Anxiety and Depression Scale (HADS) (Zigmond & Snaith, 1983) (See 5.3.8 Multinational web-based survey: Data analysis and statistical methods). In addition, the Pregnancy-Related Anxiety Questionnaire-Revised 2 (PRAQ-2) (Mudra et al. 2019) and the extension of the Leipzig Questionnaire of Motives to have a Child (LKM) (Ernst et al. 2020) will be included. All electronic data from the web-based surveys will be collected anonymously.

Before the multinational survey will be start, all questionnaires that are not yet existing in the right language of the local country will be translated forwards and backwards. Afterwards, they will be qualitatively evaluated with women with MS using think-aloud (Jaspers et al. 2004). Women with MS are given the two developed questionnaires (MPWQ and MCKQ-R) as well as all further translated questionnaires and asked to fill them out aloud. Thus, the the comprehensibility and user-friendliness of thes questionnaires will be assessed.

5.8.4 Expected results

The web-based survey will on the one hand validate the MCKQ-R and the MPWQ for the participating countries. On the other hand, the survey will be gathering the percentage of women with MS considering pregnancy as well as their worries, attitude, knowledge and decisional conflict across the different countries (explored with the MCKQ-R, DCS, and the MPWQ). Therefore, cross-country insights and differences about motherhood and MS can be identified.

Based on the results of an international study on the risk knowledge level in people with relapsing-remitting MS, we expect that the knowledge level about MS and motherhood/pregnancy will be moderate in all participating countries. Generally, we hypothesise that a higher knowledge level is associated with women who are currently considering a pregnancy or are already pregnant compared to women who have already children, because the issue of motherhood and MS is more present in these women.

Regarding the results of the MPWQ, we expect different results in each country for the possible scales attitudes, concerns as well as coping strategies. Depending on the country, there might be participants who will be more concerned or have more coping strategies in relation to participants from the other countries. In general, we assume that participants who have already had at least one child after the MS diagnosis will be less concerned, have more coping strategies and a more positive attitude towards MS and motherhood compared to childless women or women considering pregnancy.

5.8.5 Sample Size

In each country, the qualitative evaluation will include and least five and the web-based survey at least 50 women with MS at the childbearing age (18-45 years) considering pregnancy in each country.

5.8.6 Recruitment

The participants will be primally recruited via local self-help organisations of the different countries. Here, flyers, websites, social media sites of the organisations as well as mailing lists will be used to inform potential participants. In addition, MS outpatient clinics will recruit participants for the qualitative evaluation and will inform about the survey.

5.8.7 Participant timeline

The mean duration of the participation in the qualitative evaluation with think-aloud will take about 20 minutes and in the web-based survey about 30 minutes.

5.8.8 Data analysis and statistical methods

The audio material of qualitative evaluation will be transcribed if necessary. Each participating country will evaluate its qualitative data descriptively. The pseudonymized results of the qualitative evaluation of each country will be collected in Hamburg and evaluated.

The raw data of the multinational study will be exported from the online database LimeSurvey. Data analysis will be performed with the software programme SPSS (version 24/25) and demographic data will be analysed using descriptive statistics.

The developed questionnaires (MCKQ and MPWQ) will be evaluated for their psychometric properties using standard procedures as previously described (Gold et al., MSJ 2001) and modified if necessary (see 5.3.8 Questionnaire development and translation: data analysis and statistical methods). This includes assessments of item difficulty and score distributions (floor/ceiling effects), reliability (internal consistency) and aspects of validity (construct validity and convergent validity). A detailed description can be found in the protocol (see 5.3.8 Questionnaire development and translation: data analysis and statistical methods).

Differences in MCKQ and MPWQ scores across countries will be calculated using ANOVA. A mixed model regression approach will be used to investigate the effect of independent variables, such as the score of the Patient Determined Disease Steps (PDDS) scale, age, number of children before MS diagnosis and being on disease modifying drugs, on the MCKQ and MPWQ scores.

6 Data management

All data gained within the study will be assessed anonymously or will be pseudonymised and there will be no possibility to link data to persons without access to the code list.

The recorded interviews will be deleted after transcription and will be archived in anonymised and transcribed form. All electronic data from the web-based surveys will be collected anonymously via the secure online platform UNIPARK or LimeSurvey.

Survey data, collected by the webtool LimeSurvey, will be stored on a server of the LimeSurvey GmbH in Germany. LimeSurvey GmbH claims to be compliant with the General Data Protection Regulation (GDPR; Datenschutzgrundverordnung) (LimeSurvey GmbH, 2021). An agreement on commissioned processing in accordance with Art. 28 GDPR is in preparation. Only authorised persons will have access to the survey data.

Access to the web-based EBPI will be pseudonymised, but the email-address is saved by the system. Through the personal access of patients to the decision support tool based on a personal account (sent via email), it will be possible to individually track the use of the tool (e.g. frequency, use of different parts), which will be used for evaluation. This will be performed with Piwik, an encapsulated plugin to the Joomla platform, where the system is hosted by the KKNMS. Therefore, the user IP addresses will be stored by the system but shielded to anyone but the system administration by G. Antony and A. Rahn. However, data analyses will be based on pseudonyms.

The personal data as well as the anonymously obtained data in the study and the pseudonymised data will be stored at the archive of the University Medical Center Hamburg-Eppendorf (UKE) for a period of ten years.

Access to the original data and pseudonymised data is only granted to authorised employees of Institute of Neuroimmunology and Multiple Sclerosis. On request, the pseudonymised data can be viewed for traceability and further research.

7 Ethics and dissemination

7.1 Consent or assent

To take part in the study, each potential participant will be fully informed (orally or in written form) of the aims and requirements of the study, before deciding whether to take part in the study. In case of revoked consent, pseudonymised data will be anonymised and used in this form. A deletion of already anonymized or anonymously obtained data is not possible.

Additionally, written consent has to be obtained by a physician or trained scientific staff in the partaking MS/SLE clinics and MS/SLE practices for the face-to-face encounters or telephone interviews (by email, fax, directly). Concerning the web-based surveys, the participants have to give informed consent online in order to use UNIPARK or LimeSurvey. The consent is specified by the Declaration of Helsinki, and by the GCP Guidelines of the EU.

7.2 Protocol amendment

Possible protocol amendments will be reported to all relevant ethical committees and approval will be sought for at the University Hamburg.

7.3 Confidentiality

All personal information will be pseudonymised and there will be no possibility to link data to persons without access to a separate code list.

Further, with regard to publications, there will be used only anonymized data.

7.4 Data monitoring

Negative effects on the quality of life or other adverse events due to the information provision and/ or counselling on motherhood choice are not expected. Further, there are no audits planned.

7.5 Declaration of interests

CH has received research grants, congress travel compensations as well as salaries for talks from Biogenc, Genzyme, Sanofi-Aventis, Bayer Healthcare, Merck, Roche, Teva Pharma and Novartis.

SMG has received honoraria from Almirall S.A., Mylan GmbH, and Celgene; research grants from Biogen and in-kind research support from GAIA Group. His research is funded by grants from the Deutsche Forschungsgemeinschaft, Bundesministerium für Bildung und Forschung, and the National MS Society.

AR was funded by German Ministry of Education and Research and received sponsorship from the University of Hamburg (equalisation fund) and is supported by a research grant from the National MS Society, USA (grant no.G-1508-06034).

JP is funded by the Deutsche Forschungsgemeinschaft (DFG, German Research Foundation).

7.6 Access to data

The study centre will coordinate the intra-study data sharing process. All investigators will get access to qualitative data as well as the cleaned data sets.

7.7 Dissemination policy

The results of this mixed methods study will be published in three steps in open access peer-reviewed journals. The first publication will contain the results of the systematic review and the qualitative studies and the second of the web-based survey. Finally, the third publication will be carried out after the pilot of the decision support tool and the decision coaching programme.

Additionally, the study results will be presented on patient websites, e.g. a summary of the results of the web-based survey on the website of DMSG. Furthermore, the results presented at scientific congresses and conferences.

8 Authorship eligibility guidelines

Authorship will be shared between persons involved in the study following the current guidelines of the International Committee of Medical Journal Editors (ICMJE). No professional writers and no persons not directly involved in the study will be granted authorship.

9 Timetable and Milestones

Milestones:

Phase 1 – Development of the outcome measures and the intervention prototypes

- Systematic reviews
- Qualitative interview studies
- Development of the patient reported outcome measures (MCKQ-R and MPWQ)
- Pilot of MCKQ-R and MPWQ (cross-sectional studies)
- Web-based surveys (cross-sectional studies)
- Development of the decision support tool (EBPI and DA)
- Development of the decision coaching programme (EBPI and Coaching)

Phase 2 – Feasibility testing of the decision support tool and the decision coaching programme and multinational survey

- Multinational Survey
- Qualitative feasibility testing of the decision support tool
- Qualitative feasibility testing of the decision coaching programme
- Randomised pilot

Table 1: Gant chart of “motherhood choice in SLE/MS – a mixed methods study”

**Date**	Oct - Dec 2018	Jan – Dec 2019				Jan – Dec 2020				Jan – Dec 2021			
**Tasks / Months**	4. Quartal	1. Quartal	2. Quartal	3. Quartal	4. Quartal	1. Quartal	2. Quartal	3. Quartal	4. Quartal	1. Quartal	2. Quartal	3. Quartal	4. Quartal
**Phase 1 - Development of the outcome measures and the intervention prototypes**													
**Study Protocol and application for ethical approval**													
**Systematic reviews**													
**Qualitative interview study**													
Dissemination / Publication													
**Patient reported outcome measures (PROMs)**													
Development of the PROMs (MCKQ-R and MPWQ)													
MCKQ and MPWQ piloting (cross-sectional study)													
**Web-based survey** (cross-sectional study)													
Dissemination / Publication													
**Development of the Decision Support Tool (DST)**													
**Development of the Decision Coaching Programme (DCP)**													

**Date**	Oct - Dec 2018	Jan – Dec 2019				Jan – Dec 2020				Jan – Dez 2021			
**Tasks / Months**	4. Quartal	1. Quartal	2. Quartal	3. Quartal	4. Quartal	1. Quartal	2. Quartal	3. Quartal	4. Quartal	1. Quartal	2. Quartal	3. Quartal	4. Quartal
**Phase 2 – Feasibility testing of the DST and the DCP and multinational survey**													
**Multinational Survey**													
**Qualitative feasibility testing of the DST**													
**Qualitative feasibility testing of the DCP**													
**Randomised Pilot**													
Data analysis													
Dissemination / Publication													

10 References

Abhyankar, P., Volk, R. J., Blumenthal-Barby, J., Bravo, P., Buchholz, A., Ozanne, E., . . . Stalmeier, P. (2013). Balancing the presentation of information and options in patient decision aids: an updated review. *BMC Med Inform Decis Mak, 13 Suppl 2*, S6. doi:10.1186/1472-6947-13-S2-S6

Ajzen, I. (2011). The theory of planned behaviour: reactions and reflections. *Psychol Health, 26*(9), 1113–1127. doi:10.1080/08870446.2011.613995

Alroughani, R., Altintas, A., Al Jumah, M., Sahraian, M., Alsharoqi, I., AlTahan, A., . . . Bohlega, S. (2016). Pregnancy and the Use of Disease-Modifying Therapies in Patients with Multiple Sclerosis: Benefits versus Risks. *Multiple sclerosis international, 2016*, 1034912. doi:10.1155/2016/1034912

Andreoli, L., Bertsias, G. K., Agmon-Levin, N., Brown, S., Cervera, R., Costedoat-Chalumeau, N., . . . Tincani, A. (2017). EULAR recommendations for women's health and the management of family planning, assisted reproduction, pregnancy and menopause in patients with systemic lupus erythematosus and/or antiphospholipid syndrome. *Annals of the rheumatic diseases, 76*(3), 476–485. doi:10.1136/annrheumdis-2016-209770

Arain, M., Campbell, M. J., Cooper, C. L., & Lancaster, G. A. (2010). What is a pilot or feasibility study? A review of current practice and editorial policy. *BMC Med Res Methodol, 10*, 67. doi:10.1186/1471-2288-10-67

Bowen, D. J., Kreuter, M., Spring, B., Cofta-Woerpel, L., Linnan, L., Weiner, D., . . . Fernandez, M. (2009). How We Design Feasibility Studies. *Am J Prev Med, 36*(5), 452–457. doi:10.1016/j.amepre.2009.02.002

Braun, B., & Marstedt, G. (2014). Partizipative Entscheidungsfindung beim Arzt. Anspruch und Wirklichkeit. *Auszug aus „Gesundheitsmonitor 2014“.* Retrieved from https://wwwbertelsmannstiftung.de/fileadmin/files/BSt/Publikationen/GrauePublikationen/VV-PmW-PEF.pdf

Braun, V., & Clarke, V. (2014). What can "thematic analysis" offer health and wellbeing researchers? *Int J Qual Stud Health Well-being, 9*, 26152. doi:10.3402/qhw.v9.26152

Brinks, R., Fischer-Betz, R., Sander, O., Richter, J. G., Chehab, G., & Schneider, M. (2014). Age-specific prevalence of diagnosed systemic lupus erythematosus in Germany 2002 and projection to 2030. *Lupus, 23*(13), 1407-1411. doi:10.1177/0961203314540352

Browne, P., Chandraratna, D., Angood, C., Tremlett, H., Baker, C., Taylor, B. V., & Thompson, A. J. (2014). Atlas of Multiple Sclerosis 2013: A growing global problem with widespread inequity. *83*(11), 1022-1024. doi:10.1212/WNL.0000000000000768 %J Neurology

Buber, R. (2009). Denke-Laut-Protokolle. *Qualitative Marktforschung*, 555–568.

Buchholz, A., Hölzel L, Kriston L, Simon D, Härter M. (2011). Die Decisional Conflict Scale in deutscher Sprache (DCS-D) - Dimensionale Struktur in einer Stichprobe von Hausarztpatienten. *Klin. Diagnostik u. Evaluation, 4*(1), 15–30.

Bühner, M. (2006). *Einführung in die Test- und Fragebogenkonstruktion* (2., aktualisierte und erw. Aufl. ed.). München: Pearson Studium.

Bunge, M., Mühlhauser, I., & Steckelberg, A. (2010). What constitutes evidence-based patient information?: Overview of discussed criteria. *Patient Educ Couns, 78*(3), 316–328. doi:10.1016/j.pec.2009.10.029

Clowse, M. E., Chakravarty, E., Costenbader, K. H., Chambers, C., & Michaud, K. (2012). Effects of infertility, pregnancy loss, and patient concerns on family size of women with rheumatoid arthritis and systemic lupus erythematosus. *Arthritis Care Res (Hoboken), 64*(5), 668-674. doi:10.1002/acr.21593

Colombo, C., Mosconi, P., Confalonieri, P., Baroni, I., Traversa, S., Hill, S. J., . . . Filippini, G. (2014). Web search behavior and information needs of people with multiple sclerosis: focus group study and analysis of online postings. *Interact J Med Res, 3*(3), e12. doi:10.2196/ijmr.3034

Craig, P., Dieppe, P., Macintyre, S., Michie, S., Nazareth, I., Petticrew, M., & Medical Research Council, G. (2008). Developing and evaluating complex interventions: the new Medical Research Council guidance. *Brit Med J, 337*, a1655. doi:10.1136/bmj.a1655

Creswell, J., & Plano Clark, V. (2011). *Designing and conducting mixed methods research* (2 ed.). Thousand Oaks: SAGE.

DeWalt, D. A., Broucksou, K. A., Hawk, V., Brach, C., Hink, A., Rudd, R., & Callahan, L. (2011). Developing and testing the health literacy universal precautions toolkit. *NURSING OUTLOOK, 59*(2), 85–94. doi:10.1016/j.outlook.2010.12.002

Degner, L. F., Sloan, J. A., & Venkatesh, P. (1997). The control preferences scale. *Canadian Journal of Nursing Research Archive*, 29(3).

Drug, & Therapeutics, B. (2013). An introduction to patient decision aids. *BMJ, 347*, f4147. doi:10.1136/bmj.f4147

Eldridge, S. M., Lancaster, G. A., Campbell, M. J., Thabane, L., Hopewell, S., Coleman, C. L., & Bond, C. M. (2016). Defining Feasibility and Pilot Studies in Preparation for Randomised Controlled Trials: Development of a Conceptual Framework. *PLoS One, 11*(3), e0150205. doi:10.1371/journal.pone.0150205

Elwyn, G., Edwards, A., Wensing, M., Hibbs, R., Wilkinson, C., & Grol, R. (2001a). Shared decision making observed in clinical practice: visual displays of communication sequence and patterns. *J Eval Clin Pract, 7*(2), 211–221.

Elwyn, G., Edwards, A., Wensing, M., Hibbs, R., Wilkinson, C., & Grol, R. (2001b). Shared decision making observed in clinical practice: visual displays of communication sequence and patterns. *J Eval Clin Pract, 7*(2), 211-221.

Ernst, M., Brähler, E., Wild, P.S., Faber, J.,Merzenich, H., & Beutel, M.E. (2020). The desire for children among adult survivors of childhood cancer: Psychometric evaluation of a cancer-specific questionnaire and relations with sociodemographic and psychological characteristics. *Psycho-Oncology*, 29, 485–492. https://doi.org/10.1002/pon.5285

Eysenbach, G. (2004). Improving the quality of Web surveys: the Checklist for Reporting Results of Internet E-Surveys (CHERRIES). *J Med Internet Res, 6*(3), e34. doi:10.2196/jmir.6.3.e34

Finkelsztejn, A., Brooks, J. B., Paschoal, F. M., Jr., & Fragoso, Y. D. (2011). What can we really tell women with multiple sclerosis regarding pregnancy? A systematic review and meta-analysis of the literature. *Bjog, 118*(7), 790-797. doi:10.1111/j.1471-0528.2011.02931.x

Gold, S. M., Heesen, C., Schulz, H., Guder, U., Mönch, A., Gbadamosi, J., ... & Schulz, K. H. (2001). Disease specific quality of life instruments in multiple sclerosis: validation of the Hamburg Quality of Life Questionnaire in Multiple Sclerosis (HAQUAMS). *Multiple Sclerosis Journal*, *7*(2), 119-130.

Greenhalgh, T., Snow, R., Ryan, S., Rees, S., & Salisbury, H. (2015). Six 'biases' against patients and carers in evidence-based medicine. *BMC Med, 13*, 200. doi:10.1186/s12916-015-0437-x

Haase, R., Schultheiss, T., Kempcke, R., Thomas, K., & Ziemssen, T. (2012). Use and Acceptance of Electronic Communication by Patients With Multiple Sclerosis: A Multicenter Questionnaire Study. *Journal of medical Internet research, 14*(5). doi:10.2196/jmir.2133

Herndon, R. e. a. (2004). Pregnancy and MS

A Roundtable Discussion. *Int J MS Care*, 140-143.

Holstiege, J., Steffen, A., Goffrier, B., & Bätzing-Feigenbaum, J. (2017). *Epidemiologie der Multiplen Sklerose-eine populationsbasierte deutschlandweite Studie* (Vol. Versorgungsatlas-Report).

International Patient Decision Aids Standards Collaboration. (2019). IPDAS. Retrieved from http://ipdas.ohri.ca/

Kasper, J., Hoffmann, F., Heesen, C., Köpke, S., & Geiger, F. (2012). Completing the third person's perspective on patients' involvement in medical decision-making: approaching the full picture. *Z Evid Fortbild Qual Gesundhwes, 106*(4), 275–283. doi:10.1016/j.zefq.2012.04.005

Koch-Henriksen, N., & Sorensen, P. S. (2010). The changing demographic pattern of multiple sclerosis epidemiology. *Lancet Neurol, 9*(5), 520-532. doi:10.1016/s1474-4422(10)70064-8

Lazzaroni, M. G., Dall'Ara, F., Fredi, M., Nalli, C., Reggia, R., Lojacono, A., . . . Tincani, A. (2016). A comprehensive review of the clinical approach to pregnancy and systemic lupus

erythematosus. *Journal of Autoimmunity, 74*, 106-117. doi:https://doi.org/10.1016/j.jaut.2016.06.016

Limesurvey GmbH. LimeSurvey: An Open Source survey tool. LimeSurvey GmbH, Hamburg, Germany. Retrieved from http://www.limesurvey.org

Lorefice, L., Mura, G., Coni, G., Fenu, G., Sardu, C., Frau, J., . . . Cocco, E. (2013). What do multiple sclerosis patients and their caregivers perceive as unmet needs? *BMC Neurol, 13*, 177. doi:10.1186/1471-2377-13-177

Lorefice, L., Mura, G., Coni, G., Fenu, G., Sardu, C., Frau, J., . . . Cocco, E. (2013). What do multiple sclerosis patients and their caregivers perceive as unmet needs? *BMC neurology, 13*. doi:10.1186/1471-2377-13-177

Lühnen J., A. M., Mühlhauser I., Steckelberg A. (2017). Leitlinie evidenzbasierte Gesundheitsinformation. Retrieved from https://www.leitlinie-gesundheitsinformation.de/

Marrie, R. A., Salter, A. R., Tyry, T., Fox, R. J., & Cutter, G. R. (2013). Preferred Sources of Health Information in Persons With Multiple Sclerosis: Degree of Trust and Information Sought. *Journal of medical Internet research, 15*(4). doi:10.2196/jmir.2466

McGrath, A., Sharpe, L., Lah, S., & Parratt, K. (2017). Evaluation of a Decision Aid for Women with Epilepsy Who Are Considering Pregnancy: A Randomized Controlled Trial. *Med Decis Making, 37*(5), 589-599. doi:10.1177/0272989x17697304

Meade, T., Dowswell, E., Manolios, N., & Sharpe, L. (2015). The motherhood choices decision aid for women with rheumatoid arthritis increases knowledge and reduces decisional conflict: a randomized controlled trial. *BMC Musculoskelet Disord, 16*, 260. doi:10.1186/s12891-015-0713-0

Miller, D. H., Chard, D. T., & Ciccarelli, O. (2012). Clinically isolated syndromes. *Lancet Neurol, 11*(2), 157–169.

Moore, G. F., Audrey, S., Barker, M., Bond, L., Bonell, C., Hardeman, W., . . . Baird, J. (2015). Process evaluation of complex interventions: Medical Research Council guidance. *Brit Med J, 350*, h1258. doi:10.1136/bmj.h1258

Moroni, G., & Ponticelli, C. (2016). Pregnancy in women with systemic lupus erythematosus (SLE). *Eur J Intern Med, 32*, 7-12. doi:10.1016/j.ejim.2016.04.005

Mudra, S., Göbel, A., Barthel, D., Hecher, K., Schulte-Markwort, M., Goletzke, J., ... & Diemert, A. (2019). Psychometric properties of the German version of the pregnancy-related anxiety questionnaire-revised 2 (PRAQ-R2) in the third trimester of pregnancy. *BMC Pregnancy and Childbirth*, *19*(1), 242.

No author. (2013). *Gesetz zur Verbesserung der Rechte von Patientinnen und Patienten*. Retrieved from http://www.bgbl.de/xaver/bgbl/start.xav?start=//*%5B@attr_id=%27bgbl113s2855.pdf%27%5D#__bgbl__%2F%2F*%5B%40attr_id%3D%27bgbl113s0277.pdf%27%5D__1474360992463.

O'Connor, A. M., Stacey, D., Rovner, D., Holmes-Rovner, M., Tetroe, J., Llewellyn-Thomas, H., . . . Jones, J. (2001). Decision aids for people facing health treatment or screening decisions. *Cochrane Database Syst Rev*(3), CD001431. doi:10.1002/14651858.CD001431

Payne, D., & McPherson, K. M. (2010). Becoming mothers. Multiple sclerosis and motherhood: a qualitative study. *Disability and rehabilitation, 32*(8), 629–638. doi:10.3109/09638280903204708

Prunty, M., Sharpe, L., Butow, P., & Fulcher, G. (2008). The motherhood choice: themes arising in the decision-making process for women with multiple sclerosis. *Mult Scler, 14*(5), 701–704. doi:10.1177/1352458507086103

Prunty, M. C., Sharpe, L., Butow, P., & Fulcher, G. (2008). The motherhood choice: a decision aid for women with multiple sclerosis. *Patient Educ Couns, 71*(1), 108–115. doi:10.1016/j.pec.2007.10.021

Rahn, A. C., Köpke, S., Backhus, I., Kasper, J., Anger, K., Untiedt, B., . . . Heesen, C. (2018). Nurse-led immunotreatment DEcision Coaching In people with Multiple Sclerosis (DECIMS) - Feasibility

testing, pilot randomised controlled trial and mixed methods process evaluation. *Int J Nurs Stud, 78*, 26-36. doi:10.1016/j.ijnurstu.2017.08.011

Roth, H. (1971). *Pädagogische Psychologie des Lehrens und Lernens* (13 ed.). Hannover: Hermann Schroedel Verlag KG.

Sackett, D. L., Rosenberg, W. M., Gray, J. A., Haynes, R. B., & Richardson, W. S. (1996). Evidence based medicine: what it is and what it isn't. *Brit Med J, 312*(7023), 71–72.

Solari, A., Giordano, A., Kasper, J., Drulovic, J., van Nunen, A., Vahter, L., . . . on behalf of the Auto, M. S. p. (2013). Role Preferences of People with Multiple Sclerosis: Image-Revised, Computerized Self-Administered Version of the Control Preference Scale. *PLoS One, 8*(6), e66127. doi:10.1371/journal.pone.0066127

Stacey, D., Kryworuchko, J., Belkora, J., Davison, B. J., Durand, M. A., Eden, K. B., . . . Street, R. L., Jr. (2013). Coaching and guidance with patient decision aids: A review of theoretical and empirical evidence. *BMC Med Inform Decis Mak, 13 Suppl 2*, S11. doi:10.1186/1472-6947-13-s2-s11

Stacey, D., Legare, F., Col, N. F., Bennett, C. L., Barry, M. J., Eden, K. B., . . . Wu, J. H. (2014). Decision aids for people facing health treatment or screening decisions. *Cochrane Database Syst Rev, 1*, CD001431. doi:10.1002/14651858.CD001431.pub4

Stacey, D., Macartney, G., Carley, M., Harrison, M. B., & Costars, T. P. (2013). Development and evaluation of evidence-informed clinical nursing protocols for remote assessment, triage and support of cancer treatment-induced symptoms. *Nurs Res Pract, 2013*, 171872. doi:10.1155/2013/171872

Stacey, D., Murray, M. A., Legare, F., Sandy, D., Menard, P., & O'Connor, A. (2008). Decision coaching to support shared decision making: a framework, evidence, and implications for nursing practice, education, and policy. *Worldviews Evid Based Nurs, 5*(1), 25–35. doi:10.1111/j.1741-6787.2007.00108.x

Stiggelbout, A. M., Pieterse, A. H., & De Haes, J. C. J. M. (2015). Shared decision making: Concepts, evidence, and practice. *Patient Educ Couns, 98*(10), 1172-1179. doi:https://doi.org/10.1016/j.pec.2015.06.022

Thabane, L., Hopewell, S., Lancaster, G. A., Bond, C. M., Coleman, C. L., Campbell, M. J., & Eldridge, S. M. (2016). Methods and processes for development of a CONSORT extension for reporting pilot randomized controlled trials. *Pilot and Feasibility Studies, 2*(1), 25. doi:10.1186/s40814-016-0065-z

Thompson, A. J., Banwell, B. L., Barkhof, F., Carroll, W. M., Coetzee, T., Comi, G., . . . Cohen, J. A. (2018). Diagnosis of multiple sclerosis: 2017 revisions of the McDonald criteria. *Lancet Neurol, 17*(2), 162-173. doi:10.1016/s1474-4422(17)30470-2

Thone, J., Thiel, S., Gold, R., & Hellwig, K. (2017). Treatment of multiple sclerosis during pregnancy - safety considerations. *Expert Opin Drug Saf, 16*(5), 523-534. doi:10.1080/14740338.2017.1311321

UNIPARK & QuestBack. (2019). Unipark.   Retrieved from https://www.unipark.com/

Vukusic, S., & Marignier, R. (2015). Multiple sclerosis and pregnancy in the 'treatment era'. *Nat Rev Neurol, 11*(5), 280-289. doi:10.1038/nrneurol.2015.53

11 Appendices

Appendix 1: Lead investigators in the participating centres

Christoph Heesen (CH), Prof. Dr.
University Medical Center Hamburg-Eppendorf
Email: heesen@uke.de

PD Dr med. Kerstin Hellwig
St. Josef-Hospital
Email: k.hellwig@klinikum-bochum.de

PD Dr. med. Rebecca Fischer-Betz
Universitätsklinikum Düsseldorf
rebecca.fischer@med.uni-duesseldorf.de

12 Signature Sheet

Herewith, I confirm that I have read the study protocol carefully and declare my consent with it. I will treat and examine the patients in accordance with the study protocol, the national applicable laws, the international guidelines on good clinical practice (ICH-GCP), and the declaration of Helsinki.

Signature: ______________________________________

Name in print: **Anne Christin Rahn**

Date: **02.03.2021**
