# Supplementary material for: Motherhood choice in multiple sclerosis (MoMS) – Pilot trial of web-based decision support
Source: PLoS One. 2026 Jun 12;21(6):e0351108. doi: 10.1371/journal.pone.0351108 (PMC13262864; doi:10.1371/journal.pone.0351108)
Supplement: Attachment — Submitted filename: Response to Reviewers.dotx [file pone.0351108.s010.dotx]

**Point-by-point response**

*Dear Editor, dear Reviewers,*

*Thank you for the constructive review of our manuscript. We are very grateful for the opportunity to submit a revised version. The valuable comments helped us to improve our manuscript.*

*Below, we provide a detailed point-by-point response to the comments raised by the editorial team and reviewers.*

*We are grateful for the Editor’s comment to check the journal’s style requirements. We have revised the manuscript to meet the style requirements, including adjustments to heading styles and the reformatting and re-uploading of all figures to meet the specified standards. In accordance with the journal’s submission guideline, Tables 2 and 3 have been reformatted and are now presented as figures (Figs 6 and 7), as they contain complex graphical elements.*

*We believe that these revisions will further increase the quality of our paper so that it meets the high standards for publication in PLOS ONE.*

*Best wishes*

*Lara Stahl and Julia Hickstein*

**Journal Requirements:**

#1 Please ensure that your manuscript meets PLOS ONE's style requirements, including those for file naming. The PLOS ONE style templates can be found at

<https://journals.plos.org/plosone/s/file?id=wjVg/PLOSOne_formatting_sample_main_body.pdf> and

[https://journals.plos.org/plosone/s/file?id=ba62/PLOSOne_formatting_sample_title_authors_affiliations.pdf](https://track.editorialmanager.com/CL0/https:%2F%2Fjournals.plos.org%2Fplosone%2Fs%2Ffile%3Fid=ba62%2FPLOSOne_formatting_sample_title_authors_affiliations.pdf/1/010f019cdb44a302-c003fe04-b02d-43fd-8f7c-5e298bf494dc-000000/p9zLcfE-wa_Er3wLTzRbIeU1Sj20sQUgHQFq0qVUNDA=253)

*Thank you. We have carefully revised the manuscript to ensure that it meets PLOS ONE's style requirements.*

#2 We note that you have indicated that there are restrictions to data sharing for this study. For studies involving human research participant data or other sensitive data, we encourage authors to share de-identified or anonymized data. However, when data cannot be publicly shared for ethical reasons, we allow authors to make their data sets available upon request. For information on unacceptable data access restrictions, please see <http://journals.plos.org/plosone/s/data-availability#loc-unacceptable-data-access-restrictions>.

*We have checked the data sharing restrictions. Anonymized data can be made available upon reasonable request. Thus ,we have updated the data availability statement: “The data set analysed during the current study is not publicly available due to privacy issues, but anonymised data are available from AR upon reasonable request.”*

#3 We note that the original protocol file you uploaded contains a confidentiality notice indicating that the protocol may not be shared publicly or be published. Please note, however, that the PLOS Editorial Policy requires that the original protocol be published alongside your manuscript in the event of acceptance. Please note that should your paper be accepted, all content including the protocol will be published under the Creative Commons Attribution (CC BY) 4.0 license, which means that it will be freely available online, and any third party is permitted to access, download, copy, distribute, and use these materials in any way, even commercially, with proper attribution.

Therefore, we ask that you please seek permission from the study sponsor or body imposing the restriction on sharing this document to publish this protocol under CC BY 4.0 if your work is accepted. We kindly ask that you upload a formal statement signed by an institutional representative clarifying whether you will be able to comply with this policy. Additionally, please upload a clean copy of the protocol with the confidentiality notice (and any copyrighted institutional logos or signatures) removed.

*We have obtained the permission to publish this protocol under CC BY 4.0. We have uploaded a clean version of the protocol, along with a signed formal statement regarding its publication.*

#4 We note that the original protocol that you have uploaded as a Supporting Information file contains an institutional logo. As this logo is likely copyrighted, we ask that you please remove it from this file and upload an updated version upon resubmission.

*We have removed the logos and signatures from the study protocol (see clean version of the protocol).*

**Reviewers' comments**

**Reviewer 1**

#1 The study is a simple designed pilot with an imbalanced randomization. The investigators did not conduct a formal sample size calculation due to the exploratory design of their study. For intermediate effect sizes between 0.4 and 0.6 and an 80% confidence level they reference a recommended minimum sample size of 25 to 35 participants for pilot studies . They thus aimed for a sample size of 64 participants (PtDA=48, DC=16) applying a 3:1 randomization. Recruitment challenges dictated otherwise, but the sample is adequate for a pilot investigation. The randomization was conducted appropriately.

*Reply #1: Thank you for your positive and thoughtful summary of our study design, sample size rationale, and randomisation procedure. We have made minor improvements in the manuscript to clarify and make the description of the randomisation sequence change more understandable: “Halfway through the recruitment phase, we changed to a 1:1 randomisation (block sizes of 2, 4, and 6) due to recruitment difficulties to ensure an appropriate number of participants in both groups (PtDA n>20, DC n>10)” (page 9).*

#2 There were missing values. The investigators noted that If fewer than 20% of the items were left unanswered, they used mean value imputation to replace missing values. This can be problematic. It is simple but does not preserve the relationship among variables. Criticisms will arise. It is suggested that they consult a statistician for more accepted methodologies even with the small sample.

*Reply #2: Thank you for your suggestion and comment. We have now consulted a statistician, NE mentioned in the acknowledgement, to check our methodical approach. We (including the consulted statistician) agree that single mean imputation does not preserve the relationships among variables. The “state of the art”-technique would be multiple imputation, which is quite an elaborate technique for a pilot study. We considered the advantages and disadvantages of a range of possible options:*

1) *Exclusion of persons with missing data*
2) *Single mean imputation*
3) *Single regression imputation*
4) *Multiple imputation*
5) *Combination: Exclusion of persons with > 20% missing data and single mean imputation otherwise*

*We settled for the last option. We believe that our approach is sufficient for this exploratory study; the exclusion of questionnaires with more than 20% missings makes sure that the p-value is at most negligibly affected by the ignorance of uncertainty due to missingness that is characteristic of single imputation, while most information from observations is retained in the analysis by including questionnaires with <= 20% missings.*

*If the mean imputed value is far from the true value, our conclusion could be incorrect and there might be an advantage in favour of DC or PtDA. To address this potential bias due to the missing data imputation, a sensitivity analysis was conducted to check how results change 1) if missing values were least favouring DC and 2) if missing values were least favouring PtDA. Specifically, we did the following:*

1) *Every missing item in all questionnaires for the DC group was imputed with the worst observed value for this item in the total study population, and in the PtDA group with the best observed value in the total study population.*
2) *Every missing item in all questionnaires of the DC group was imputed with the best observed value for this item in the total study population, and in the PtDA group with the worst observed value in the total study population.*

*For both cases, explorative group comparisons (Mann-Whitney U test) did not indicate no differences between wwMS in the DC and PtDA groups regarding the MPWQ score.We now added more detailed information on the number of excluded questionnaires and the number of missing items (see page 12 and page 17). We did not include this sensitivity analysis in the manuscript because the number of imputations is very small, and as this is hopefully now clear to the reader. And we do not expect anyone to seriously expect a major impact of the imputation on the results.*

#3 The analysis is non parametric and is acceptable for the small sample once the concern in the above paragraph is addressed. They compared the two groups regarding decisional conflict (DCS), knowledge (MCKQ), and worries (MPWQ) using the Mann-Whitney U test. The conclusions noted in Table 2 follow from the analysis acknowledging the small sample and should be interpreted with caution as the investigators note in the limitation section of the Discussion.

*Reply #3:Thank you for your comment. We agree that the findings should be interpreted with caution. Therefore, we acknowledge this limitation in the discussion section as well as highlight the explorative nature of our analysis in the methods and results section by using the term “exploratory group comparison”. The statistician we consulted considered this methodological approach to be appropriate. In addition, the limitation is now also noted in the legend of Table 2.*

**Reviewer 2**

#1 Thank you very much for the opportunity to review this interesting manuscript titled ‘Motherhood choice in multiple sclerosis (MoMS) – pilot trial of web-based decision support’, to test the decisional conflict for a patient decision aid (PtDA) and a nurse-led decision coaching intervention (DC) developed for supporting motherhood choices in women with multiple sclerosis.
Please see the following comments and suggestions to be addressed by the authors.

*Reply #1: Thank you for reviewing our manuscript, and for your helpful suggestions and comments.*

#2 Abstract: The Methods section mentions about switching to a randomization ratio of 1:1. Please give information about the number of participants finally decided to be involved in the trial.

*Reply #2: Thank you for your comment. As suggested, we have added information on the final number of participants to be involved in the pilot study to the abstract.*

#3 Abstract: The study population and the study setting need to be specified in the Methods section in the abstract.

*Reply #3: As suggested, we have added information on the study population and setting to the abstract.*

#4 Abstract: The Methods section mentions about ‘applying qualitative (interviews) and quantitative methods (questionnaires)’ for data collection. Please elaborate on this to briefly mention which questionnaires were applied, what was assessed using interviews and among whom these were carried out.

*Reply #4: Thank you for your comment. As suggested, we have added details on the included questionnaire and the scope of the interviews.*

#5 Abstract: The Results section mentions about recruiting 22 participants for the PtDA and 13 for the DC. This seems quite a large difference considering the 1:1 randomization ratio mentioned in the Methods section. Please clarify this.

*Reply #5: See also comment 1 to reviewer 1. We had initially intended a 3:1 randomisation, but changed this later in the process to a 1:1 randomisation due to recruitment difficulties. The aim was to randomise a sufficient number of women into the DC group during the remaining recruitment period. There was a risk that we would have collected not enough data on the DC group by the end of the recruitment period. A lower number of participants in the PtDA group did not affect the ability to answer the main feasibility questions. We have optimised the sentence in the abstract to clarify this, while this change in the recruitment strategy was is mentioned in the methods section on page 9. We have revised the sentence in the methods section to clarify and make the description of the randomisation sequence change more understandable: “Halfway through the recruitment phase, we changed to a 1:1 randomisation (block sizes of 2, 4, and 6) due to recruitment difficulties to ensure an appropriate number of participants in both groups (PtDA n>20, DC n>10).”*

#6 Abstract: The results section also mentions that ‘satisfaction levels seemed to be higher in the DC group’ based on the interviews. Was satisfaction assessed as an outcome in the interviews, and how was it determined based on the interviews conducted?

*Reply #6: This result is based on the interview data, which we now clarified. We also reworded the mentioned sentence, as it does not fit linguistically within the context of qualitative data: “Qualitative findings indicate greater satisfaction levels in the DC group.”*

#7 Methods: Please include the inclusion (and exclusion) criteria used in recruiting the participants.

*Reply #7: Thank you for your comment. We have rewritten the paragraph “Participants and recruitment” in section “Randomised pilot trial (beta testing)”, providing inclusion and exclusion criteria: “We defined the following inclusion criteria: women had to be aged between 18 and 45 years with relapsing-remitting multiple sclerosis (RRMS), clinically isolated syndrome (CIS), or suspected RRMS, and who had not yet fully decided about motherhood. Women with MS who were not facing a decision regarding family planning in connection with their MS were excluded.” (page 8).*

#8 Methods: Please explain/ expand the terms RRMS and CIS, the first time they appear in the text (L.160).

*Reply #8:* We apologise for not having provided full texts and now have clarified the abbreviations.

#9 Methods: Please elaborate on the study setting, as to whether the study was carried out by recruiting participants across the country, or in specified localities.

*Reply #9: Thank you for pointing this out. Women with MS from all over Germany were eligible to participate in the pilot study if they met all the inclusion criteria. We have now described this more clearly in the manuscript: “The study took place across Germany with location-independent participation.” (page 8).*

#10 Methods: Was there a particular reason to have two different Decision Guides (DG and DGPro) for the two interventions? Please clarify this.

*Reply #10: In contrast to the DG, the extended version (DGpro) includes information on prognostic factors for the MS course and allows documenting the individual MS course. We consider it essential that this section of the guide be completed with the trained nurse, as it could leave women feeling uncertain and anxious. We have included this information in the main text in subsection “Decision support programmes” in the Methods (see page 7).*

#11 Results: Please explain the large difference observed in the final numbers of participants included in the two interventions, in the context of a randomization ratio of 1:1 with block randomization.

*Reply #11: As mentioned in reply 5 above as well as reply 1 to reviewer 1, we have adjusted the randomisation ratio from 3:1 to 1:1 due to recruitment problems.* *Once half of the recruitment period had passed, it became clear that we would not be able to recruit a sufficient number of participants per group to compare the two interventions by the end of the study if we maintained a 3:1 ratio. By changing the ratio to 1:1, we aimed to recruit more than 20 participants for the PtDA group and more than 10 for the DC group. We have now clarified this in methods section, paragraph “Randomisation” (page 9).*

#12 Results: The content in L. 296-298 is better suited under section 3.3, which would improve the clarity of the results presented.

*Reply #12: As suggested, we have moved the content from line 296-298 to the subsection “Results regarding decisional conflict and involvement in the decision process” (see page 16).*
