# Supplementary material for: Motherhood choice in multiple sclerosis (MoMS) – Pilot trial of web-based decision support
Source: PLoS One. 2026 Jun 12;21(6):e0351108. doi: 10.1371/journal.pone.0351108 (PMC13262864; doi:10.1371/journal.pone.0351108)
Supplement: Peer review history

# Peer review: Decision Letter 0

**DOI:** 10.1371/journal.pone.0351108.r001

**Authors:** Pasyodun Koralage Buddhika Mahesh

11 Mar 2026

PONE-D-25-68178

Motherhood choice in multiple sclerosis (MoMS) – pilot trial of web-based decision support

PLOS One

Dear Dr. Stahl,

Thank you for submitting your manuscript to PLOS ONE. After careful consideration, we feel that it has merit but does not fully meet PLOS ONE’s publication criteria as it currently stands. Therefore, we invite you to submit a revised version of the manuscript that addresses the points raised during the review process.

Please submit your revised manuscript by Apr 25 2026 11:59PM. If you will need more time than this to complete your revisions, please reply to this message or contact the journal office at plosone@plos.org. When you're ready to submit your revision, log on to https://www.editorialmanager.com/pone/ and select the 'Submissions Needing Revision' folder to locate your manuscript file.

Please include the following items when submitting your revised manuscript:

- A letter that responds to each point raised by the academic editor and reviewer(s). You should upload this letter as a separate file labeled 'Response to Reviewers'.
- A marked-up copy of your manuscript that highlights changes made to the original version. You should upload this as a separate file labeled 'Revised Manuscript with Track Changes'.
- An unmarked version of your revised paper without tracked changes. You should upload this as a separate file labeled 'Manuscript'.

If you would like to make changes to your financial disclosure, please include your updated statement in your cover letter. Guidelines for resubmitting your figure files are available below the reviewer comments at the end of this letter.

If applicable, we recommend that you deposit your laboratory protocols in protocols.io to enhance the reproducibility of your results. Protocols.io assigns your protocol its own identifier (DOI) so that it can be cited independently in the future. For instructions see: https://journals.plos.org/plosone/s/submission-guidelines#loc-laboratory-protocols. Additionally, PLOS ONE offers an option for publishing peer-reviewed Lab Protocol articles, which describe protocols hosted on protocols.io. Read more information on sharing protocols at https://plos.org/protocols?utm_medium=editorial-email&utm_source=authorletters&utm_campaign=protocols.

We look forward to receiving your revised manuscript.

Kind regards,

Pasyodun Koralage Buddhika Mahesh

Academic Editor

PLOS One

Journal Requirements:

When submitting your revision, we need you to address these additional requirements.

1. Please ensure that your manuscript meets PLOS ONE's style requirements, including those for file naming. The PLOS ONE style templates can be found at

https://journals.plos.org/plosone/s/file?id=wjVg/PLOSOne_formatting_sample_main_body.pdf and

https://journals.plos.org/plosone/s/file?id=ba62/PLOSOne_formatting_sample_title_authors_affiliations.pdf

2.We note that you have indicated that there are restrictions to data sharing for this study. For studies involving human research participant data or other sensitive data, we encourage authors to share de-identified or anonymized data. However, when data cannot be publicly shared for ethical reasons, we allow authors to make their data sets available upon request. For information on unacceptable data access restrictions, please see http://journals.plos.org/plosone/s/data-availability#loc-unacceptable-data-access-restrictions.

Before we proceed with your manuscript, please address the following prompts:

a) If there are ethical or legal restrictions on sharing a de-identified data set, please explain them in detail (e.g., data contain potentially identifying or sensitive patient information, data are owned by a third-party organization, etc.) and who has imposed them (e.g., a Research Ethics Committee or Institutional Review Board, etc.). Please also provide contact information for a data access committee, ethics committee, or other institutional body to which data requests may be sent.

b) If there are no restrictions, please upload the minimal anonymized data set necessary to replicate your study findings to a stable, public repository and provide us with the relevant URLs, DOIs, or accession numbers. Please see http://www.bmj.com/content/340/bmj.c181.long for guidelines on how to de-identify and prepare clinical data for publication. For a list of recommended repositories, please see https://journals.plos.org/plosone/s/recommended-repositories. You also have the option of uploading the data as Supporting Information files, but we would recommend depositing data directly to a data repository if possible.

Please update your Data Availability statement in the submission form accordingly.

3. We note that the original protocol file you uploaded contains a confidentiality notice indicating that the protocol may not be shared publicly or be published. Please note, however, that the PLOS Editorial Policy requires that the original protocol be published alongside your manuscript in the event of acceptance. Please note that should your paper be accepted, all content including the protocol will be published under the Creative Commons Attribution (CC BY) 4.0 license, which means that it will be freely available online, and any third party is permitted to access, download, copy, distribute, and use these materials in any way, even commercially, with proper attribution.

Therefore, we ask that you please seek permission from the study sponsor or body imposing the restriction on sharing this document to publish this protocol under CC BY 4.0 if your work is accepted. We kindly ask that you upload a formal statement signed by an institutional representative clarifying whether you will be able to comply with this policy. Additionally, please upload a clean copy of the protocol with the confidentiality notice (and any copyrighted institutional logos or signatures) removed.

4. We note that the original protocol that you have uploaded as a Supporting Information file contains an institutional logo. As this logo is likely copyrighted, we ask that you please remove it from this file and upload an updated version upon resubmission.

5. If the reviewer comments include a recommendation to cite specific previously published works, please review and evaluate these publications to determine whether they are relevant and should be cited. There is no requirement to cite these works unless the editor has indicated otherwise.

[Note: HTML markup is below. Please do not edit.]

Reviewers' comments:

Reviewer's Responses to Questions

**Comments to the Author**

1. Is the manuscript technically sound, and do the data support the conclusions?

The manuscript must describe a technically sound piece of scientific research with data that supports the conclusions. Experiments must have been conducted rigorously, with appropriate controls, replication, and sample sizes. The conclusions must be drawn appropriately based on the data presented.

Reviewer #1: No

Reviewer #2: Yes

**********

2. Has the statistical analysis been performed appropriately and rigorously?

Reviewer #1: No

Reviewer #2: Yes

**********

3. Have the authors made all data underlying the findings in their manuscript fully available?

The PLOS Data policy requires authors to make all data underlying the findings described in their manuscript fully available without restriction, with rare exception (please refer to the Data Availability Statement in the manuscript PDF file). The data should be provided as part of the manuscript or its supporting information, or deposited to a public repository. For example, in addition to summary statistics, the data points behind means, medians and variance measures should be available. If there are restrictions on publicly sharing data—e.g. participant privacy or use of data from a third party—those must be specified.

Reviewer #1: Yes

Reviewer #2: No

**********

4. Is the manuscript presented in an intelligible fashion and written in standard English?

PLOS ONE does not copyedit accepted manuscripts, so the language in submitted articles must be clear, correct, and unambiguous. Any typographical or grammatical errors should be corrected at revision, so please note any specific errors here.

Reviewer #1: Yes

Reviewer #2: Yes

**********

5. Review Comments to the Author

Please use the space provided to explain your answers to the questions above. You may also include additional comments for the author, including concerns about dual publication, research ethics, or publication ethics. (Please upload your review as an attachment if it exceeds 20,000 characters)

Reviewer #1: The study is a simple designed pilot with an imbalanced randomization. The investigators did not conduct a formal sample size calculation due to the exploratory design of their study. For intermediate effect sizes between 0.4 and 0.6 and an 80% confidence level they reference a recommended minimum sample size of 25 to 35 participants for pilot studies . They thus aimed for a sample size of 64 participants (PtDA=48, DC=16) applying a 3:1 randomization. Recruitment challenges dictated otherwise, but the sample is adequate for a pilot investigation. The randomization was conducted appropriately.

There were missing values. The investigators noted that If fewer than 20% of the items were left unanswered, they used mean value imputation to replace missing values. This can be problematic. It is simple but does not preserve the relationship among variables. Criticisms will arise. It is suggested that they consult a statistician for more accepted methodologies even with the small sample.

The analysis is non parametric and is acceptable for the small sample once the concern in the above paragraph is addressed. They compared the two groups regarding decisional conflict (DCS), knowledge (MCKQ), and worries (MPWQ) using the Mann-Whitney U test. The conclusions noted in Table 2 follow from the analysis acknowledging the small sample and should be interpreted with caution as the investigators note in the limitation section of the Discussion.

Reviewer #2: Thank you very much for the opportunity to review this interesting manuscript titled ‘Motherhood choice in multiple sclerosis (MoMS) – pilot trial of web-based decision support’, to test the decisional conflict for a patient decision aid (PtDA) and a nurse-led decision coaching intervention (DC) developed for supporting motherhood choices in women with multiple sclerosis.

Please see the following comments and suggestions to be addressed by the authors.

Abstract:

The Methods section mentions about switching to a randomization ratio of 1:1. Please give information about the number of participants finally decided to be involved in the trial.

The study population and the study setting need to be specified in the Methods section in the abstract.

The Methods section mentions about ‘applying qualitative (interviews) and quantitative methods (questionnaires)’ for data collection. Please elaborate on this to briefly mention which questionnaires were applied, what was assessed using interviews and among whom these were carried out.

The Results section mentions about recruiting 22 participants for the PtDA and 13 for the DC. This seems quite a large difference considering the 1:1 randomization ratio mentioned in the Methods section. Please clarify this.

The results section also mentions that ‘satisfaction levels seemed to be higher in the DC group’ based on the interviews. Was satisfaction assessed as an outcome in the interviews, and how was it determined based on the interviews conducted?

Methods:

Please include the inclusion (and exclusion) criteria used in recruiting the participants.

Please explain/ expand the terms RRMS and CIS, the first time they appear in the text (L.160).

Please elaborate on the study setting, as to whether the study was carried out by recruiting participants across the country, or in specified localities.

Was there a particular reason to have two different Decision Guides (DG and DGPro) for the two interventions? Please clarify this.

Results:

Please explain the large difference observed in the final numbers of participants included in the two interventions, in the context of a randomization ratio of 1:1 with block randomization.

The content in L. 296-298 is better suited under section 3.3, which would improve the clarity of the results presented.

**********

6. PLOS authors have the option to publish the peer review history of their article (what does this mean?). If published, this will include your full peer review and any attached files.

If you choose “no”, your identity will remain anonymous but your review may still be made public.

**Do you want your identity to be public for this peer review?**   For information about this choice, including consent withdrawal, please see our Privacy Policy.

Reviewer #1: No

Reviewer #2: No

**********

[NOTE: If reviewer comments were submitted as an attachment file, they will be attached to this email and accessible via the submission site. Please log into your account, locate the manuscript record, and check for the action link "View Attachments". If this link does not appear, there are no attachment files.]

To ensure your figures meet our technical requirements, please review our figure guidelines: https://journals.plos.org/plosone/s/figures

You may also use PLOS’s free figure tool, NAAS, to help you prepare publication quality figures: https://journals.plos.org/plosone/s/figures#loc-tools-for-figure-preparation.

NAAS will assess whether your figures meet our technical requirements by comparing each figure against our figure specifications.

# Author comment: Author response to Decision Letter 1

**DOI:** 10.1371/journal.pone.0351108.r002

6 May 2026

Dear Reviewers,

thank you for taking the time to evaluate our submission and for your comments and advice. We have carefully reviewed the comments and revised the parts that did not meet your requirements and were pointed out. Attached to this submission, you will find the document named "Response to Reviewers", where we answer all the comments point by point.

I have included our responses in here as well, please find them down below.

Kind regards,

Lara Stahl

Reviewer 1

#1 The study is a simple designed pilot with an imbalanced randomization. The investigators did not conduct a formal sample size calculation due to the exploratory design of their study. For intermediate effect sizes between 0.4 and 0.6 and an 80% confidence level they reference a recommended minimum sample size of 25 to 35 participants for pilot studies . They thus aimed for a sample size of 64 participants (PtDA=48, DC=16) applying a 3:1 randomization. Recruitment challenges dictated otherwise, but the sample is adequate for a pilot investigation. The randomization was conducted appropriately.

Reply #1: Thank you for your positive and thoughtful summary of our study design, sample size rationale, and randomisation procedure. We have made minor improvements in the manuscript to clarify and make the description of the randomisation sequence change more understandable: “Halfway through the recruitment phase, we changed to a 1:1 randomisation (block sizes of 2, 4, and 6) due to recruitment difficulties to ensure an appropriate number of participants in both groups (PtDA n>20, DC n>10)” (page 9).

#2 There were missing values. The investigators noted that If fewer than 20% of the items were left unanswered, they used mean value imputation to replace missing values. This can be problematic. It is simple but does not preserve the relationship among variables. Criticisms will arise. It is suggested that they consult a statistician for more accepted methodologies even with the small sample.

Reply #2: Thank you for your suggestion and comment. We have now consulted a statistician, NE mentioned in the acknowledgement, to check our methodical approach. We (including the consulted statistician) agree that single mean imputation does not preserve the relationships among variables. The “state of the art”-technique would be multiple imputation, which is quite an elaborate technique for a pilot study. We considered the advantages and disadvantages of a range of possible options:

1) Exclusion of persons with missing data

2) Single mean imputation

3) Single regression imputation

4) Multiple imputation

5) Combination: Exclusion of persons with > 20% missing data and single mean imputation otherwise

We settled for the last option. We believe that our approach is sufficient for this exploratory study; the exclusion of questionnaires with more than 20% missings makes sure that the p-value is at most negligibly affected by the ignorance of uncertainty due to missingness that is characteristic of single imputation, while most information from observations is retained in the analysis by including questionnaires with <= 20% missings.

If the mean imputed value is far from the true value, our conclusion could be incorrect and there might be an advantage in favour of DC or PtDA. To address this potential bias due to the missing data imputation, a sensitivity analysis was conducted to check how results change 1) if missing values were least favouring DC and 2) if missing values were least favouring PtDA. Specifically, we did the following:

1) Every missing item in all questionnaires for the DC group was imputed with the worst observed value for this item in the total study population, and in the PtDA group with the best observed value in the total study population.

2) Every missing item in all questionnaires of the DC group was imputed with the best observed value for this item in the total study population, and in the PtDA group with the worst observed value in the total study population.

For both cases, explorative group comparisons (Mann-Whitney U test) did not indicate no differences between wwMS in the DC and PtDA groups regarding the MPWQ score.We now added more detailed information on the number of excluded questionnaires and the number of missing items (see page 12 and page 17). We did not include this sensitivity analysis in the manuscript because the number of imputations is very small, and as this is hopefully now clear to the reader. And we do not expect anyone to seriously expect a major impact of the imputation on the results.

#3 The analysis is non parametric and is acceptable for the small sample once the concern in the above paragraph is addressed. They compared the two groups regarding decisional conflict (DCS), knowledge (MCKQ), and worries (MPWQ) using the Mann-Whitney U test. The conclusions noted in Table 2 follow from the analysis acknowledging the small sample and should be interpreted with caution as the investigators note in the limitation section of the Discussion.

Reply #3:Thank you for your comment. We agree that the findings should be interpreted with caution. Therefore, we acknowledge this limitation in the discussion section as well as highlight the explorative nature of our analysis in the methods and results section by using the term “exploratory group comparison”. The statistician we consulted considered this methodological approach to be appropriate. In addition, the limitation is now also noted in the legend of Table 2.

Reviewer 2

#1 Thank you very much for the opportunity to review this interesting manuscript titled ‘Motherhood choice in multiple sclerosis (MoMS) – pilot trial of web-based decision support’, to test the decisional conflict for a patient decision aid (PtDA) and a nurse-led decision coaching intervention (DC) developed for supporting motherhood choices in women with multiple sclerosis.

Please see the following comments and suggestions to be addressed by the authors.

Reply #1: Thank you for reviewing our manuscript, and for your helpful suggestions and comments.

#2 Abstract: The Methods section mentions about switching to a randomization ratio of 1:1. Please give information about the number of participants finally decided to be involved in the trial.

Reply #2: Thank you for your comment. As suggested, we have added information on the final number of participants to be involved in the pilot study to the abstract.

#3 Abstract: The study population and the study setting need to be specified in the Methods section in the abstract.

Reply #3: As suggested, we have added information on the study population and setting to the abstract.

#4 Abstract: The Methods section mentions about ‘applying qualitative (interviews) and quantitative methods (questionnaires)’ for data collection. Please elaborate on this to briefly mention which questionnaires were applied, what was assessed using interviews and among whom these were carried out.

Reply #4: Thank you for your comment. As suggested, we have added details on the included questionnaire and the scope of the interviews.

#5 Abstract: The Results section mentions about recruiting 22 participants for the PtDA and 13 for the DC. This seems quite a large difference considering the 1:1 randomization ratio mentioned in the Methods section. Please clarify this.

Reply #5: See also comment 1 to reviewer 1. We had initially intended a 3:1 randomisation, but changed this later in the process to a 1:1 randomisation due to recruitment difficulties. The aim was to randomise a sufficient number of women into the DC group during the remaining recruitment period. There was a risk that we would have collected not enough data on the DC group by the end of the recruitment period. A lower number of participants in the PtDA group did not affect the ability to answer the main feasibility questions. We have optimised the sentence in the abstract to clarify this, while this change in the recruitment strategy was is mentioned in the methods section on page 9. We have revised the sentence in the methods section to clarify and make the description of the randomisation sequence change more understandable: “Halfway through the recruitment phase, we changed to a 1:1 randomisation (block sizes of 2, 4, and 6) due to recruitment difficulties to ensure an appropriate number of participants in both groups (PtDA n>20, DC n>10).”

#6 Abstract: The results section also mentions that ‘satisfaction levels seemed to be higher in the DC group’ based on the interviews. Was satisfaction assessed as an outcome in the interviews, and how was it determined based on the interviews conducted?

Reply #6: This result is based on the interview data, which we now clarified. We also reworded the mentioned sentence, as it does not fit linguistically within the context of qualitative data: “Qualitative findings indicate greater satisfaction levels in the DC group.”

#7 Methods: Please include the inclusion (and exclusion) criteria used in recruiting the participants.

Reply #7: Thank you for your comment. We have rewritten the paragraph “Participants and recruitment” in section “Randomised pilot trial (beta testing)”, providing inclusion and exclusion criteria: “We defined the following inclusion criteria: women had to be aged between 18 and 45 years with relapsing-remitting multiple sclerosis (RRMS), clinically isolated syndrome (CIS), or suspected RRMS, and who had not yet fully decided about motherhood. Women with MS who were not facing a decision regarding family planning in connection with their MS were excluded.” (page 8).

#8 Methods: Please explain/ expand the terms RRMS and CIS, the first time they appear in the text (L.160).

Reply #8: We apologise for not having provided full texts and now have clarified the abbreviations.

#9 Methods: Please elaborate on the study setting, as to whether the study was carried out by recruiting participants across the country, or in specified localities.

Reply #9: Thank you for pointing this out. Women with MS from all over Germany were eligible to participate in the pilot study if they met all the inclusion criteria. We have now described this more clearly in the manuscript: “The study took place across Germany with location-independent participation.” (page 8).

#10 Methods: Was there a particular reason to have two different Decision Guides (DG and DGPro) for the two interventions? Please clarify this.

Reply #10: In contrast to the DG, the extended version (DGpro) includes information on prognostic factors for the MS course and allows documenting the individual MS course. We consider it essential that this section of the guide be completed with the trained nurse, as it could leave women feeling uncertain and anxious. We have included this information in the main text in subsection “Decision support programmes” in the Methods (see page 7).

#11 Results: Please explain the large difference observed in the final numbers of participants included in the two interventions, in the context of a randomization ratio of 1:1 with block randomization.

Reply #11: As mentioned in reply 5 above as well as reply 1 to reviewer 1, we have adjusted the randomisation ratio from 3:1 to 1:1 due to recruitment problems. Once half of the recruitment period had passed, it became clear that we would not be able to recruit a sufficient number of participants per group to compare the two interventions by the end of the study if we maintained a 3:1 ratio. By changing the ratio to 1:1, we aimed to recruit more than 20 participants for the PtDA group and more than 10 for the DC group. We have now clarified this in methods section, paragraph “Randomisation” (page 9).

#12 Results: The content in L. 296-298 is better suited under section 3.3, which would improve the clarity of the results presented.

Reply #12: As suggested, we have moved the content from line 296-298 to the subsection “Results regarding decisional conflict and involvement in the decision process” (see page 16).

Attachment Submitted filename: Response to Reviewers.dotx

# Peer review: Decision Letter 1

**DOI:** 10.1371/journal.pone.0351108.r003

**Authors:** Pasyodun Koralage Buddhika Mahesh

25 May 2026

Motherhood choice in multiple sclerosis (MoMS) – pilot trial of web-based decision support

PONE-D-25-68178R1

Dear Dr. Stahl,

We’re pleased to inform you that your manuscript has been judged scientifically suitable for publication and will be formally accepted for publication once it meets all outstanding technical requirements.

Within one week, you’ll receive an e-mail detailing the required amendments. When these have been addressed, you’ll receive a formal acceptance letter and your manuscript will be scheduled for publication.

An invoice will be generated when your article is formally accepted. Please note, if your institution has a publishing partnership with PLOS and your article meets the relevant criteria, all or part of your publication costs will be covered. Please make sure your user information is up-to-date by logging into Editorial Manager at Editorial Manager® and clicking the ‘Update My Information' link at the top of the page. For questions related to billing, please contact billing support.

If your institution or institutions have a press office, please notify them about your upcoming paper to help maximize its impact. If they’ll be preparing press materials, please inform our press team as soon as possible -- no later than 48 hours after receiving the formal acceptance. Your manuscript will remain under strict press embargo until 2 pm Eastern Time on the date of publication. For more information, please contact onepress@plos.org.

Kind regards,

Pasyodun Koralage Buddhika Mahesh

Academic Editor

PLOS One

Additional Editor Comments (optional):

Reviewers' comments:

Reviewer's Responses to Questions

**Comments to the Author**

1. If the authors have adequately addressed your comments raised in a previous round of review and you feel that this manuscript is now acceptable for publication, you may indicate that here to bypass the “Comments to the Author” section, enter your conflict of interest statement in the “Confidential to Editor” section, and submit your "Accept" recommendation.

Reviewer #1: All comments have been addressed

Reviewer #2: All comments have been addressed

**********

2. Is the manuscript technically sound, and do the data support the conclusions?

The manuscript must describe a technically sound piece of scientific research with data that supports the conclusions. Experiments must have been conducted rigorously, with appropriate controls, replication, and sample sizes. The conclusions must be drawn appropriately based on the data presented.

Reviewer #1: (No Response)

Reviewer #2: (No Response)

**********

3. Has the statistical analysis been performed appropriately and rigorously?

Reviewer #1: (No Response)

Reviewer #2: (No Response)

**********

4. Have the authors made all data underlying the findings in their manuscript fully available?

The PLOS Data policy requires authors to make all data underlying the findings described in their manuscript fully available without restriction, with rare exception (please refer to the Data Availability Statement in the manuscript PDF file). The data should be provided as part of the manuscript or its supporting information, or deposited to a public repository. For example, in addition to summary statistics, the data points behind means, medians and variance measures should be available. If there are restrictions on publicly sharing data—e.g. participant privacy or use of data from a third party—those must be specified.

Reviewer #1: (No Response)

Reviewer #2: (No Response)

**********

5. Is the manuscript presented in an intelligible fashion and written in standard English?

PLOS ONE does not copyedit accepted manuscripts, so the language in submitted articles must be clear, correct, and unambiguous. Any typographical or grammatical errors should be corrected at revision, so please note any specific errors here.

Reviewer #1: (No Response)

Reviewer #2: (No Response)

**********

6. Review Comments to the Author

Please use the space provided to explain your answers to the questions above. You may also include additional comments for the author, including concerns about dual publication, research ethics, or publication ethics. (Please upload your review as an attachment if it exceeds 20,000 characters)

Reviewer #1: Al comments have been adequately addressed. Their missing value solution should suffice.

\XXXXXXXXXXXXXXXXXXXXXXXXXXXXXXXXXXXXXXXXXXXXXXXXXXXXXXXXXXXXXXXXXXXXXXXXXXXXXXXXXXXXXXXXXXXXXXXXXXXXXXXXXXXXXXXXXXXXXXXXXXXXXXXXXXXXXXXXXXXXXXXXXXX

Reviewer #2: (No Response)

**********

7. PLOS authors have the option to publish the peer review history of their article (what does this mean?). If published, this will include your full peer review and any attached files.

If you choose “no”, your identity will remain anonymous but your review may still be made public.

**Do you want your identity to be public for this peer review?**   For information about this choice, including consent withdrawal, please see our Privacy Policy.

Reviewer #1: No

Reviewer #2: No

**********

# Editor report: Acceptance letter

**DOI:** 10.1371/journal.pone.0351108.r004

**Authors:** Pasyodun Koralage Buddhika Mahesh

PONE-D-25-68178R1

PLOS One

Dear Dr. Stahl,

I'm pleased to inform you that your manuscript has been deemed suitable for publication in PLOS One. Congratulations! Your manuscript is now being handed over to our production team.

At this stage, our production department will prepare your paper for publication. This includes ensuring the following:

* All references, tables, and figures are properly cited

* All relevant supporting information is included in the manuscript submission,

* There are no issues that prevent the paper from being properly typeset

You will receive further instructions from the production team, including instructions on how to review your proof when it is ready. Please keep in mind that we are working through a large volume of accepted articles, so please give us a few days to review your paper and let you know the next and final steps.

Lastly, if your institution or institutions have a press office, please let them know about your upcoming paper now to help maximize its impact. If they'll be preparing press materials, please inform our press team within the next 48 hours. Your manuscript will remain under strict press embargo until 2 pm Eastern Time on the date of publication. For more information, please contact onepress@plos.org.

You will receive an invoice from PLOS for your publication fee after your manuscript has reached the completed accept phase. If you receive an email requesting payment before acceptance or for any other service, this may be a phishing scheme. Learn how to identify phishing emails and protect your accounts at https://explore.plos.org/phishing.

If we can help with anything else, please email us at customercare@plos.org.

Thank you for submitting your work to PLOS ONE and supporting open access.

Kind regards,

PLOS ONE Editorial Office Staff

on behalf of

Dr. Pasyodun Koralage Buddhika Mahesh

Academic Editor

PLOS One
